# Loss of CorA, the primary magnesium transporter of *Salmonella*, is alleviated by MgtA and PhoP-dependent compensatory mechanisms

**DOI:** 10.1371/journal.pone.0291736

**Published:** 2023-09-15

**Authors:** Selma Metaane, Véronique Monteil, Thibaut Douché, Quentin Giai Gianetto, Mariette Matondo, Corinne Maufrais, Françoise Norel

**Affiliations:** 1 Biochimie des Interactions Macromoléculaires, Institut Pasteur, CNRS UMR3528, Université Paris Cité, Paris, France; 2 Proteomic Platform, Mass Spectrometry for Biology Unit, Institut Pasteur, CNRS UAR 2024, Université Paris Cité, Paris, France; 3 Bioinformatics and Biostatistics Hub, Institut Pasteur, Université Paris Cité, Paris, France; Instituto Butantan, BRAZIL

## Abstract

In many Gram-negative bacteria, the stress sigma factor of RNA polymerase, σ^S^/RpoS, remodels global gene expression to reshape the physiology of stationary phase cells and ensure their survival under non-optimal growth conditions. In the foodborne pathogen *Salmonella enterica* serovar Typhimurium, σ^S^ is also required for biofilm formation and virulence. We have recently shown that a Δ*rpoS* mutation decreases the magnesium content and expression level of the housekeeping Mg^2+^-transporter CorA in stationary phase *Salmonella*. The other two Mg^2+^-transporters of *Salmonella* are encoded by the PhoP-activated *mgtA* and *mgtB* genes and are expressed under magnesium starvation. The σ^S^ control of *corA* prompted us to evaluate the impact of CorA in stationary phase *Salmonella* cells, by using global and analytical proteomic analyses and physiological assays. The Δ*corA* mutation conferred a competitive disadvantage to exit from stationary phase, and slightly impaired motility, but had no effect on total and free cellular magnesium contents. In contrast to the wild-type strain, the Δ*corA* mutant produced MgtA, but not MgtB, in the presence of high extracellular magnesium concentration. Under these conditions, MgtA production in the Δ*corA* mutant did not require PhoP. Consistently, a Δ*mgtA*, but not a Δ*phoP*, mutation slightly reduced the magnesium content of the Δ*corA* mutant. Synthetic phenotypes were observed when the Δ*phoP* and Δ*corA* mutations were combined, including a strong reduction in growth and motility, independently of the extracellular magnesium concentration. The abundance of several proteins involved in flagella formation, chemotaxis and secretion was lowered by the Δ*corA* and Δ*phoP* mutations in combination, but not alone. These findings unravel the importance of PhoP-dependent functions in the absence of CorA when magnesium is sufficient. Altogether, our data pinpoint a regulatory network, where the absence of CorA is sensed by the cell and compensated by MgtA and PhoP- dependent mechanisms.

## Introduction

In many Gram-negative bacteria, the alternative sigma subunit of RNA polymerase, σ^S^/RpoS, remodels global gene expression to reshape the cell physiology and ensure survival under starvation and various stress conditions (the so-called general stress response) [1]. The σ^S^ network has been intensively studied in the model organism, *Escherichia coli* K-12 (*E*. *coli*) [1–3]. In the closely related foodborne pathogen *Salmonella enterica* serovar Typhimurium (*S*. Typhimurium), σ^S^ is required for stress resistance, biofilm formation and virulence [1, 4]. Global transcriptomic and proteomic studies have revealed the composition of the *Salmonella* σ^S^ network, and a major effect of σ^S^ on remodeling of membrane and metabolic functions [5–7]. The observed control by σ^S^ of genes involved in ions trafficking prompted us to assess the impact of σ^S^ on the *Salmonella* ionome. A marked effect of the Δ*rpoS* mutation on the cell-associated concentration of cobalt, manganese, magnesium and potassium was detected by inductively coupled plasma mass spectrometry (ICP-MS) [8], suggesting that a tight control of uptake and availability of these cations might be critical for quiescent bacteria. Consistent with this hypothesis, σ^S^ was required for optimal regrowth of stationary phase cells in rich medium depleted for magnesium [8].

*Salmonella* imports magnesium *via* three known transporters, the widely conserved CorA transporter and the MgtA and MgtB P-type ATPases [9]. CorA is expressed under various growth conditions whereas the other two magnesium transporters, MgtA and MgtB, are expressed under conditions of magnesium starvation under the positive control of the PhoP-PhoQ regulatory system [10]. CorA is regulated at the levels of transcription initiation *via* the stringent response [11] and transcription elongation through an unknown mechanism [12, 13]. CorA can perform magnesium influx and efflux and it is also able to import cobalt when cobalt is present in high concentrations in the growth medium [9, 10, 14–16]. A *Salmonella corA* mutant is affected in virulence in mice, epithelial cell invasion and weakly in macrophage survival [17, 18]. However, the underlying molecular mechanisms are not well understood since, surprisingly, the mutant does not seem to lack magnesium [17, 18].

In our global transcriptomic analysis in LB rich medium, the *mgtA* and *mgtB* genes were expressed to low levels in both the wild-type and Δ*rpoS* strains [5], likely because the magnesium concentration in LB [8] was high enough to prevent detectable expression of these genes [9, 10, 14]. In contrast, the *corA* gene was downregulated in the Δ*rpoS* mutant [5], a finding that was confirmed by using a transcriptional *corA-lacZ* fusion [8]. A reduced expression level of *corA* in the Δ*rpoS* strain may thus contribute to lower the magnesium content of the mutant, compared to the wild-type strain. Nevertheless, potential differences between the wild type strain and the Δ*rpoS* mutant in their membrane composition [5, 7] and/or ribosomes and ATP contents, which represent important reservoirs of magnesium [9, 10, 14, 19, 20], have also to be considered. To address this issue and determine the biological significance of the σ^S^ control of *corA*, we assessed the physiological impact of a Δ*corA* mutation on stationary phase cells by analytical and global analyses. This study unravels a crosstalk between the CorA and PhoP/MgtA systems important for *Salmonella* resilience. Under magnesium proficient environmental conditions, the absence of CorA is sensed by the cell and compensated by PhoP-independent production of MgtA along with PhoP-dependent mechanisms. This regulatory network minimizes the impact of a Δ*corA* mutation on protein content, magnesium homeostasis, growth, and motility of *Salmonella*.

## Material and methods

### Bacterial strains, bacteriophage, plasmids, and growth conditions

Strains and plasmids are listed in S1 Table. Bacteriophage P22HT105/1*int* was used to transfer mutations and *lacZ* fusions between *Salmonella* strains by transduction [21]. Green plates, for screening for P22-infected cells or lysogens, were prepared as described previously [22]. Bacteria were routinely grown in LB medium [23] at 37°C under aeration. When indicated, the LB medium was supplemented with the metal chelating agent ethylenediaminetetraacetic acid (EDTA) and magnesium chloride (MgCl_2_) at the indicated concentrations. Antibiotics were used at the following concentrations (in *μ*g per ml): carbenicillin (Cb), 100; chloramphenicol, (Cm) 15 for the chromosomal resistance gene and 30 for the plasmid resistance gene; kanamycin, (Km) 50; and tetracycline, (Tc) 20.

### DNA manipulations, *lacZ* fusions and inactivation of chromosomal genes

Standard molecular biology techniques were used [23, 24]. Oligonucleotides were obtained from Sigma-Aldrich and are listed in S2 Table. Functional annotations and DNA sequences of ATCC14028 genes were obtained from the KEGG server (www.genome.jp/kegg/kegg2.html) and Uniprot resource (www.uniprot.org). DNA sequencing was performed by Eurofins Genomics (Cologne, Germany). Chromosomal deletions and *lacZ* fusions were generated in *Salmonella* ATCC14028 using PCR-generated linear DNA fragments (S2 Table) and λ-Red recombination-based method [5, 6, 25–27]. All strains were confirmed to contain the expected mutation by DNA sequencing.

### Whole Genome Sequencing (WGS)

The whole genome of the Δ*corA*Δ*mgtA* colony variants (SCV and LCV, S1 Table) and the parental strain ATCC14028s was sequenced at the Novogene Corporation Inc, using an Illumina NovaSeq sequencer. Total genomic DNA was extracted using the FastDNA spin kit for soil (MP Biomedicals). DNA fragmentation, WGS libraries preparation and sequencing was performed by Novogene using a 2 × 150 nucleotides paired-end strategy and the quality of the resulting data was controlled. Library adaptators of paired-ends read were trimmed with Cutadapt version 2.10 (https://doi.org/10.14806/ej.17.1.200) and then mapped to the reference genome of *Salmonella enterica* subsp. *enterica* serovar Typhimurium (GenBank accession numbers CP001362 and CP001363 for the virulence plasmid and chromosome, respectively) using the Burrows-Wheeler Alignment tool, BWA version 0.7.17 with the BWA-mem algorithm. SAMtools version 1.13 and Picard tools version 2.23.3 (http://broadinstitute.github.io/picard) were then used to filter, sort, convert SAM files and assign read groups and mark duplicate reads. Single-nucleotide polymorphism (SNPs) and insertion/deletion (indels) positions were called using Genome Analysis Toolkit version3.1–130 according to the GATK Best Practices for haploid genome (VariantFiltration, QD <2.0, LowQD, ReadPosRankSum<-8.0, LowRankSum, FS >60.0, HightFS, MQRankSum<-12.5, MQRankSum, MQ <40.0, LowMQ, HaplotypeScore >13.0, HaploScore parameters were used to filter SNPs and indels). Twenty-one SNPs and eight INDELs were identified in the genome of the parental strain (VF6910, S1 Table), compared to the published sequence of ATCC14028s [28]. These SNPs and INDELs were also found in the genome of the two Δ*corA*Δ*mgtA* colony variants together with the expected deletions in the *corA* and *mgtA* genes. The Δ*corA*Δ*mgtA* LCV contains two additional SNPs (1277518 C->A and 3612166 G->C), not found in the genome of the Δ*corA*Δ*mgtA* SCV and the wild type strain (see results and discussion section).

### Electrophoresis and immunoblot analysis of proteins

Whole-cell extracts were prepared and SDS-polyacrylamide gel electrophoresis was carried out as described [29, 30]. The amount of proteins in whole-cell lysates was determined using the DC Protein Assay kit (Bio-Rad). Equal amounts of proteins were loaded in each slot. The molecular sizes of the proteins were estimated using Precision Plus Protein Standard (Bio-Rad). Proteins were transferred to nitrocellulose blotting membranes (Amersham Protan, GE Healthcare). Reversible Ponceau staining [23] of the membrane was used to check proteins transfer. For detection of 3x-Flag-tagged proteins, membranes were incubated with a mouse anti-Flag antibody (F3165 Sigma) as previously described [29, 30]. Purified anti-RNA polymerase alpha (663104, Biolegend) and anti-GroEL (G6532 Sigma) antibodies were used to reveal the RNAP-alpha subunit and the GroEL control proteins, respectively. A polyclonal rabbit antibody serum was used to detect the σ^S^ protein of *S*. *enterica* serovar Typhimurium, as previously described [31]. Bound antibodies were detected using secondary anti-mouse (NA9310 Cytiva) and anti-rabbit (NA934 Cytiva) antibodies linked to peroxidase and the Pierce ECL Plus western blotting substrate (Thermoscientific). ImageJ (http://rsb.info.nih.gov/ij/index.html) was used to compare the density of bands.

### Experimental design and statistical analysis of the proteome

#### Bacterial lysis

Bacterial cultures were centrifuged, washed twice with Tris-HCl 100 mM, pH 7.4. Aliquots (1ml at OD600 = 10) were centrifuged and resuspended in 1ml lysis buffer (Urea 8M, Tris-HCl 100 mM, pH7.4). Samples were added to a lysing matrix tube containing specialized lysing matrix particles (tubes with caps Sigma Z763837 and glass beads Sigma G4649) and were lysed at 4°C using the FastPrep-24 5G instrument (3x30sec, pause 180 sec). After centrifugation (15 min, 13200 rpm, 4°C), aliquots (500 μl) were immediately freezed in dry ice and stored at -80°C.

#### Protein digestion

An amount of 50 μg of total protein were solubilized into 80 μL with lysis buffer. Subsequently, samples were reduced in 50 mM TCEP (Sigma ‐ 646547) for 1H followed by alkylation in 50mM iodoacetamide (Sigma ‐ I114) for 1H in the dark. Proteins were first digested with 1 μg rLys-C (Promega ‐ V1671) for 3H at 25°C and a second digestion was performed with 1 μg Sequencing Grade Modified Trypsin (Promega ‐ V5111) for 16H at 37°C. Incubation time and temperature control for reduction, alkylation and digestions were performed with an Eppendorf ThermoMixer® C equipped with a ThermoTop®. The digestion was stop with formic acid (FA) at 1% final and peptides were desalted on reversed phase C18 Sep-Pak Cartridge (Waters ‐ WAT054955). Peptides were eluted 2 times with Acetonitrile (ACN) 50%, FA 0.1% and 1 time with ACN 80%, FA 0.1%. Finally, samples were dried in vacuum centrifuge and resuspended with ACN 2% / FA 0.1%.

#### LC-MS/MS analysis

A nanochromatographic system (Proxeon EASY-nLC 1200 ‐ Thermo Fisher Scientific) was coupled on-line to a Q Exactive^TM^ Plus Mass Spectrometer (Thermo Fisher Scientific) using an integrated column oven (PRSO-V1 ‐ Sonation). For each sample, peptides were loaded on an *home-made* 38 cm capillary column picotip silica emitter tip (75 μm inner diameter) with C18 resin (1.9 μm particles, 100 Å pore size, Reprosil-Pur Basic C18-HD resin, Dr. Maisch) after an equilibration step in 100% solvent A (H2O, FA 0.1%). Peptides were eluted with a multi-step gradient using 2 to 7% solvent B (ACN 80%, FA 0.1%) during 5 min, 7 to 23% during 120 min, 23 to 45% during 20 min and 45 to 95% during 7 min at a flow rate of 250 nL/min over 167 min. Column temperature was set to 60°C. MS data were acquired using Xcalibur software using a data-dependent Top 10 method with a survey scans (300–1700 m/z) at a resolution of 70,000 and a MS/MS scans (fixed first mass 100 m/z) at a resolution of 17,500. The AGC target and maximum injection time for the survey scans and the MS/MS scans were set to 3E6, 50 ms and 1E6, 60 ms respectively. The isolation window was set to 1.6 m/z and normalized collision energy fixed to 28 for HCD fragmentation. We used a minimum AGC target of 1E4 for an intensity threshold of 1.7E5. Unassigned precursor ion charge states as well as 1, 7, 8 and >8 charged states were rejected and peptide match was disable. Exclude isotopes was enabled and selected ions were dynamically excluded for 45 seconds.

#### Data analysis

*MaxQuant*. Raw data were analyzed using MaxQuant software version 1.6.6.0 [32] using the Andromeda search engine [33]. The MS/MS spectra were searched against the *Salmonella* typhimurium strain 14028s UniProt database (5,369 entries the 17/01/2021) and against a small protein database containing 5 proteins. Andromeda searches were performed choosing trypsin as specific enzyme with a maximum number of two missed cleavages. Possible modifications included carbamidomethylation (Cys, fixed), oxidation (Met, variable) and Nter acetylation (variable). The mass tolerance in MS was set to 20 ppm for the first search then 4.5 ppm for the main search. The mass tolerance in MS/MS was set to 20 ppm. Maximum peptide charge was set to seven and seven amino acids were required as minimum peptide length. The “match between runs” feature was applied for samples having the same experimental condition with a maximal retention time window of 0.7 minute. One unique peptide to the protein group was required for the protein identification. A false discovery rate (FDR) cutoff of 1% was applied at the peptide and protein levels. Quantification was performed using the XIC-based LFQ algorithm with the Fast LFQ mode as described in Ref. [34]. Unique and razor peptides included modified peptides, with at least 2 ratio count were accepted for quantification.

*PEAKS studio*. Data were analysed using PEAKS Studio 7.0 [35]. *De novo* sequencing and database search of peptides was performed with common parameter settings such as trypsin digestion, parent mass tolerance 5 ppm and fragment mass tolerance 0.02 Da. Possible modifications included carbamidomethylation (Cys, fixed), oxidation (Met, variable) and Nter acetylation (variable). For *de novo* sequencing ALC score was set ≥50% and for database search the same database as MaxQuant was used. A cutoff of 1% false discovery rate (FDR) was applied at PSM level.

#### Statistical analysis

To find the proteins more abundant in one condition than in another, the LFQ intensity values from Maxquant were compared. Reverse hits, potential contaminants, and proteins “Only identified by site” were removed from the analysis. Then proteins identified with at least one peptide that is not common to other proteins in the FASTA file used for the identification (at least one "unique" peptide) were kept. Additionally, only proteins with at least four LFQ intensity values in one of the two compared conditions were kept for further statistics. Proteins absent in a condition and present in another are put aside. These proteins can directly be assumed differentially abundant between the conditions. After this filtering, intensities of the remaining proteins were first log-transformed (log2). Next, intensity values were normalized by median centering within conditions (section 3.5 in [36]). Missing values were imputed using the impute.mle function of the R package imp4p [37]. Statistical testing was conducted using a limma t-test thanks to the R package limma [38]. An adaptive Benjamini-Hochberg procedure was applied on the resulting p-values thanks to the function adjust.p of the cp4p R package [39] using the robust method described in [40] to estimate the proportion of true null hypotheses among the set of statistical tests. The proteins associated to an adjusted p-value inferior to an FDR (false discovery rate) level of 1% and an absolute log2(fold-change) superior to 1 have been considered as significantly differentially abundant proteins. Finally, the proteins of interest are therefore those which emerge from this statistical analysis supplemented by those which are present from one condition and absent in another (“ID Table” in the S1 Dataset).

### Functional analysis

GO terms associated to each identified protein were retrieved from Uniprot database (www.uniprot.org) and hypergeometric tests were performed to test the overrepresentation hypothesis for each GO term using R software. All the proteins identified in our mass spectrometry experiments have been used as background for the hypergeometric tests. A significantly low p value means the proportion of proteins related to a GO-term is significantly superior in the considered list than in this background (S15 Fig). STRING database (http://string.embl.de/) was used to visualize protein-protein interactions. The active interaction sources were Text mining, Experiments, Database, Co-expression, Neighborhood, Gene fusion, and Co-occurrence, with a required minimum combined score of major confidence (0.9).

### Accession numbers

The mass spectrometry proteomics data have been deposited to the ProteomeXchange Consortium via the PRIDE [41] partner repository with the dataset identifier PXD041489. The genome sequence data have been deposited to the NCBI SRA database with the accession number PRJNA931917.

### Assay for the cell-associated magnesium content

The cell-associated magnesium content was measured using the Magnesium assay kit (MAK026, Sigma) according to the manufacturer’s recommendations. Results were expressed in nmoles of magnesium in whole-cell lysates, relative to the amount of total proteins determined using the DC Protein Assay kit (Bio-Rad).

### Assay for the intracellular free magnesium content

A method using the Mg^2+^- sensitive fluorescent dye Mag-Fura2 has previously been developed for dual-excitation ratio-metric determination of free intracellular magnesium content of *S*. Typhimurium [42]. This method was used in this study to compare the amount of intracellular free magnesium of the wild-type *Salmonella* strain ATCC14028s and its mutant derivatives. Since the probe has to be cleaved by intracellular esterases to be able to interact with magnesium, the membrane permeant Pluronic F-127 was used to allow entry of the probe into the cell [42]. Mag-Fura2 shows a different fluorescence spectrum depending on whether it is bound to magnesium, and the ratio between the fluorescence intensities at 340 nm and 380 nm reflects the abundance of free intracellular magnesium. The fluorescence ratio per OD_600_ of the bacterial culture was estimated for each strain tested, using three biological replicates. Relative fluorescence values (fluorescence ratio per OD_600_ for a mutant relative to the fluorescence ratio per OD_600_ for the reference strain measured within the same experiment) were used for comparison between strains through independent experiments.

### Competition assays

Overnight LB cultures were washed and resuspended in phosphate- buffered saline (NaCl 137 mM, KCl 2.7 mM, Na2HPO4 10 mM, KH2PO4 1.76 mM) to an OD_600_ of 1.0. Equal numbers of cells of the wild-type strain ATCC14028 and the mutant strain were then mixed in fresh LB medium to give a total of about 3000 cells ml^-1^ and the mixture was incubated at 37°C with shaking. In some experiments, the LB medium was supplemented with MgCl_2_ at the indicated concentrations. Aliquots of bacteria were removed at timed intervals and numbers of viable cells of each strain were determined on LB plates containing the appropriate antibiotics. P values were calculated using a two-tailed t test.

### Motility assays

1 *μ*l of stationary phase LB culture was inoculated into 0.3% agar LB plates that were incubated at 37°C for 5 h. In some experiments the LB medium was supplemented with MgCl_2_ at the indicated concentrations.

### Enzymatic assays

β-galactosidase activity was measured as described by Miller [43] and is expressed in Miller units.

### Statistical analysis

Student’s t-test was performed for pairwise comparisons. Values were presented as means ± standard error of the mean (SEM). Differences were considered significant when p ≤ 0.05.

## Results and discussion

### Mg^2+^-transporters in *Salmonella*

**Fig 1:**
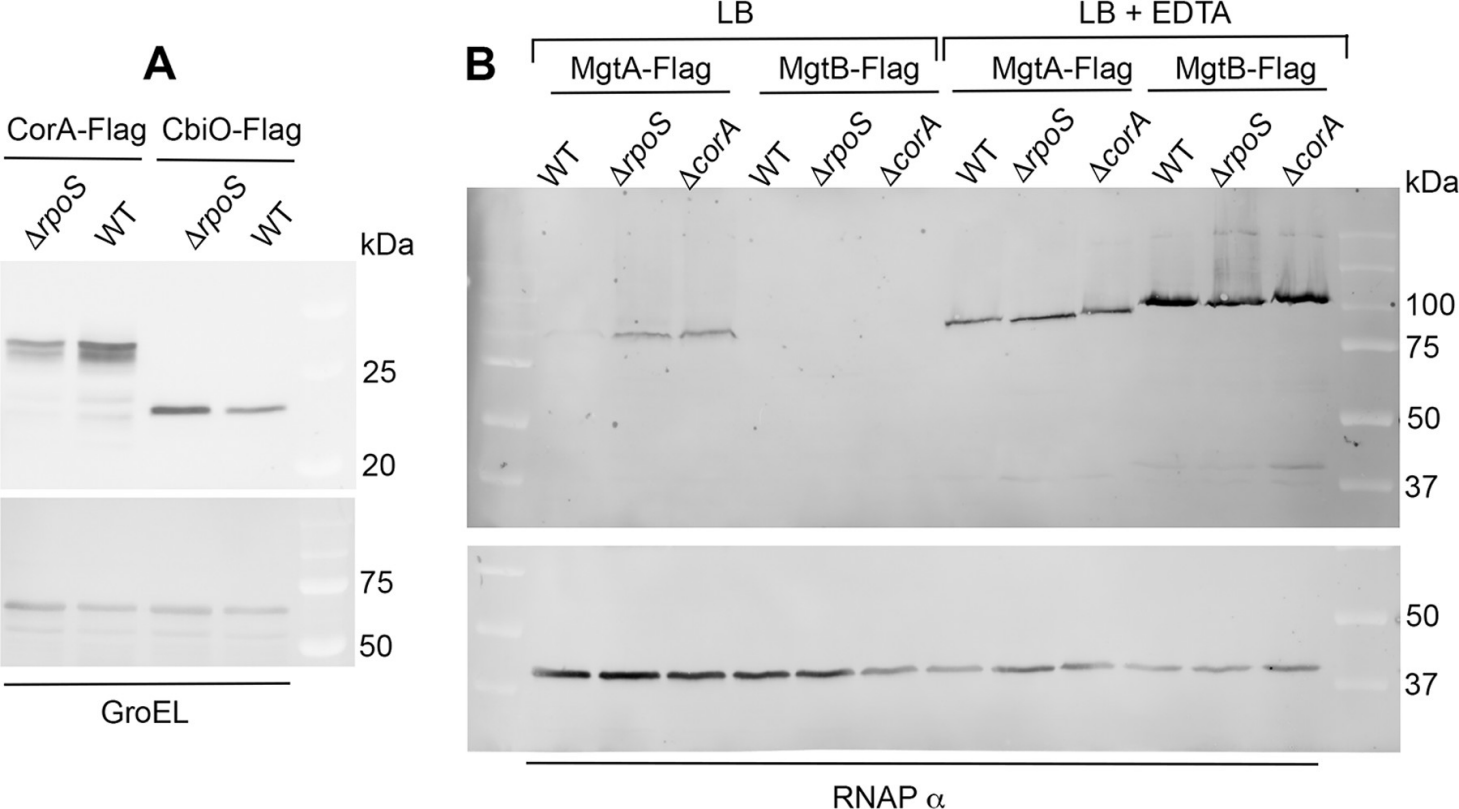
Magnesium transporters produced in *Salmonella*. (A) The CorA-Flag protein was immunodetected in *Salmonella* wild-type and Δ*rpoS* strains grown for 18 h in LB at 37°C. The Flag-tagged CbiO protein produced from the *cbiMNQO* operon encoding a high affinity cobalt uptake system [48] negatively regulated by σ^S^ [7, 8] was used as a control. Membranes used to reveal the Flag-tagged proteins with the anti-Flag antibody were then incubated in the presence of antibodies directed against GroEL used as a loading control of total protein amounts. Relative quantification of bands intensity (normalized to GroEL) indicated that the Δ*rpoS* mutation decreased by about 3.5-fold the amounts of CorA. The amount of the CbiO-Flag control protein was increased by about 1.5-fold, in agreement with our previous finding that expression of a translational *cbiO-lacZ* fusion is increased by about 2-fold by the Δ*rpoS* mutation [8]. (B) The MgtA-Flag and MgtB-Flag proteins were immunodetected in the wild-type strain and the Δ*rpoS* and Δ*corA* mutants grown for 18h at 37°C in LB supplemented or not with EDTA 2 mM. Membranes used to reveal the Flag-tagged proteins with the anti-Flag antibody were then incubated in the presence of antibodies directed against the alpha subunit of RNA polymerase used as a loading control of total protein amounts. A representative experiment is shown. Similar results were found in repeat experiments (Figs S1 and S7 and 4).

Three magnesium transporters, CorA (37-kDa), MgtA (95-kDa) and MgtB (101 kDa) have been characterized in *Salmonella* [9]. In previous RNA sequencing experiments using wild-type and Δ*rpoS* strains of *S*. Typhimurium ATCC14028 grown to stationary phase in LB rich medium, *corA* mRNA was detected in lower amounts in the Δ*rpoS* mutant than in the wild-type strain [5]. In the same growth conditions, the *mgtA* and *mgtB* mRNAs were detected to low levels in both the wild-type and Δ*rpoS* strains [5]. The positive effect of σ^S^ on *corA* expression was confirmed using a *corA-lacZ* transcriptional fusion [8] and a *corA*-flag construct (Fig 1A). Magnesium concentrations in LB (100 to 200 μM, [8] and unpublished data) were likely too high to allow detectable expression of *mgtA* and *mgtB* [10]. Consistent with this hypothesis, the corresponding flag-tagged proteins were not immunodetected (MgtB) or immunodetected to very low levels (MgtA) in wild-type *Salmonella* grown to stationary phase in LB, except when the LB was supplemented with EDTA which chelates cations such as Mg^2+^ (Figs 1B and S1A). Similarly, the PhoPQ-regulated MgtC protein was produced from the *mgtCB* operon only when LB was supplemented with EDTA (S1B Fig). These findings are consistent with previous studies indicating that, whereas PhoP is active in LB broth [44], cytoplasmic Mg^2+^ levels are too high to allow significant *mgtA* and *mgtB* expression [45–47]. In conclusion, CorA was likely the main magnesium transporter produced in *Salmonella* grown to stationary phase in LB, and its expression was favored by σ^S^.

### The Δ*corA* mutant has a competitive disadvantage to exit from stationary phase

To determine whether activation of *corA* expression by σ^S^ confers advantages to stationary phase bacteria, we first assessed the effect of a Δ*corA* mutation on σ^S^-dependent phenotypes. Motility of *Salmonella* is impaired by both the Δ*rpoS* ([5], S2A Fig) and Δ*corA* mutations (S2A Fig and [17]) opening the possibility that the Δ*rpoS* mutation affects motility by altering *corA* expression. However, the Δ*corA* mutation decreased the motility of both the wild-type strain and the Δ*rpoS* mutant (S2A and S2B Fig), suggesting additive effects of the mutations. The Δ*corA* mutation was complemented by the plasmid-borne *corA* gene (S2C Fig). σ^S^ is required for stationary phase survival in LB [29]. However, the Δ*corA* mutation had no significant effect on stationary phase survival of the wild strain and the Δ*rpoS* mutant (S3A Fig).

**Fig 2:**
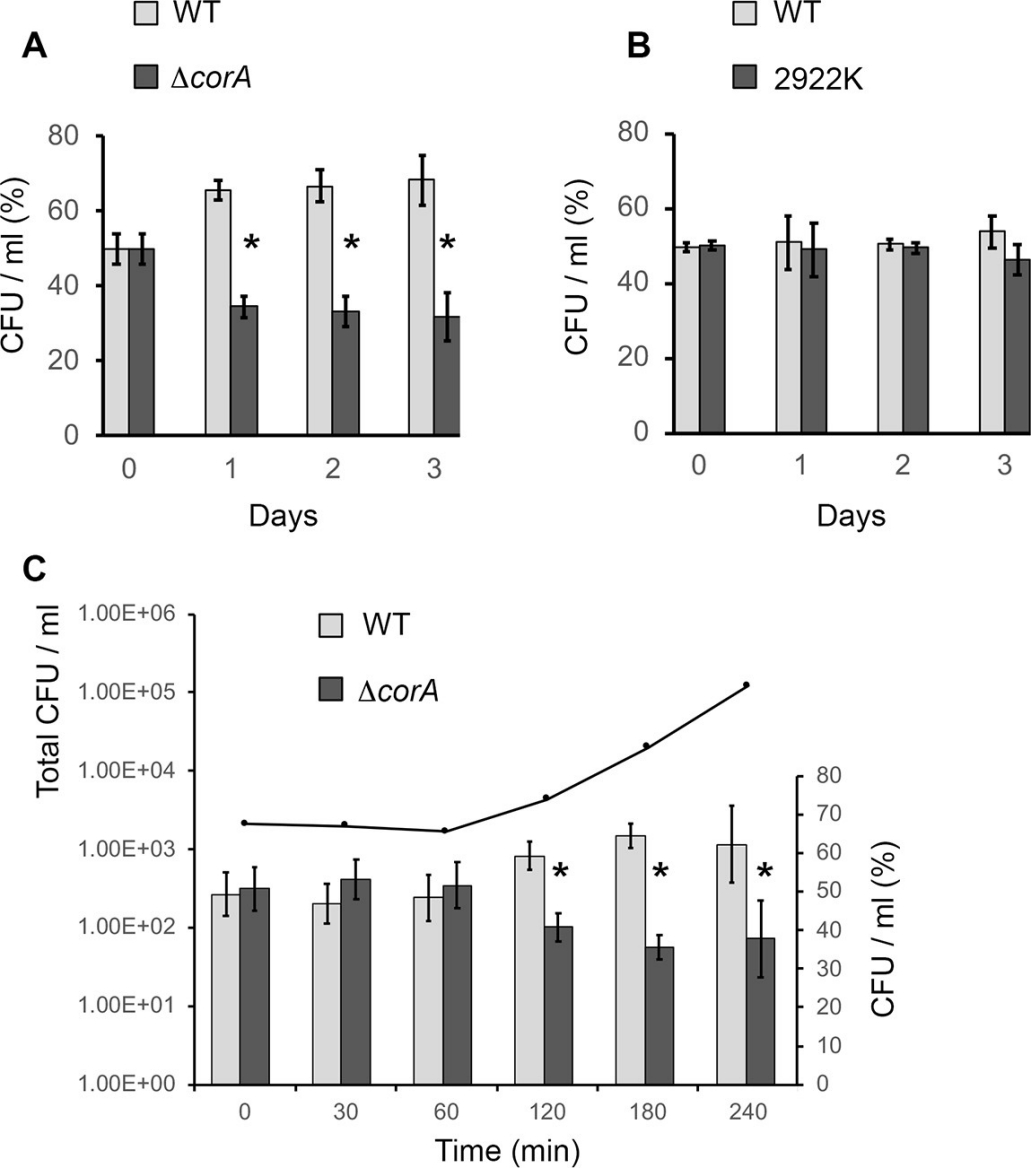
Competition experiments between the wild-type strain and the Δ*corA* mutant of *Salmonella*. Competition assays were performed between the wild-type strain ATCC14028 (WT) and the Δ*corA* mutant (panel A) or the control strain ATCC14028 2922K (panel B) which carries the same kanamycin resistance cartridge as the Δ*corA* mutant. The control competition assay showing similar fitness of ATCC14028 and 2922K is consistent with our previous data [5, 29]. Equal cell numbers of stationary phase LB cultures of the wild-type strain and the mutant strain were mixed in fresh LB medium to give a total of about 3000 cells ml-1 (Day 0) and the mixtures were incubated at 37°C with shaking. Aliquots of bacteria were removed at timed intervals and numbers of viable cells of each strain were determined. For each time point, cells number of each strain is reported as a percentage of the total number of viable cells in the culture. The error bars represent standard errors for three independent measurements. At day 0, the Δ*corA* mutant represented about 50% of a total population of 3000 CFU/ml. At day 1, the Δ*corA* mutant represented about 34% of a total population of 5 10^9^ CFU/ml, which corresponds to 1.7 10^9^ Δ*corA* cells and 3.3 10^9^ wild-type cells. * Statistically significant competitive disadvantage of the mutant compared to the wild-type (p-value <0.01). (C) In this experiment, competition assays between the wild-type strain and the Δ*corA* mutant were monitored immediately after inoculation of the bacterial mixture into fresh LB medium. The black curve represents the growth of the total bacterial population estimated by CFU counts (left y axis) at regular intervals after inoculation of the bacterial mixture into fresh LB. Bars represent the percentage of each strain within the population (right y axis) at the different time points. The error bars represent standard errors for three independent measurements. * p-value <0.01.

Since our recent results suggest that σ^S^ plays a role in the dynamics of exit of *Salmonella* from stationary phase [8], the regrowth potential of the Δ*corA* mutant was also determined. Whereas growth of the Δ*corA* mutant in LB was not significantly different from that of the wild-type strain in monocultures (S3B and S3C Fig), the Δ*corA* mutant was disadvantaged in competition experiments with the wild-type strain (Fig 2A). The competitive disadvantage of the Δ*corA* mutant was similar at day 1, day 2 and day 3, indicating that the Δ*corA* mutation did not impact *Salmonella* stationary phase survival in both competition experiments (Fig 2A) and monocultures (S3A Fig). When the proportion of the wild-type and Δ*corA* mutant in the total population were measured at regular intervals after inoculation into fresh LB, the cost of the Δ*corA* mutation was the most apparent at the end of the lag phase (Fig 2C). Indeed, the proportion of Δ*corA* mutant within the total population decreased significantly from 120 minutes post-inoculation, corresponding to the exit from the lag phase, which suggests that the Δ*corA* mutant had a competitive disadvantage to exit from stationary phase.

### The Δ*corA* mutation does not affect the total and free magnesium content of cells

**Fig 3:**
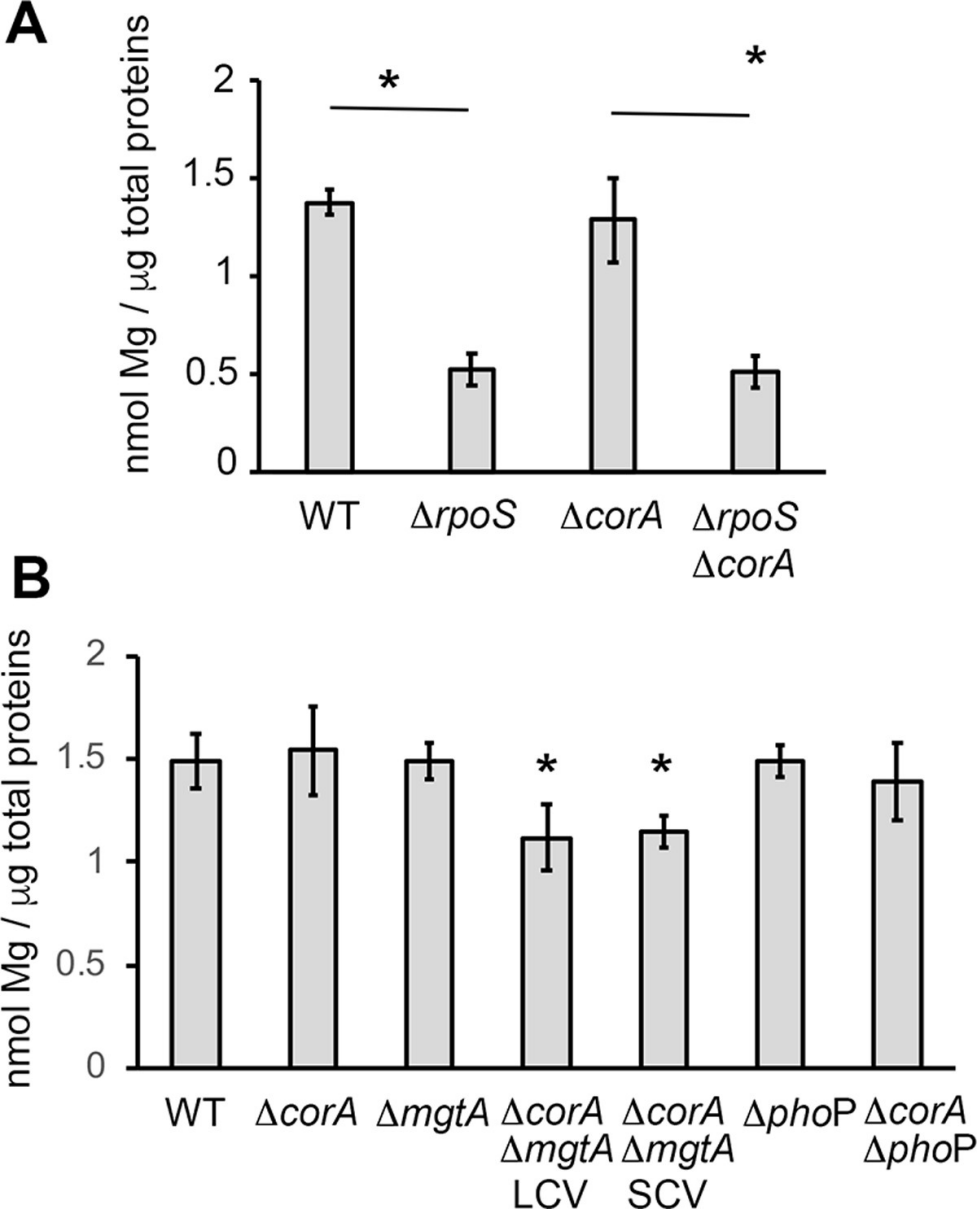
Total magnesium content of *Salmonella* wild-type and mutant strains. The *Salmonella* wild-type and mutant strains were grown for 18 h at 37°C in LB (A) or in LB supplemented with MgCl_2_ 10 mM (B) and cell-associated magnesium content was measured as described in Material and Methods. Bars represent the mean of at least three independent measurements and the error bars represent the standard error. * p-value <0.01.

Using both ICP-MS [8] and a commercialized biochemical assay (Fig 3), we showed that the cell-associated magnesium content in stationary phase LB cultures of *Salmonella*, was lowered by the Δ*rpoS* mutation. Decreased level of cell-associated Mg^2+^ in the Δ*rpoS* mutant might be caused by the decreased level of CorA, and the competitive disadvantage of the Δ*corA* mutant in exiting stationary phase might be due to a decrease in magnesium levels in the stationary phase cells. However, under the same growth conditions, the Δ*corA* mutation had no significant effect on the cell-associated magnesium amount of the wild-type strain and the Δ*rpoS* mutant (Fig 3A). This result is surprising, considering that CorA is the primary magnesium channel in *Salmonella*, but it is consistent with previous studies from Papp-Wallace and Maguire [18]. This finding suggests that 1) The impact of the Δ*rpoS* mutation on magnesium content does not result from a defect in *corA* expression; 2) other systems allow the import of magnesium into the Δ*corA* mutant and 3) the competitive disadvantage of the Δ*corA* mutant to exit from stationary phase does not result from a variation in the total magnesium content of the mutant at the time of inoculation.

Lag phase is a poorly understood stage of the bacterial growth cycle [49–51]. This period prepares bacteria for the replicative phase and is thus critical for competitive growth of bacteria and possibly antibiotic tolerance. To address a potential effect of the Δ*corA* mutation on magnesium trafficking during the lag phase, the amount of magnesium associated with wild-type and Δ*corA* cells was measured at different intervals during the lag phase preceding the resumption of growth and until the entry to stationary phase (S4 Fig). No significant difference was observed between the wild-type strain and the Δ*corA* mutant. During the lag phase, the number of viable cells was constant (S4B Fig) whereas the OD_600_ slightly increased (S4A Fig), indicating an increase in cell size. During this period, the total magnesium level, relative to cellular proteins, decreased (S4C Fig), suggesting that magnesium is not consumed during the lag phase. This conclusion is consistent with a previous study showing that, although the lag phase of growth of *Salmonella* in LB involves transient metal accumulation, the cell-associated concentration of magnesium does not change significantly during this period [49]. To further assess consumption (or efflux) of magnesium during the lag phase, the magnesium concentration in the extracellular medium was measured during growth (S5 Fig). The extracellular magnesium concentration was stable during the lag phase and decreased during exponential and early stationary phase of *Salmonella* growth (S5C Fig), confirming that *Salmonella* does not consume magnesium during the lag phase. Similar results were obtained for the wild-type strain and the Δ*corA* mutant (S5 Fig).

Data in Figs 3 and S4 and [18] demonstrated that the total magnesium content of cells is not modified by the Δ*corA* mutation. However, since most (98%) of magnesium molecules are bound to nucleotide triphosphates (mainly ATP), ribosome, DNA, proteins and membrane [9, 10, 52], a possible effect of the Δ*corA* mutation on the free magnesium level of cells might have been overlooked. We thus applied a published method, using the fluorescent probe Mag-Fura2 [42], to compare the intracellular free magnesium contents of the wild-type strain and the Δ*corA* mutant. Since the Δ*rpoS* mutant showed a reduced content of total magnesium, compared to the wild-type strain [8] (Fig 3), we used this strain as a control. The amount of free magnesium was slightly reduced in the Δ*rpoS* mutant compared to the wild-type strain (S6A Fig). Under similar growth conditions, however, the Δ*corA* mutation did not modify the amount of free magnesium of the wild-type strain (S6B Fig) and the Δ*rpoS* mutant (S6C Fig). Thus, the Δ*corA* phenotypes are not due to variations in the free magnesium level of the mutant.

Altogether these data are consistent with the conclusion that the Δ*corA* strain does not lack magnesium and that other systems are involved in magnesium import in the Δ*corA* mutant.

### Increased MgtA production in the Δ*corA* mutant

**Fig 4:**
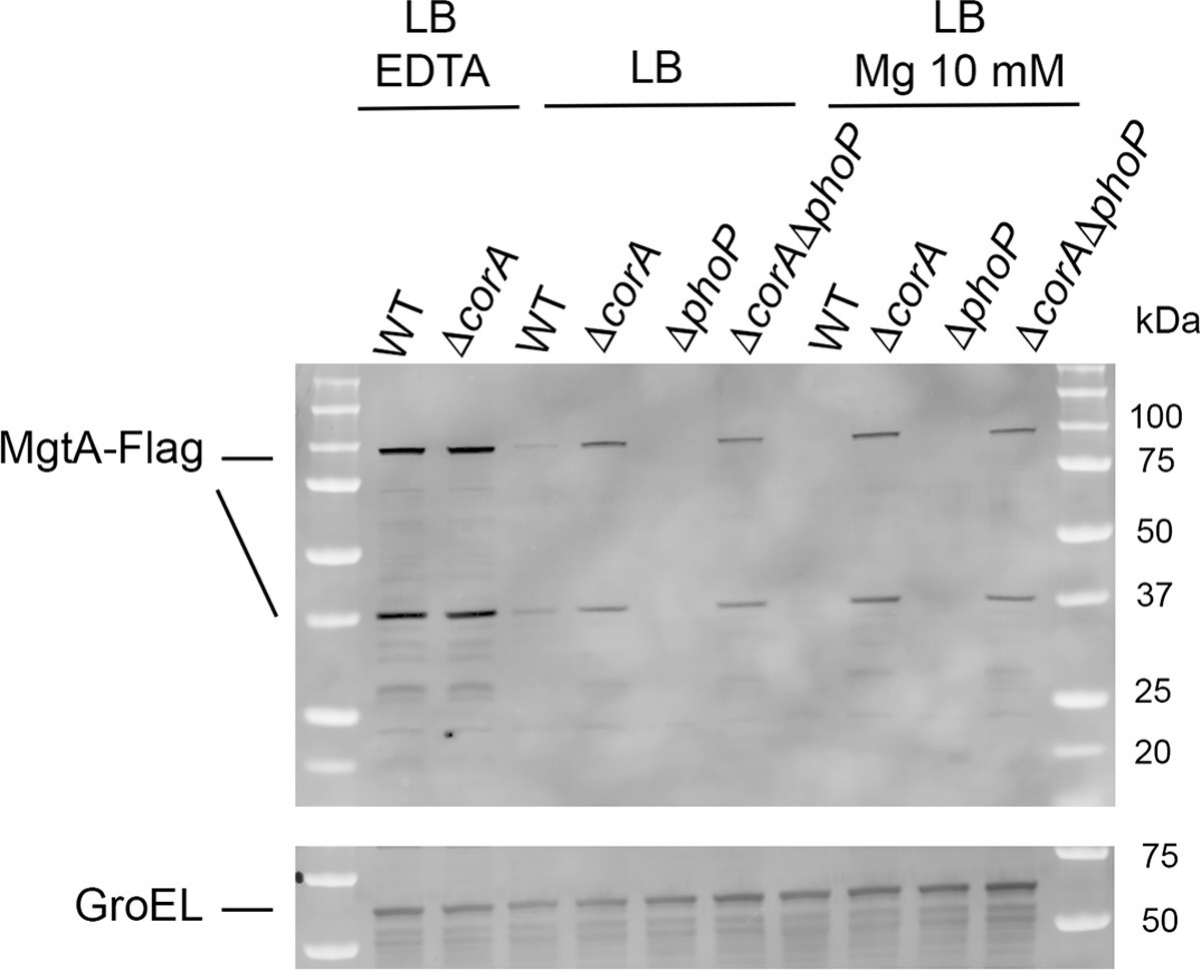
Immunodetection of MgtA in *Salmonella* wild-type and mutant strains. The MgtA-Flag protein was immunodetected in *Salmonella* wild-type and mutant strains grown for 18 h at 37°C in LB supplemented or not with MgCl_2_ 10 mM or EDTA 2 mM. MgtA is a 95-kDa protein. In most immunodetection experiments, two MgtA-Flag products were found (a full-length product at about 98-kDa and a smaller product of about 38-kDa), suggesting that the MgtA-flag protein was partially degraded. Membranes used to reveal the Flag-tagged proteins with the anti-Flag antibody were then incubated in the presence of antibodies directed against GroEL used as a loading control of total protein amounts. A representative experiment is shown. Similar results were found in repeat experiments (S7 Fig).

Besides CorA, MgtA and MgtB are the only magnesium transporters characterized so far in *Salmonella* [9, 10]. Interestingly, in stationary phase LB cultures, the MgtA-flag protein was immunodetected in the Δ*corA* mutant and in the Δ*rpoS* mutant, but not in the wild-type strain (Fig 1B). This effect of the Δ*corA* and Δ*rpoS* mutations appeared specific for MgtA since the MgtB-flag and MgtC-flag proteins were immunodetected only when LB was supplemented with EDTA (Figs 1B and S1), in agreement with induction of their production upon magnesium starvation [10]. Low levels of cytoplasmic Mg^2+^ activate elongation of *mgtA* through a magnesium-sensing mRNA leader [10, 45–47, 53]. In the Δ*rpoS* mutant, the decreased amount of Mg^2+^ (Figs 3 and S6) and the decreased amount of CorA (Fig 1) may promote *mgtA* expression. Similar levels of MgtA were detected in the Δ*corA* strain grown in LB and in LB supplemented with MgCl_2_ 10 mM, indicating that MgtA production in the Δ*corA* mutant is not repressed by high external concentrations of magnesium, in contrast to the situation in the wild-type strain (Figs 4 and S7A). Alternatively, MgtA levels were not higher in the Δ*corA* mutant than in the wild-type strain when the growth medium was supplemented with EDTA (Figs 1B and 4 and S7B), and thus when *mgtA* was likely fully induced.

### PhoP is not required for MgtA production in the Δ*corA* mutant

MgtA production is induced by PhoP under magnesium starvation [10]. However, since MgtA production in the Δ*corA* mutant was not repressed by high extracellular magnesium concentration (Figs 4 and S7), we wondered whether PhoP was required. To address this issue, MgtA-flag was immunodetected in strains harboring the Δ*corA* and Δ*phoP* mutations alone and in combination. MgtA production was very low in the wild-type strain grown in LB (Figs 1B and 4 and S7) and undetectable in the Δ*phoP* mutant (Figs 4 and S7), as expected [10]. The Δ*corA* mutation improved MgtA production in both the wild-type strain and the Δ*phoP* mutant, indicating that PhoP was not required. As noticed previously, when LB was supplemented with EDTA, MgtA amounts were high and similar in the wild-type strain and the Δ*corA* mutant (Figs 1B and 4). The Δ*phoP* and Δ*corA*Δ*phoP* mutants did not grow (or grew poorly) in the presence of EDTA, in agreement with the major role of PhoP under conditions of magnesium starvation [10].

### The Δ*corA* mutation strongly impairs *Salmonella* growth in the absence of *phoP*

**Fig 5:**
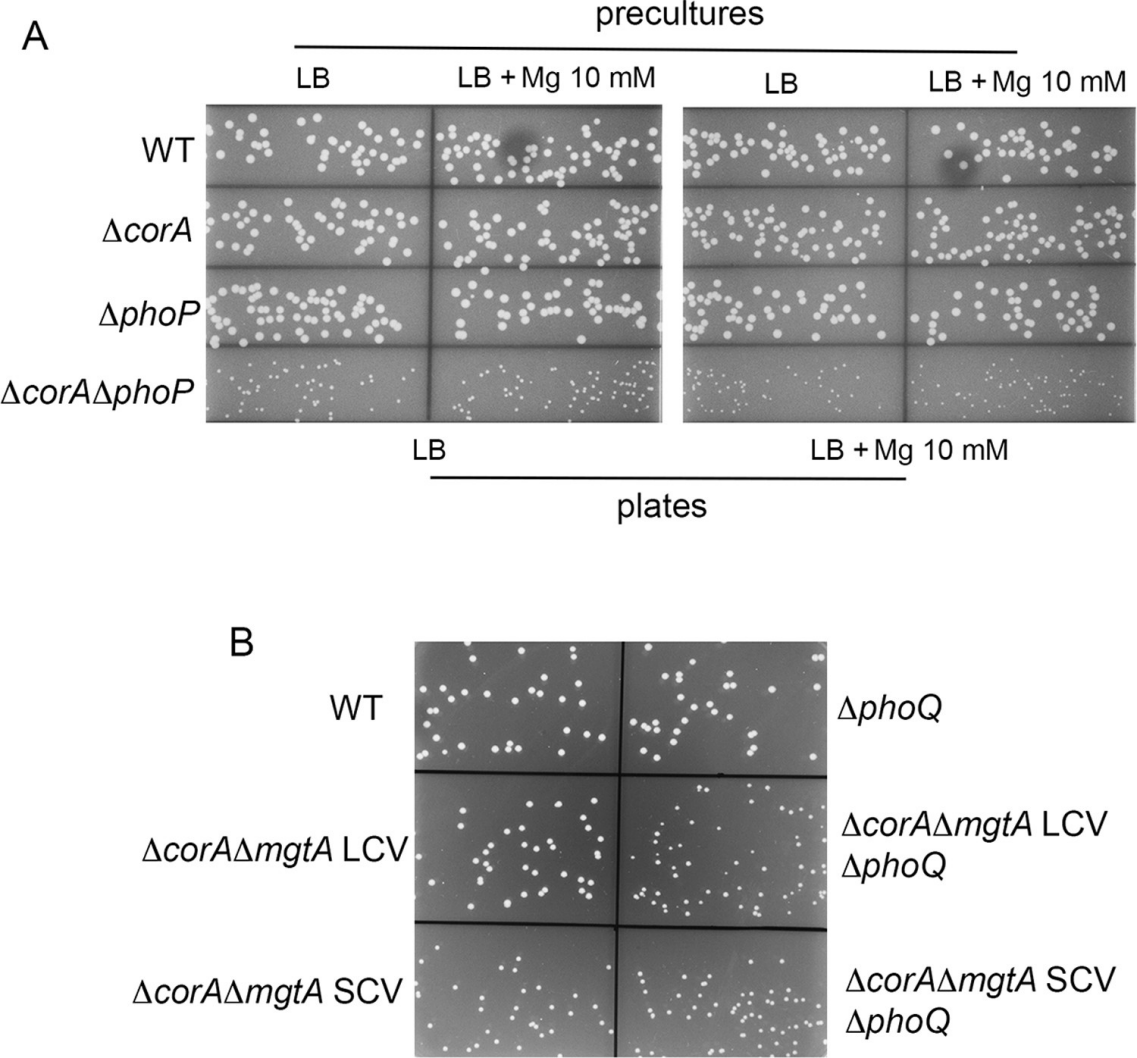
The Δ*corA* mutation impairs *Salmonella* growth in the absence of PhoP or MgtA. (A) The Δ*corA* mutation impairs *Salmonella* growth in the absence of *phoP*. The *Salmonella* wild-type, Δ*corA*, Δ*phoP* and Δ*corA*Δ*phoP* strains were grown 18 h in LB and in LB supplemented with MgCl_2_ 10 mM at 37°C. Cultures were spread on LB plates supplemented or not with MgCl_2_ 10 mM which were incubated at 37°C and colony size was examined overnight. (B) The phoQ*_R16S_ allele improves growth of the Δ*corA*Δ*mgtA* mutant. The *Salmonella* wild-type, Δ*corA*, and Δ*corA*Δ*mgtA* large and small colony variants (LCV and SCV, respectively) carrying or not a Δ*phoQ* mutation were grown 18 h in LB at 37°C. Cultures were spread on LB plates which were incubated at 37°C and colony size was examined overnight. Compared to the wild-type strain, growth of the Δ*corA*Δ*mgtA* SCV was affected (B), but to a lesser extent than that of the Δ*corA*Δ*phoP* strain (A). The Δ*corA*Δ*mgtA* LCV carrying the mutated *phoQ* allele (phoQ*_R16S_, see text) did not show visible growth defect compared to the wild-type strain.

PhoP was not required for MgtA production in the Δ*corA* mutant (Figs 4 and S7). However, we observed a synthetic growth phenotype when the Δ*corA* and Δ*phoP* mutations were combined (Fig 5A). Colonies of the Δ*corA*Δ*phoP* mutant were smaller than that of the wild-type strain and the Δ*corA* and Δ*phoP* mutants (Fig 5A), except when the plasmid-borne *corA* gene was introduced *in trans* (S8 Fig). The PhoP-PhoQ system responds to extracellular low Mg^2+^ and is important for fitness under low Mg^2+^ stress [10]. We thus wondered whether the growth defect of the Δ*corA*Δ*phoP* mutant might be alleviated by high extracellular magnesium concentrations. However, supplementation of LB plates with magnesium 10 mM did not improve growth of the Δ*corA*Δ*phoP* (Fig 5A). The Δ*corA*Δ*phoP* strain was also affected during growth in liquid LB whatever the concentration of magnesium (S9 Fig). Consistently, the competitive growth disadvantage of the Δ*corA* mutant (Fig 2A) was aggravated in the absence of *phoP* (S10BD Fig). Since PhoP was required for normal growth of the Δ*corA* strain even when the extracellular magnesium concentration is high (Figs 5 and S9), PhoP was likely activated by another signal than magnesium starvation. Interestingly, the Δ*phoP* mutant had a growth advantage in competition with the wild-type strain (S10AC Fig), but not in monocultures (S9 Fig). This finding suggests that, under these conditions, PhoP activation confers a fitness cost when CorA is present. The Δ*phoP* mutant was not able to rescue the Δ*corA*Δ*phoP* mutant in competition experiments (S11 Fig).

Unexpectedly, supplementation of LB medium with 10 mM magnesium aggravated the competitive growth disadvantage of the Δ*corA* mutant (S12 Fig). A growth defect of the Δ*corA* mutant in liquid LB containing magnesium 10 mM was detectable in some experiments when monocultures of the wild-type and Δ*corA* strains were compared, but results were not reproducible (not shown). The colony sizes and the cellular magnesium contents of the wild-type and Δ*corA* strains were similar whatever LB was supplemented or not with magnesium (Figs 5 and 3).The wild-type strain might compete with the Δ*corA* strain to exit from stationary phase by limiting access of the Δ*corA* strain to a molecule favoring growth or by increasing the concentration of a component limiting growth of the mutant, and this competition is exacerbated when magnesium level is high.

### MgtA, but not PhoP, contributes to magnesium homeostasis in the Δ*corA* mutant

Results above indicated that, in the absence of CorA, PhoP is required for growth, but not for MgtA production. To assess the physiological impact of PhoP-independent production of MgtA in the Δ*corA* mutant, we constructed Δ*corA*Δ*mgtA* strains. However, during this construction, small and large colonies were recovered, suggesting the occurrence of secondary mutation(s) when the two mutations are combined. One large and one small Δ*corA*Δ*mgtA* colony variants were selected (SCV and LCV). On LB plates (Fig 5B) and in liquid LB (S13 Fig), growth of the Δ*corA*Δ*mgtA* SCV was retarded, but to a lesser extent than that of the Δ*corA*Δ*phoP* mutant. Magnesium supplementation did not improve growth of the Δ*corA*Δ*mgtA* SCV strain and even had a negative effect (compare growth of the Δ*corA*Δ*mgtA* SCV on S13 and S14A Figs). In competition experiments also (S14B Fig), magnesium supplementation did not rescue the Δ*corA*Δ*mgtA* mutants. The magnesium amounts of the two Δ*corA*Δ*mgtA* variants were similar and slightly lower than that of the wild-type strain and Δ*mgtA* mutant (Fig 3B). The Δ*phoP* mutation had no effect on magnesium content of the wild-type and Δ*corA* strains (Fig 3B), which is consistent with our conclusion that PhoP is not required for MgtA production in the Δ*corA* strain (Fig 4). Thus, under these conditions, MgtA, but not PhoP, is involved in magnesium homeostasis in the absence of CorA.

The Δ*mgtA* mutation did not affect *Salmonella* growth (S13 and S15A Figs). To determine why the two Δ*corA*Δ*mgtA* constructs had a different growth phenotype (Figs 5B and S13), their genome sequence was determined to identify potential secondary mutation(s). Except for the expected Δ*corA* and Δ*mgtA* deletions, the genome sequence of the Δ*corA*Δ*mgtA* SCV was identical to that of the parental strain ATCC14028. Thus, the Δ*corA* mutation impairs *Salmonella* growth in the absence of MgtA (Figs 5B and S13). Of note, growth of the Δ*corA* mutant carrying the *mgtA*-flag construct was not impaired (S15B Fig), indicating that the flag-tagged MgtA protein was functional.

As previously observed for the Δ*phoP* mutation, the Δ*phoQ* mutation did not impair *Salmonella* growth except when the Δ*corA* gene was deleted (S16 Fig). However, the colony size of the Δ*corA*Δ*mgtA* LCV was reduced and was like that of the Δ*corA*Δ*mgtA* SCV when the *phoQ* allele was deleted (Fig 5B). Compared to the Δ*corA*Δ*mgtA* SCV, the Δ*corA*Δ*mgtA* LCV contains two SNPs. One SNP is in STM14_4149 (*tuf*_1 gene, 3612166 G->C) and it modifies the penultimate codon of *tuf_1* (GAG instead of CAG) without modifying the amino acid sequence of the encoded elongation factor Tu_1 (leucine in both cases). The other SNP is in the *phoQ* gene (phoQ*_R16S_ allele, at pos 1277518 C->A). This SNP modifies the 16^th^ codon of *phoQ* (AGT instead of CGT), yielding to an Arg/Ser amino acid substitution in the cytoplasmic N-terminal portion of PhoQ. Altogether these data indicate that 1) the Δ*mgtA* mutation impairs both the growth and magnesium content of the Δ*corA* strain and 2) the phoQ*_R16S_ is a gain of function mutation required for suppressing the growth defect without affecting the magnesium content of the Δ*corA*Δ*mgtA* strain (Figs 3 and 5). Cells lacking both CorA and MgtA may take more time to acquire enough Mg^2+^, leading to a growth defect. However, since the Δ*corA*Δ*mgtA* LCV variant had a normal growth despite its lower Mg^2+^ level, this hypothesis is unlikely. MgtA has been shown to increase the amount of activated PhoP molecules in a PhoQ-dependent manner, by removing Mg^2+^ from the periplasmic space, where it inhibits PhoQ activity [10]. One possible scenario to account for the differential growth phenotypes of the two Δ*corA*Δ*mgtA* variants is that the Δ*mgtA* mutation impairs growth of the Δ*corA* mutant by limiting PhoP activation and the phoQ*_R16S_ mutation alleviates this defect by favoring somehow PhoP activity.

### The effect of the Δ*corA* mutation on the proteome and motility of *Salmonella* is majored in the absence of PhoP

To determine why combination of the Δ*corA* and Δ*phoP* mutations, but not the mutations alone, impaired growth, we asked whether combination of these mutations was accompanied by a differential protein cargo content. A global quantitative proteomic analysis was carried out to compare the proteome of the wild-type strain, and the Δ*corA*, Δ*phoP* and Δ*corA*Δ*phoP* mutants, using five biological replicates of each strain (S1 Dataset and S17 Fig). Bacteria were grown to stationary phase in LB supplemented with magnesium 10 mM because the growth defect of the Δ*corA*Δ*phoP* strain was similar in LB and in LB supplemented with magnesium (Figs 5 and S9) but this latter condition aggravated the competitive growth disadvantage of the Δ*corA* mutant (S12 Fig) and increased the difference in MgtA amounts between the wild-type strain and the Δ*corA* mutant (Figs 4 and S7). A total of 2688 proteins were identified (“ID Table” in S1 Dataset), representing at least 50% of the reference proteome of strain ATCC14028 (UniProt–Taxon ID 588858, https://www.uniprot.org/taxonomy/588858). The remaining proteins might not be expressed under the growth condition employed in this study, or they might be expressed at levels too low to be detected. Also, membrane proteins are less easily identified by MS-based proteomics than soluble proteins.

#### Combination of the Δ*corA* and Δ*phoP* mutations had a profound effect on the *Salmonella* proteome

**Fig 6:**
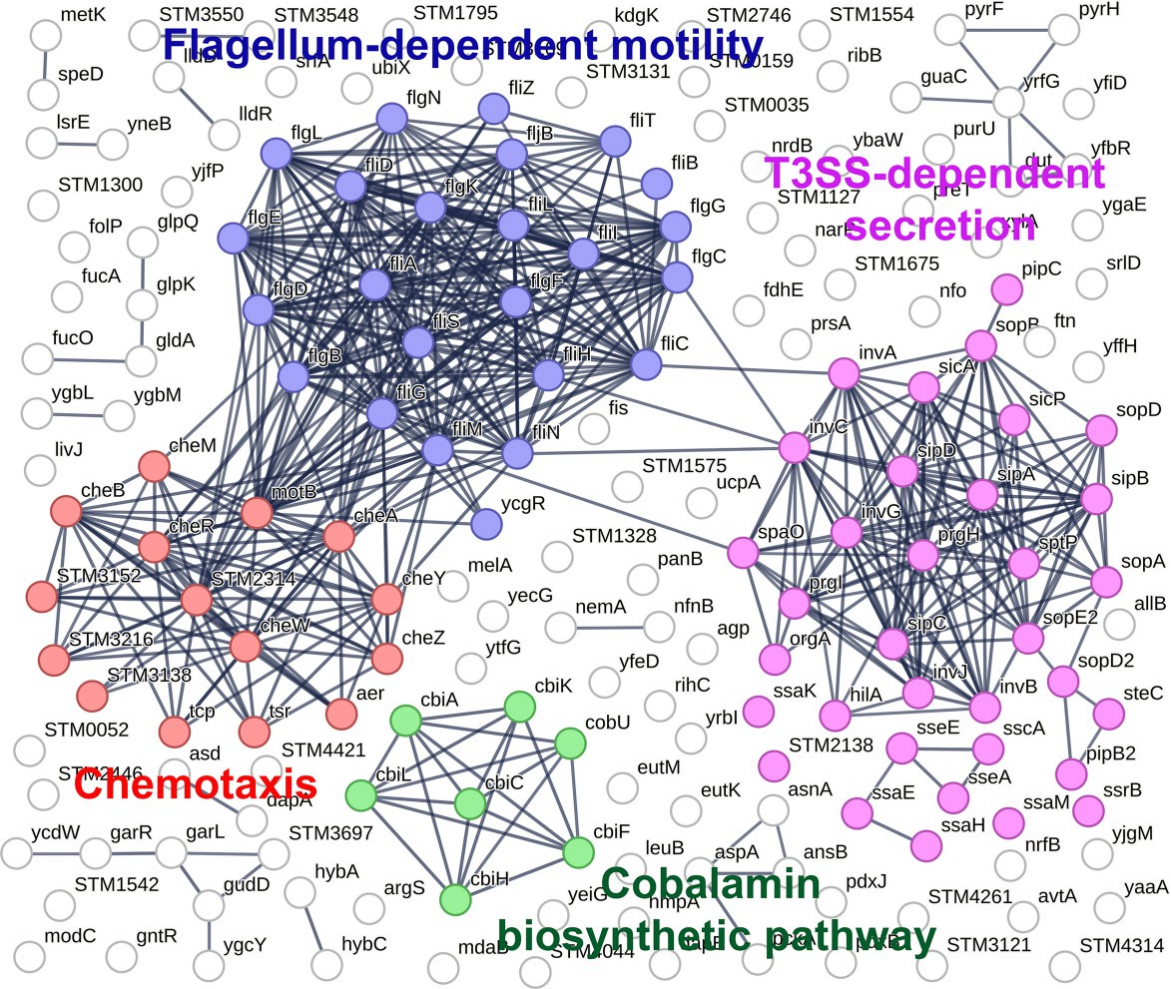
Protein-protein interaction network of the proteins significantly less abundant in the Δ*corA*Δ*phoP* mutant, but not found less abundant in the Δ*corA* and Δ*phoP* strains, when comparing to the wild-type strain. Top enriched functional categories of proteins are colored: flagellum-dependent motility in blue, chemotaxis in red, T3SS-dependent secretion in pink and cobalamin biosynthetic pathway in green. See also S2 Dataset for details.

Almost five hundred proteins of various functions were found differentially abundant between the Δ*corA*Δ*phoP* mutant and the wild-type strain (S1 Dataset). Then, enrichment analyses were performed to assess which relevant functional or mechanistic biological processes were significantly enriched in the lists of proteins differentially abundant between the mutants and the wild-type strain. Interestingly, it appears that many proteins less abundant in the Δ*corA*Δ*phoP* mutant than in the wild type strain and not found less abundant in the Δ*corA* and Δ*phoP* strain are related to flagella formation, chemotaxis, T3SS-dependent secretion, and cobalamin biosynthesis (Fig 6). A large number of proteins involved in these pathways were affected by the Δ*corA* and Δ*phoP* mutations combined, but not alone (S1 Dataset and S17 Fig).

**Fig 7:**
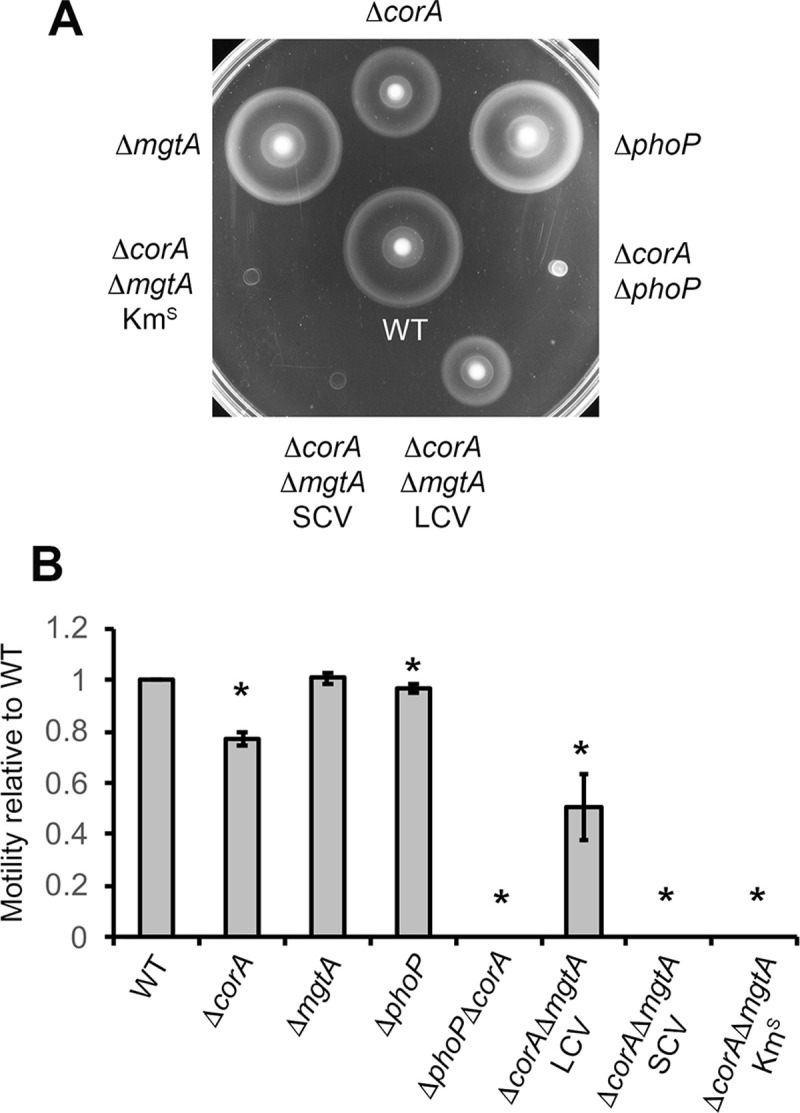
Motility of *Salmonella* wild-type and mutant strains. Motility of the wild-type (WT) strain and mutants indicated was assessed on LB agar 0.3% supplemented with MgCl_2_ 10 mM (Material and Methods). (A) A representative experiment is shown. (B) The diameter of the halo was measured after five hours of growth at 37°C. Since the diameters of halos were variable from one experiment to another, the diameter measured for a mutant strain is expressed relative to that measured for the wild-type strain within the same experiment. Bars represent the mean of at least three independent measurements and the error bars represent the standard error of the mean. * p-value <0.01.

PhoP has been reported to repress flagellum-encoding genes, thus preventing flagellum-mediated motility [10]. In our experimental conditions, there was no positive effect of the Δ*phoP* mutation alone on motility and flagellum synthesis (Fig 7 and S1 Dataset and S17 Fig). On its side, the Δ*corA* mutation alone slightly decreased *Salmonella* locomotion (Figs S2 and 7), but proteins involved in this pathway were not significantly affected by the Δ*corA* mutation (S1 Dataset). Several proteins involved in flagella biosynthesis and chemotaxis were less abundant in the Δ*corA*Δ*phoP* mutant (Fig 6 and S1 Dataset). Consistent with this finding, the Δ*corA*Δ*phoP* mutant was not motile (Fig 7) and contained decreased levels of the CheR and CheY proteins involved in chemotaxis (S18 Fig). Similarly, whereas there was no significant effect of the Δ*mgtA* mutation on motility and abundance of the CheR and CheY proteins, the Δ*corA*Δ*mgtA* SCV mutant was non-motile and had reduced levels of the CheR and CheY proteins (Figs 7 and S18). Of note, the Δ*corA*Δ*mgtA* LCV retained some motility (Fig 7) and contained higher amounts of CheR and CheY than the Δ*corA*Δ*mgtA* SCV (S18 Fig). As suggested above to explain the growth defect of the Δ*corA*Δ*mgtA* mutant, the Δ*mgtA* mutation may impair motility and expression of the CheRY proteins in the absence of CorA by limiting PhoP activation, an effect that would be alleviated by the phoQ*_R16S_ allele present in the Δ*corA*Δ*mgtA* LCV.

Under magnesium starvation, PhoP represses genes of SPI-1, encoding the T3SS-1 implicated in invasion of epithelial cells, which may explain why a PhoP-null mutant enters epithelial cells better than the wild type strain [10]. In contrast, PhoP promotes transcription of genes encoded by SPI-2, encoding the T3SS-2 implicated in macrophage survival and virulence [10]. Genes encoding components of SPI-1 and SPI-2 were repressed in a microarray study with a *Salmonella corA* mutant grown to log phase in rich medium [17]. In our experimental conditions, functional categories of proteins involved in secretion via T3SS were not found significantly enriched in the Δ*phoP* and Δ*corA* mutants, but they were enriched in the Δ*corA*Δ*phoP* strain (Figs 6 and S17 and S1 Dataset). Indeed, several proteins secreted or involved in secretion *via* T3SS were less abundant in the Δ*corA*Δ*phoP* mutant, an outcome which might be explained by the fact that HilA and SsrB proteins, the major transcriptional activator of SPI-1 and SPI-2 genes, respectively [54, 55], were less abundant in the Δ*corA*Δ*phoP* mutant (S1 Dataset).

#### Impact of the Δ*phoP* and Δ*corA* mutations alone

A large set of proteins was affected by the Δ*phoP* mutation alone, confirming that PhoP was functional in the wild-type strain grown to stationary phase in LB supplemented with magnesium 10 mM (S1 Dataset). Some of these proteins are known to be positively controlled by PhoP under magnesium starvation (PagC, RstA, IraP, ArnB, SseB, SlrP, BasR, S1 Dataset) [10]. The response to Mg^2+^-starvation orchestrated by PhoPQ is complex, with transcription of individual genes being induced at specific stages of the stress [56]. The pattern of transcription activation by PhoP is not dictated solely by the affinity of phosphorylated PhoP for its target promoters but it is also influenced by additional regulatory mechanisms and modulation of PhoP and PhoQ activity [10, 57]. Under the magnesium-proficient growth conditions used in this study, PhoP is probably activated through another environmental signal than magnesium starvation. PhoP is known to respond to other signals, including Ca^2+^, low pH and antimicrobial peptides [10] and additional signals are probably to be discovered, especially when the extracellular concentration of magnesium is high.

A major role for PhoP is the control of the cell envelope composition and regulation of LPS modifications which help bacteria to evade the immune system or confer resistance to antibacterial compounds or cation toxicity by lowering the negative charge of the membrane [10]. With regard to magnesium, modifications affecting lipid A phosphate residues release Mg^2+^ neutralizing ions, allowing the membrane to be a Mg^2+^ reservoir that can be used under magnesium deprivation [10]. Sixty-five proteins affecting membrane functions and LPS were more abundant in the Δ*phoP* mutant than the wild-type strain, indicating a cell wall remodeling in the absence of PhoP even when magnesium is available (S1 Dataset). The effect of combination of the Δ*corA* and Δ*phoP* mutations on the composition of the cell envelope was even more drastic with more than one hundred proteins more abundant (S1 Dataset). Several of these proteins are involved in transport activities and most are intrinsic components of membrane (S1 Dataset).

The effect of the Δ*corA* mutation on *Salmonella* proteome was more subtle than that of the Δ*phoP* mutation, with fifty-three proteins found differentially abundant with the wild-type and involved in various functions (S1 Dataset). Of note, among the fifty-three proteins affected by the Δ*corA* mutation, proteins involved in pyrimidine and propanediol metabolism were enriched and significantly less abundant in the Δ*phoP* and Δ*corA*Δ*phoP* mutants (S1 Dataset and S17 Fig). Although the biological significance of these effects, common to the Δ*phoP* and Δ*corA* mutations, must be determined, they suggest the existence of a certain level of functional redundancy between the CorA and PhoP systems under the experimental conditions used. The Δ*corA* mutation increases the abundance of MgtA whether PhoP is present or not (Figs 1 and 4 and S7). In agreement with these immunodetection data, calculation of the relative abundance of the MgtA protein from distinct peptides through the MS-based proteomic analysis validated the quantification of MgtA in the Δ*corA* and Δ*corA*Δ*phoP* samples, while not in the wild-type and Δ*phoP* samples (see details in S1 Text and S19 and S20 Figs).

Altogether, these findings suggest that under the experimental conditions used, the CorA and PhoP systems have compensatory effects important for the resilience of Δ*corA* and Δ*phoP* mutants.

## Conclusion

**Fig 8:**
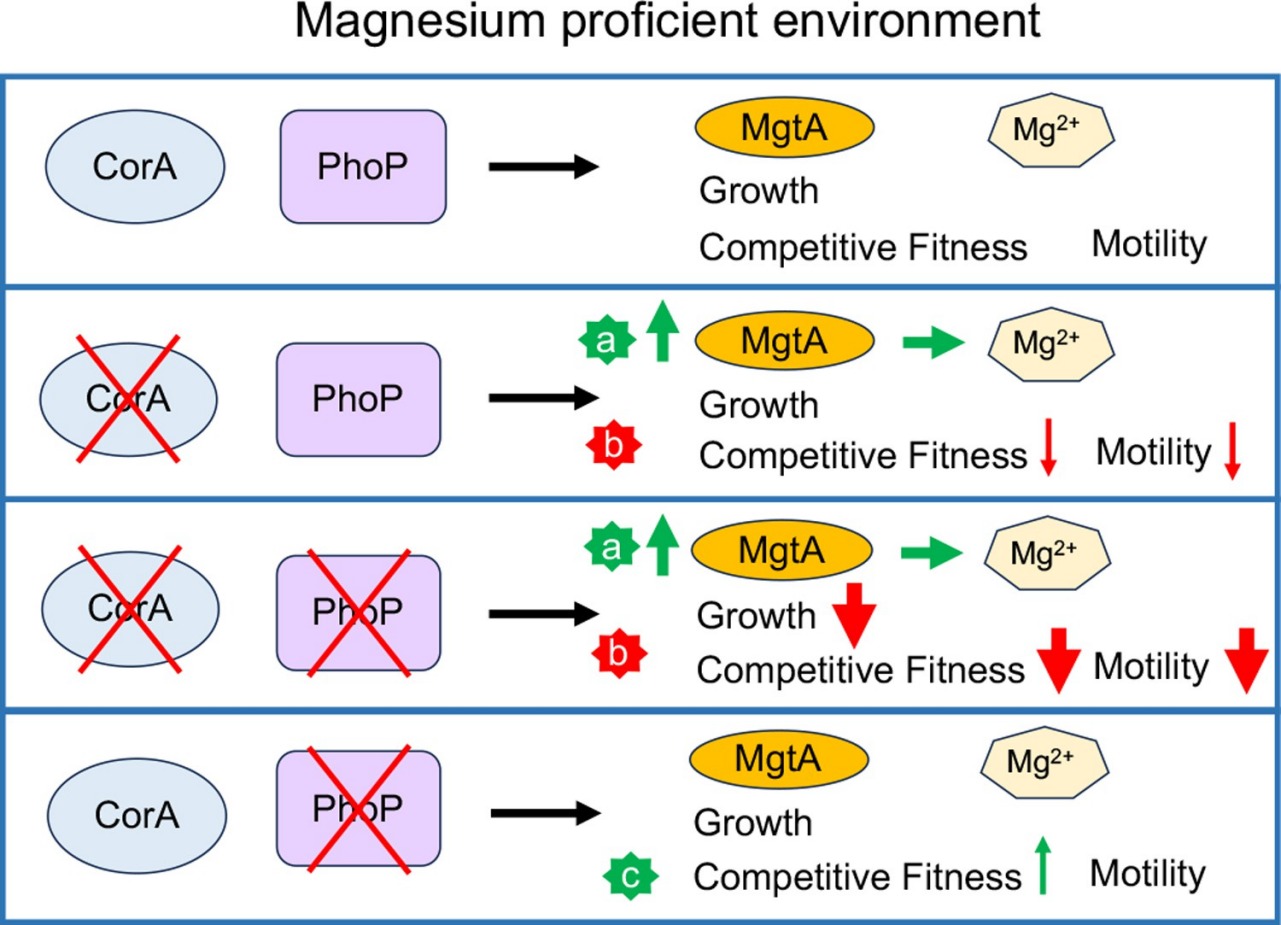
Schematic illustration of the synthetic effects of the Δ*corA* and Δ*phoP* mutations on *Salmonella* physiology. When magnesium is sufficient, the housekeeping CorA protein is the main Mg^2+^ transporter. PhoP is a global transcriptional regulator that promotes expression of the other two Mg^2+^-transporters, MgtA and MgtB, under magnesium starvation. Vertical arrows indicate positive (green) or negative (red) effects of the Δ*corA* and Δ*phoP* mutations on the indicated phenotypes of *Salmonella*. The line thickness is indicative of the magnitude of the effect. When the *corA* gene is deleted, MgtA production increased in a PhoP-independent fashion, which alleviates the negative effect of the Δ*corA* mutation on magnesium homeostasis (a). The exact mechanisms by which the Δ*corA* mutation promotes MgtA production is unknown (see text). When the Δ*corA* mutation is combined with the Δ*phoP* mutation, synthetic phenotypes are observed, including reduction in growth in monoculture, competitive fitness, and motility (b). It is unknown so far whether the synthetic growth phenotype of the Δ*corA*Δ*phoP* mutant and the phenotype of the Δ*corA* mutant in competitive fitness to exit from stationary phase rely on the same mechanism. A synthetic growth phenotype is also observed when the Δ*corA* mutation is combined with the Δ*mgtA* mutation, unless the phoQ*_R16S_ allele is present (see text for details). This phenomenon may be due to a reduced level of PhoP activated molecules in the Δ*corA*Δ*mgtA* mutant, alleviated somehow by the phoQ*_R16S_ mutation.

This study suggests the existence of a functional redundancy between the CorA and PhoP systems beyond magnesium trafficking and unravels the importance of PhoP and MgtA in the resilience of the Δ*corA* mutant (Fig 8). MgtA, but not PhoP, is necessary to maintain magnesium homeostasis in the absence of CorA under non-limiting extracellular magnesium levels (Fig 8A). How does the absence of CorA promote PhoP-independent MgtA production? *mgtA* expression is activated under conditions of Mg^2+^ starvation at the levels of transcription initiation *via* PhoP and transcription elongation through a magnesium-sensing mRNA leader [10, 45–47, 53]. However, MgtA production in the Δ*corA* mutant appears insensitive to high external concentration of magnesium, is independent of PhoP, and is not due to variations in total and free cell magnesium contents. Overexpression of Rob, a transcriptional regulator, promotes *mgtA* transcription in a PhoP-independent fashion [58]. However, experiments using transcriptional and translational *mgtA-lacZ* fusions suggests that *mgtA* regulation by the Δ*corA* mutation is post-transcriptional (S21 Fig). Identification of the molecular bases underlying this phenomenon will thus add a novel level of complexity to MgtA regulation [10]. On its side, PhoP is required for growth, competitive fitness, and motility, in the absence of CorA only and even under high magnesium conditions (Fig 8B). Under these conditions, synthetic effects of the Δ*corA* and Δ*phoP* mutations were observed on the abundance of proteins involved in flagellum-dependent motility, chemotaxis, secretion, and cobalamin biosynthesis. It is not known so far if, and how, this proteome rewiring contributes to the synthetic growth phenotype of the Δ*corA*Δ*phoP* mutant. It will be also interesting to determine whether the synthetic growth phenotype of the Δ*corA*Δ*phoP* and Δ*corA*Δ*mgtA* mutants and the competitive fitness disadvantage of the Δ*corA* mutant in exiting stationary phase rely on the same molecular mechanism. Altogether, our data unravel the existence of a regulatory network involving at least two magnesium transporters and a global regulator. It remains to be determined how CorA is connected to the PhoPQ system and the possible role(s) of MgtA (Fig 8). The absence of CorA may be sensed by the cell through the loss of physical interactions between CorA and membrane or cytoplasmic proteins, yielding to MgtA production and PhoP activation. Alternatively, a novel function of CorA, besides Mg^2+^ trafficking, might be involved.

CorA proteins are a unique class of transporters and are widespread in the Bacteria and Archaea, with functional homologs in eukaryotes. Many questions about CorA and its full role in *Salmonella* physiology and pathogenicity remain unanswered.

The Mg^2+^ Km values for the CorA, MgtA and MgtB transporters are in the same order of magnitude [14] and CorA is expressed in many environmental conditions, including the low Mg^2+^ environments where the other two transporters are expressed. What is then the logic behind this functional redundancy? CorA might be not active, or its activity might be harmful under conditions inducing the PhoP regulon, such as low Mg^2+^ environments. An alternative view supported by this study is that this functional redundancy is essential for *Salmonella* resilience, whereby robustness is conferred by a regulatory network between *corA* and *mgtA/phoP* rather than the individual genes. Understanding of how functional redundancy works and of how adaptive compensation mechanisms enhance the bacteria robustness is highly relevant for developing novel therapeutics.

## Supporting information

S1 Dataset Proteomic data. (XLSX)

S2 Dataset Details for protein-protein interaction network on Fig 6. (XLSX)

S1 Table Strains used in this study. (PDF)

S2 Table Primers used in this study. (PDF)

S1 Text Evaluation of the relative abundance of MgtA by the MS-based proteomic analysis (see also S19 and S20 Figs). (PDF)

S1 Fig Immunodetection of MgtB and MgtC in stationary phase *Salmonella*. The MgtB-Flag (A) and MgtC-Flag (B) proteins were immunodetected in the wild-type (WT) and mutant strains grown for 18h at 37°C in LB supplemented or not with EDTA 2 mM. Membranes used to reveal the Flag-tagged proteins with the anti-Flag antibody were then incubated in the presence of antibodies directed against the alpha subunit of RNA polymerase used as a loading control of total protein amounts. The Mg^2+^-transporter MgtB is a 101-kDa protein. MgtC is a 22.5-kDa protein encoded by the first gene of the *mgtCB* operon which inhibits ATP synthesis and protects PhoP from proteolysis [10].
(TIF)

S2 Fig Motility of *Salmonella* ATCC14028 and mutant derivatives. (A) Motility of the wild-type (WT) strain and Δ*rpoS* and Δ*corA* mutants. (B) Motility of the Δ*rpoS* and Δ*rpoS*Δ*corA* mutants. (C) The Δ*corA* mutation was complemented with the pAC*corA* plasmid containing the *corA* gene, the pACYC184 vector being used as a control. Motility was estimated by measuring the diameter of the halo after five hours of growth at 37°C on LB agar 0.3%. Representative experiments are shown (A, B, C). Although the diameters of halos were variable from one experiment to another, the effect of the Δ*corA* mutation on the motility of the wild-type and Δ*rpoS* strains was reproducibly observed in the three independent experiments that were carried out.
(TIF)

S3 Fig Growth and stationary phase survival of the wild-type strain ATCC14028 and Δ*corA* mutant in LB. (A) Stationary phase survival of wild-type and Δ*corA* strains grown in LB was evaluated by measuring the colony forming units (CFU) daily for up to three days. One-hundred percent survival corresponds to the number of cells in cultures grown overnight (Time 0). (B) Kinetics of growth of wild-type and Δ*corA* strains in LB was followed by measuring the optical density of bacterial cultures at 600 nm. (C) Kinetics of growth of wild-type and Δ*corA* strains in LB was followed by measuring viable cells on LB plates. Stationary phase (18 h) LB cultures were inoculated into fresh LB (about 3000 cells/ml) and the colony forming units (CFU) were measured at time intervals. Two independent experiments were performed using biological replicates (A-C).
(TIF)

S4 Fig Cell-associated magnesium content during *Salmonella* growth. Stationary phase LB cultures of the wild-type (WT) and Δ*corA* strains were diluted into fresh LB medium and the cell-associated magnesium content (panel C) was measured during the lag phase of growth (LAG, red line) and until entry to stationary phase (blue star). The kinetics of growth was followed by measuring the OD_600_ of the culture (panel A) and the viable cells (panel B). σ^S^ was immunodetected to similar levels in wild-type and Δ*corA* cells (panel D). σ^S^ was detected only in the inoculum and at the entry to stationary phase (from OD_600_ of about 1), as expected [1]. GroEL was immunodetected as a loading control. The error bars represent standard errors for three independent measurements. No significant difference between the wild-type strain and the Δ*corA* mutant was found (A-D).
(TIF)

S5 Fig Magnesium content in the growth medium following *Salmonella* inoculation. Stationary phase LB cultures of the wild-type (WT) strain and Δ*corA* mutant were diluted into fresh LB medium and the extracellular magnesium content was measured during *Salmonella* growth (nmoles/*μ*l, panel C). The kinetics of growth was followed by measuring the OD_600_ of the culture (panel A) and the viable cells (CFU/ml, panel B). Two independent experiments were conducted. Magnesium concentration in various LB batches varies from 0.1 to 0.2 mM [8] and was around 0.11 mM in Experiment 1 and 0.16 mM in Experiment 2. In both experiments, magnesium concentration was stable during the lag phase of growth (LAG, blue line).
(TIF)

S6 Fig Intracellular free magnesium measurements. *Salmonella* wild-type and mutant strains were grown for 18 h at 37°C in LB. Cell-associated free magnesium amounts were measured as described (Material & Methods). The fluorescence ratio per OD_600_ of the bacterial culture was estimated for each strain tested using three biological replicates. Relative fluorescence values, *i*.*e*., fluorescence ratio per OD_600_ for a mutant relative to the fluorescence ratio per OD_600_ for the wild-type strain (A, B) or the Δ*rpoS* mutant (C) evaluated within the same experiment, were used for comparison between strains through independent experiments. For each strain comparison (A-C) the three independent experiments performed are shown with different colors and p-values are indicated. In these experiments, the only significant difference was between the wild-type strain and the Δ*rpoS* mutant (panel A).
(TIF)

S7 Fig Regulation of MgtA production. The MgtA-Flag protein was immunodetected in *Salmonella* wild-type (WT) and mutant strains grown for 18 h at 37°C in LB supplemented or not with MgCl_2_ 10 mM (A, B) or EDTA 2 mM (B). MgtA is a 95-kDa protein. In most immunodetection experiments, two MgtA-Flag products were found (a full-length product at about 98-kDa and a smaller product of about 38-kDa), suggesting that the MgtA-flag protein is sensitive to degradation. Membranes used to reveal the Flag-tagged proteins with the anti-Flag antibody were then incubated in the presence of antibodies directed against GroEL used as a loading control of total protein amounts.
(TIF)

S8 Fig The plasmid-borne *corA* gene restores wild-type growth of the Δ*corA*Δ*phoP* mutant. The *Salmonella* wild-type and Δ*corA*Δ*phoP* strains harboring the vector pACYC184 and derivatives containing the *corA* gene (pAC*corA* OR1 and OR2, S1 Table) were grown 18 h in LB at 37°C. Cultures were streaked on LB plates which were incubated at 37°C and colony size was examined overnight. A representative experiment is shown.
(TIF)

S9 Fig The Δ*corA* mutation impairs kinetics of cell growth in the absence of *phoP* only. The *Salmonella* wild-type (WT), Δ*corA*, Δ*phoP* and Δ*corA*Δ*phoP* strains were grown 18 h in LB (A, C) and in LB supplemented with magnesium 10 mM (B, C) at 37°C. Cultures were inoculated at the same OD_600_ into fresh LB supplemented or not with magnesium, as indicated. Growth was followed by measuring the optical density at 600 nm. Representative experiments are shown.
(TIF)

S10 Fig Competition experiments between the wild-type strain and the Δ*corA*F044*phoP and* Δ*phoP* mutants of *Salmonella*. (A) Competition assays between the wild-type strain ATCC14028 (WT) and the mutants indicated were performed in LB and in LB supplemented with MgCl_2_ 10 mM. Equal cell numbers of stationary phase cultures of the wild-type strain and the mutant strain were mixed in fresh medium to give a total of about 3000 cells ml-1 (Day 0) and the mixtures were incubated at 37°C with shaking. Aliquots of bacteria were removed at timed intervals and numbers of viable cells of each strain were determined. Cells number of each strain is reported as a percentage of the total number of viable cells in the culture. The error bars represent standard errors for three independent measurements. * Statistically significant competitive advantage or disadvantage of one strain compared to the other (p-value <0.01).
(TIF)

S11 Fig Competition experiments between the Δ*phoP* and the Δ*corA*F044*phoP* mutants of *Salmonella*. (A) Competition assays between the Δ*phoP* and Δ*corA*F044*phoP* mutants were performed in LB supplemented or not with MgCl_2_ 10 mM. Equal cell numbers of stationary phase cultures of each strain were mixed in fresh medium to give a total of about 3000 cells ml-1 (Day 0) and the mixtures were incubated at 37°C with shaking. Aliquots of bacteria were removed at timed intervals and numbers of viable cells of each strain were determined. For each time point, cells number of each strain is reported as a percentage of the total number of viable cells in the culture. (B) Mixture of the Δ*phoP* and Δ*corA*F044*phoP* cells at day 0 was spread on LB plates and incubated overnight at 37°C to visualize colony size.
(TIF)

S12 Fig Effect of magnesium on the competitive growth disadvantage of the Δ*corA* mutant of *Salmonella*. (A) Competition assays between the wild-type strain ATCC14028 (WT) and the Δ*corA* mutant were performed in LB supplemented or not with MgCl_2_ at the indicated concentrations and at 37°C. Equal cell numbers of stationary phase cultures of the wild-type strain and the mutant were mixed in fresh medium to give a total of about 3000 cells ml-1 (Day 0) and the mixtures were incubated at 37°C with shaking. Aliquots of bacteria were removed at Day 0 and Day 1 (after 24 h of growth) and numbers of viable cells of each strain were determined. Cells number of each strain is reported as a percentage of the total number of viable cells in the culture. The error bars represent standard errors for three independent quantifications from biological replicates. * Statistically significant competitive disadvantage of the Δ*corA* mutant compared to the wild-type (p-value <0.01). (B) Proportion of the Δ*corA* mutant at day 1 relative to day 0 in the different growth conditions. No effect of magnesium was observed at 0.1 mM or 1 mM. The competitive disadvantage of the Δ*corA* mutant was aggravated when LB was supplemented with magnesium at 10 mM (* p-value <0.01, n = 3). Under the same growth condition, the fitness of the control strain 2922K and the wild-type strain ATCC14028 was similar (C, n = 3).
(TIF)

S13 Fig Kinetics of growth of the Δ*corA*Δ*mgtA* small and large colony variants. The *Salmonella* wild-type strain, the Δ*mgtA* mutant and the Δ*corA*Δ*mgtA* large colony and small colony variants (LCV and SCV, respectively) were grown 18 h in LB at 37°C. The Δ*corA*Δ*phoP* was included as a control (S9 Fig). Cultures were inoculated at the same OD_600_ into fresh LB and growth was followed by measuring the optical density at 600 nm. Representative experiments are shown.
(TIF)

S14 Fig Effects of magnesium supplementation on growth of the Δ*corA*Δ*mgtA* mutants and their ability to compete with the wild-type strain. A) The *Salmonella* wild-type (WT), Δ*mgtA* and Δ*corA*Δ*mgtA* strains were grown 18 h at 37°C in LB supplemented with magnesium 10 mM. Cultures were inoculated at the same OD_600_ into fresh LB supplemented with magnesium and growth was followed by measuring the optical density at 600 nm. Representative experiments are shown. B) Competition assays between the wild-type strain ATCC14028 (WT) and the Δ*mgtA* strain or the Δ*corA*Δ*mgtA* mutants were performed in LB supplemented or not with MgCl_2_ 10 mM at 37°C. Equal cell numbers of stationary phase cultures of the wild-type strain and the mutant were mixed in fresh medium to give a total of about 3000 cells ml-1 (Day 0) and the mixtures were incubated at 37°C with shaking. Aliquots of bacteria were removed at timed intervals and numbers of viable cells of each strain were determined. Cells number of each strain is reported as a percentage of the total number of viable cells in the culture. The error bars represent standard errors for three independent quantifications from biological replicates. * Statistically significant competitive disadvantage of the Δ*corA*Δ*mgtA* mutant compared to the wild-type (p-value <0.01).
(TIF)

S15 Fig Colony size of strains containing the Δ*mgtA* and *mgtA*-3xflag constructs. The *Salmonella* wild-type strain and mutants indicated were grown 18 h in LB at 37°C. Cultures were spread on LB plates which were incubated at 37°C and colony size was examined overnight. Representative experiments are shown.
(TIF)

S16 Fig The Δ*corA* mutation impairs *Salmonella* growth in the absence of PhoQ. The *Salmonella* wild-type, Δ*corA*, Δ*phoQ* and Δ*corA*Δ*phoQ* strains were grown 18 h in LB at 37°C. Cultures were spread on LB plates which were incubated at 37°C and colony size was examined overnight. The two Δ*phoQ* constructions are identical, except for the antibiotic resistance cartridge.
(TIF)

S17 Fig Enrichment analyses of GO terms for the effects of the Δ*corA* and Δ*phoP* mutations, alone and combined, on the *Salmonella* proteome. Enrichment analyses of GO terms have been performed from the lists of differentially abundant proteins between mutants and the wild-type strain (WT). (A) Enrichment analysis for the proteins significantly more abundant in WT than Δ*phoP* (left), and significantly more abundant in Δ*phoP* than WT (right). (B) Enrichment analysis for the proteins significantly more abundant in WT than Δ*corA* (left), and significantly more abundant in Δ*corA* than WT (right). (C) Enrichment analysis for the proteins significantly more abundant in WT than Δ*corA*Δ*phoP* (left), and significantly more abundant in Δ*corA*Δ*phoP* than WT (right). Histograms represent the number of proteins associated to a GO term in a list. Colors are function of the p-value of enrichment of the associated GO term (the redder it is, the stronger the enrichment of the term). Grey color means the p-value of enrichment is superior to 1% (e.g., the GO term is not enriched). GO terms of interest are highlighted in bold.
(TIF)

S18 Fig Immunodetection of the CheR and CheY proteins in *Salmonella* strains carrying the Δ*corA*, Δ*phoP* and Δ*mgtA* mutations alone and combined. The CheR-Flag and CheY-Flag proteins were immunodetected in the *Salmonella* strains indicated grown for 18 h at 37°C in LB supplemented or not with MgCl2 10 mM. Membranes used to reveal the Flag-tagged proteins with the anti-Flag antibody were then incubated in the presence of antibodies directed against GroEL used as a loading control of total protein amounts. These immunodetection data are consistent with the MS-based data showing reduced abundance of the CheR and CheY proteins in the Δ*corA*Δ*phoP* mutant (Fig 6 and S1 Dataset). In addition, these data reveal a reduced abundance of the CheR and CheY proteins in the Δ*corA*Δ*mgtA* SCV mutant, compared to the Δ*corA*Δ*mgtA* LCV mutant.
(TIF)

S19 Fig Evaluation of the relative abundance of MgtA from distinct peptides through the MS-based proteomic analysis. (A) From the MaxQuant analysis, histogram representing intensities of identified peptides for MgtA. (B) MS1 and MS2 spectra. B1: Chromatogram of a wild-type sample (grey box) with the extracted ion chromatogram for 2+ (1164.6113 m/z) and 3+ (776.7433 m/z) charge states of the NLLDTAVLEGVDETAARQLSGR peptide (blue box). B2 (orange box): HCD fragmentation spectra, from database search using PEAKS software, attributed to the NLLDTAVLEGVDETAARQLSGR peptide. B3 (orange box): HCD fragmentation of a similar spectra, from de novo sequencing using PEAKS software, attributed to a RGWLATLEQVTLVDDTLAR peptide. See also S1 Text.
(PDF)

S20 Fig MS2 ion tables. (A) MS2 ion table corresponding to S16B2 Fig. (B) MS2 ion table corresponding to S16B3 Fig (B). The database search approach attributes 20 masses to NLLDTAVLEGVDETAARQLSGR peptide. The *de novo* sequencing approach attributes 51 masses to a RGWLATLEQVTLVDDTLAR peptide which is not registered in the database. Orange boxes indicate similar masses between database search and *de novo* sequencing approaches (8 masses). See also S1 Text.
(PDF)

S21 Fig Expression of *mgtA-lacZ* and *mgtB-lacZ* fusions in stationary phase *Salmonella*. Expression of the transcriptional and translational *mgtA-lacZ* fusions was evaluated in the wild-type and Δ*corA* strains, grown for 18 h in LB at 37°C. A translational *mgtB-lacZ* fusion was included as a control. Bar graphs represent the mean β-galactosidase activity, and error bars represent standard deviation of at least three independent experiments. The transcriptional and translational *mgtA-lacZ* fusions were both inserted at the beginning of the *mgtA* ORF (S2 Table). The translational *mgtB-lacZ* fusion was inserted at the beginning of the *mgtB* ORF (S2 Table). The *mgtA-lacZ* and *mgtB-lacZ* translational fusions were expressed to very low levels in the wild-type strain and expression of the *mgtA-lacZ* (but not *mgtB-lacZ*) fusion was increased in the Δ*corA* mutant, in agreement with the immunodetection data (Figs 1 and S1). The strong positive effect of the Δ*corA* mutation on *mgtA* expression was not observed with the transcriptional *mgtA-lacZ* fusion, suggesting a post-transcriptional effect.
(TIF)

S1 Raw images (PDF)

S2 Raw images (PDF)

S3 Raw images (PDF)

S4 Raw images (PDF)

S5 Raw images (PDF)

S6 Raw images (PDF)

S7 Raw images (PDF)

S8 Raw images (PDF)

S9 Raw images (PDF)

S10 Raw images (PDF)

S11 Raw images (PDF)

S12 Raw images (PDF)

S13 Raw images (PDF)
