# Supplementary material for: Loss of CorA, the primary magnesium transporter of Salmonella, is alleviated by MgtA and PhoP-dependent compensatory mechanisms
Source: PLoS One. 2023 Sep 15;18(9):e0291736. doi: 10.1371/journal.pone.0291736 (PMC10503707; doi:10.1371/journal.pone.0291736)
Supplement: S1 Table — (PDF) [file pone.0291736.s003.pdf]

S1 Table: Bacterial strains and plasmids used in this study

Strain or Plasmid	Characteristics	Source or reference
VF6910	ATCC14028 *Salmonella enterica* serovar Typhimurium, wild-type strain	American Type Culture Collection
VF7969	ATCC2922K	(26)
VF8158	ATCC14028 Δ*rpoS*ΔCm	(21)
VFC331	ATCC14028 Δ*rpoS* (scarless in frame deletion of *rpoS*)	(5)
VFG250	ATCC14028 Δ*corA*::Km	This study
VFG564	VFG250 with the Km cassette eliminated	This study
VFG562	VF8158 Δ*corA*::Km	This study
VFG563	VFG562 with the Km cassette eliminated	This study
VFH333	ATCC14028 Δ*phoP*::Km	This study
VFH437	VFH333 with the Km cassette eliminated	This study
VFH455	VFH437 Δ*corA*::Km	This study
VFH876	VFH437 with the Km cassette eliminated	This study
VFG592	ATCC14028 Δ*mgtA*::Cm	This study
VFH90	VFG592 with the Cm cassette eliminated	This study
VFH606	VFH90 Δ*corA*::Km, LCV	This study
VFH607	VFH606 with the Km cassette eliminated, LCV	This study
VFH938	VFH90 Δ*corA*::Km, SCV	This study
VFH977	VFH938 with the Km cassette eliminated, SCV	This study
VFG256	ATCC14028 *corA*-3xflag::Km	This study
VFG257	VF8158 *corA*-3xflag::Km	This study
VFF443	ATCC14028 *cbiO*-3xflag::Km	This study
VFF444	VF8158 *cbiO*-3xflag::Km	This study
VFG241	ATCC14028 *mgtA*-3xflag::Km	This study
VFG278	VF8158 *mgtA*-3xflag::Km	This study
VFG567	VFG564 *mgtA*-3xflag::Km	This study
VFI324	VFH437 *mgtA*-3xflag::Km	This study
VFI325	VFH876 *mgtA*-3xflag::Km	This study
VFG242	ATCC14028 *mgtB*-3xflag::Km	This study
VFG279	VF8158 *mgtB*-3xflag::Km	This study
VFG568	VFG564 *mgtB*-3xflag::Km	This study
VFG577	VFG563 *mgtB*-3xflag::Km	This study
VFG243	ATCC14028 *mgtC*-3xflag::Km	This study
VFG280	VF8158 *mgtC*-3xflag::Km	This study
VFG569	VFG564 *mgtC*-3xflag::Km	This study
VFI345	ATCC14028 Δ*phoQ*::Km	This study
VFI347	ATCC14028 Δ*phoQ*::Cm	This study
VFI346	VFG564 Δ*phoQ*::Km	This study
VFI348	VFG564 Δ*phoQ*::Cm	This study
VFI357	VFH607 Δ*phoQ*::Km	This study
VFI358	VFH977 Δ*phoQ*::Km	This study
VFI401	ATCC14028 *cheR*-3xflag::Km	This study
VFI402	ATCC14028 *cheR*-3xflag::Km	This study
VFI403	VFH437 *cheR*-3xflag::Km	This study
VFI404	VFH90 *cheR*-3xflag::Km	This study
VFI405	VFH876 *cheR*-3xflag::Km	This study
VFI406	VFH607 *cheR*-3xflag::Km	This study
VFI407	VFH977 *cheR*-3xflag::Km	This study
VFI408	ATCC14028 *cheY*-3xflag::Km	This study
VFI409	VFG564 *cheY*-3xflag::Km	This study
VFI410	VFH437 *cheY*-3xflag::Km	This study
VFI411	VFH90 *cheY*-3xflag::Km	This study
VFI412	VFH876 *cheY*-3xflag::Km	This study
VFI413	VFH607 *cheY*-3xflag::Km	This study
VFI414	VFH977 *cheY*-3xflag::Km	This study
VFH795	ATCC14028 *mgtA-lacZ*, translational fusion	This study
VFH797	VFG564 *mgtA-lacZ*, translational fusion	This study
VFH825	ATCC14028 *mgtA-lacZ*, transcriptional fusion	This study
VFH827	VFG564 *mgtA-lacZ*, transcriptional fusion	This study
VFH799	ATCC14028 *mgtB-lacZ*, translational fusion	This study
VFH801	VFG564 *mgtB-lacZ*, translational fusion	This study

**Plasmids**		
pACYC184	Cloning vector, Cm^R, Tet^R	(21)
pAC*corA* OR1	*corA*cloned into pACYC184, Cm^R	This study
pAC*corA* OR2	*corA* cloned into pACYC184, Cm^R	This study
