# Supplementary material for: Loss of CorA, the primary magnesium transporter of Salmonella, is alleviated by MgtA and PhoP-dependent compensatory mechanisms
Source: PLoS One. 2023 Sep 15;18(9):e0291736. doi: 10.1371/journal.pone.0291736 (PMC10503707; doi:10.1371/journal.pone.0291736)
Supplement: S2 Table — (PDF) [file pone.0291736.s004.pdf]

S2 Table. Oligonucleotides used in this study.

Name	Sequence (5' – 3')	Construction/Purpose
corA-flagCterFw	GGGGCTTGCGCCGTATTTGTACTTTAAGCGCAAGAACTGGCTGGACTACAAAGACCATGACGG	*corA*-3xFlag::Km
corA-flagCterRv	GGTGGTTCAGCCGCAGCTGAATCACCCTGGCCTTAATGTCTTACATATGAATATCCTCCTTAG	*corA*-3xFlag::Km
corA-P1	CGCATTGGGAGTCCCGGTCATGCTGAGCGCATTTCAACTGGAAGTGTAGGCTGGAGCTGCTTC	Δ*corA*::Km
corA-P2	CAGCCGCAGCTGAATCACCCTGGCCTTAATGTCTTACAGCCACATATGAATATCCTCCTTAG	Δ*corA*::Km
cbiO-flagFw	GTTTTTTCATCGAATGCAAAAGTGCGCATTCAGGGAGGCGTCAGACTACAAAGACCATGACGG	*cbiO*-3xFlag::Km
cbiO-flagRv	CCGACGTCGGATGCCGTCCCCTGCAACATAACTGCCTGCGTCACATATGAATATCCTCCTTAG	*cbiO*-3xFlag::Km
mgtA-flagFw	CCAGTTGGTGAAAGGGTTTTACAGCAGACGTTATGGCTGGCAGGACTACAAAGACCATGACGG	*mgtA*-3xFlag::Km
mgtA-flagRv	TCGGGGATTAAGCACGCTGGCGAATCCCCGACGAAAGTGTTTACATATGAATATCCTCCTTAG	*mgtA*-3xFlag::Km
mgtB-flagFw	AGGCATGAAGCGCTTTTATATCAAACGCTTTGGCCAGTGGTTTGACTACAAAGACCATGACGG	*mgtB*-3xFlag::Km
mgtB-flagRv	TATCGGGTGAGCGATTCATCTGGGCGATCCTCAAACATTATTACATATGAATATCCTCCTTAG	*mgtB*-3xFlag::Km
mgtC-flagFw	GATAACGATAATATCACCGCAATTCACTGGAGCATTGATAGTCAAGACTACAAAGACCATGACGG	*mgtC*-3xFlag::Km
mgtC-flagRv	CTTATACGCCTGGCGTAATGTTGCAATTGAATAAAAAACTATTACATATGAATATCCTCCTTAG	*mgtC*-3xFlag::Km
phoP-P1	CAAGGGAGAAGAGATGATGCGCGTACTGGTTGTAGAGGATAATGTGTAGGCTGGAGCTGCTTC	Δ*phoP*::Km
phoP-P2	AATTTATTCATTAGCGCAATTCAAAAAGATATVCTTGTCCGCGCATATGAATATCCTCCTTAG	Δ*phoP*::Km
mgtA-P1	CGCGGAGGGATTACCTATGCTAAAAATCATTACCCGCCAGCTTGTGTAGGCTGGAGCTGCTTC	Δ*mgtA*::Cm and transcriptional *mgtA-lacZ* fusion
mgtA-P2	CGGGGATTAAGCACGCTGGCGAATCCCCGACGAAAGTGTTTACATATGAATATCCTCCTTAG	Δ*mgtA*::Cm and transcriptional *mgtA-lacZ* fusion
phoQ-P1	AATGAATAAATTTGCTCGCCATTTTCTGCCGCTGTCGCTGCGGGTGTAGGCTGGAGCTGCTTC	Δ*phoQ*::Km et Cm
phoQ-P2	GTGGAAGAACGCACAGAAATGTTTATTCCTCTTTCTGTGTGGGCATATGAATATCCTCCTTAG	Δ*phoQ*::Km et Cm
corA-pACbam-Fw	AGGCTCGGATCCTGAGATGTTAATATTCTGAAATCATAGATAAATACTCTCA	*corA* cloned into pACYC184*
corA-pACbam-Rv	TTCCGAGGATCCTTACACCCTGGCCTTAATGTCTTACAGCCAGT	*corA* cloned into pACYC184*
cheR-flagFw	CCTGCGCGGACAGACGGTGTATGCGCTAAGTAAGGATAAAGCAGACTACAAAGACCATGACGG	*cheR*-3xFlag::Km
cheR-flagRv	ATTAGCGCGGAATCATCAACTGACAATACCCTGATTTTACTCACATATGAATATCCTCCTTAG	*cheR*-3xFlag::Km
cheY-flagFw	TCTGGAAGAGAAGCTCAACAAAATCTTTGAGAAACTGGGCATGGACTACAAAGACCATGACGG	*cheY*-3xFlag::Km
cheY-flagRv	ATCAGCAGGCTTGATAGATGGTTGCATCATCATCGCATCCTCACATATGAATATCCTCCTTAG	*cheY*-3xFlag::Km
mgtA-P4	GGAGGGATTACCTATGCTAAAAATCATTACCCGCCAGCTTTTTATTCCGGGGATCCGTCGACC	Translational *mgtA-lacZ* fusion
mgtA-P1R	GCGGTGAACCAGACGATAAGGCAAATGACGATTAAGCCGGGCGTGTAGGCTGGAGCTGCTTC	Translational *mgtA-lacZ* fusion
mgtB-P4	ATTCATCATGACTGACATGAACATTGAAAACCGGAAACTCAATATTCCGGGGATCCGTCGACC	Translational *mgtB-lacZ* fusion
mgtB-P1R	GAAATACTTTTTTATGCTGCTTATCATTTTCTGACGCCGGGCGGGTAGGCTGGAGCTGCTTC	Translational *mgtB-lacZ* fusion

* A PCR product carrying the *corA* gene was cloned into the BamHI restriction site located within the *tet* gene of pACYC184, into both orientations. In the OR1 orientation, the *corA* gene and the *tet* gene are transcribed in the same direction.
