# Supplementary material for: Loss of CorA, the primary magnesium transporter of Salmonella, is alleviated by MgtA and PhoP-dependent compensatory mechanisms
Source: PLoS One. 2023 Sep 15;18(9):e0291736. doi: 10.1371/journal.pone.0291736 (PMC10503707; doi:10.1371/journal.pone.0291736)
Supplement: S1 Text — (PDF) [file pone.0291736.s005.pdf]

S1 Text. Evaluation of the relative abundance of MgtA by the MS-based proteomic analysis (see also S19 and S20 Figs).

We employed a proteomic approach, integrating MaxQuant database search and statistical analysis, to investigate the presence and relative abundance of MgtA protein across different bacterial strains. The analysis of the S1 Dataset provided insights into the identification status of MgtA protein among the strains. The abundance of the MgtA protein was found to be similar in the four bacterial strains (S1 Dataset). This result was surprising since the immunodetection data clearly showed higher abundance of MgtA in the Δ*corA* and Δ*corA*Δ*phoP* mutants than in the wild-type and Δ*phoP* strains (Figs 1 and 4, S7 Fig). To address this issue, we examined in more details how the relative abundance of the MgtA protein was calculated from distinct peptides through the MS-based proteomic analysis (S19 and S20 Figs).

Peptide identification by MS/MS using a database involves comparing experimental MS/MS spectra with theoretical fragmentation patterns derived from a specific protein sequence database for defined bacterial strains. High collision-induced dissociation (HCD) or other fragmentation methods are used to generate the experimental MS/MS spectra from precursor ions. These spectra are then matched against theoretical spectra generated by in silico fragmentation of peptide sequences in the bacterial strain-specific database. The peptide sequence with the best match to the experimental spectrum is considered the identified peptide, providing insights into the presence and relative abundance of specific peptides. However, during MS/MS analysis, co-fragmentation of multiple precursor ions can occur when precursor ions with similar mass-to-charge ratios (m/z) are present in the sample. This can lead to overlapping or shared fragment ions, making it challenging to assign specific fragments to individual precursor ions accurately.

To calculate the relative abundance of MgtA protein, we identified four distinct peptides through MS-based proteomic analysis (see S19A Fig showing the distribution of the four peptides across the bacterial strains). Among the identified peptides, three, represented by the blue, grey, and yellow bars, were exclusively detected in the Δ*corA* and Δ*corA*Δ*phoP* strains (S19 Fig), where increased production of MgtA was observed by immunodetection (Figs 1 and 4, S7 Fig). One peptide, represented by the striped, orange bar, was identified in all bacterial strains and, as shown on the S19A Fig bar

chart, this peptide NLLDTAVLEGVDETAARQLSGR shows an atypical intensity distribution compared to the three other peptides. Therefore, MS1 spectral information were extracted to confirm the retention time and m/z of the peptide (S19B1 Fig). Using PEAKS software, similar HCD fragmentation spectra of the peptide reveal two different amino acid sequences based on a database search (S19B2 and S20A Figs) or based on a *de novo* sequencing method (S19B3 and S20B Figs). By evidence, there is a co-elution and a co-fragmentation of the NLLDTAVLEGVDETAARQLSGR and the RGWLATLEQVTLVDDTLAR peptides.

The *de novo* sequencing approach (with Peaks software) allows for the identification of more peak products compared to the database search approach. In our study, we observed that the RGWLATLEQVTLVDDTLAR peptide, which is not present in the database, cannot be searched by the MaxQuant software, thereby preventing it from competing with the NLLDTAVLEGVDETAARQLSGR peptide. Based on these observations, the RGWLATLEQVTLVDDTLAR peptide is identified as the most abundant peptide. However, since it coelutes with the NLLDTAVLEGVDETAARQLSGR peptide, the MaxQuant software overestimates the abundance of the MgtA peptide which is identified in all strains.

This analysis, validating the quantification by MS of the MgtA protein in the Δ*corA* and Δ*corA*Δ*phoP* samples, while not in the wild-type and Δ*phoP* samples, is in agreement with the immunodetection data of MgtA (Figs 1 and 4, S7 Fig).
