# Supplementary figures and images for: Loss of CorA, the primary magnesium transporter of Salmonella, is alleviated by MgtA and PhoP-dependent compensatory mechanisms
Source: PLoS One. 2023 Sep 15;18(9):e0291736. doi: 10.1371/journal.pone.0291736 (PMC10503707; doi:10.1371/journal.pone.0291736)

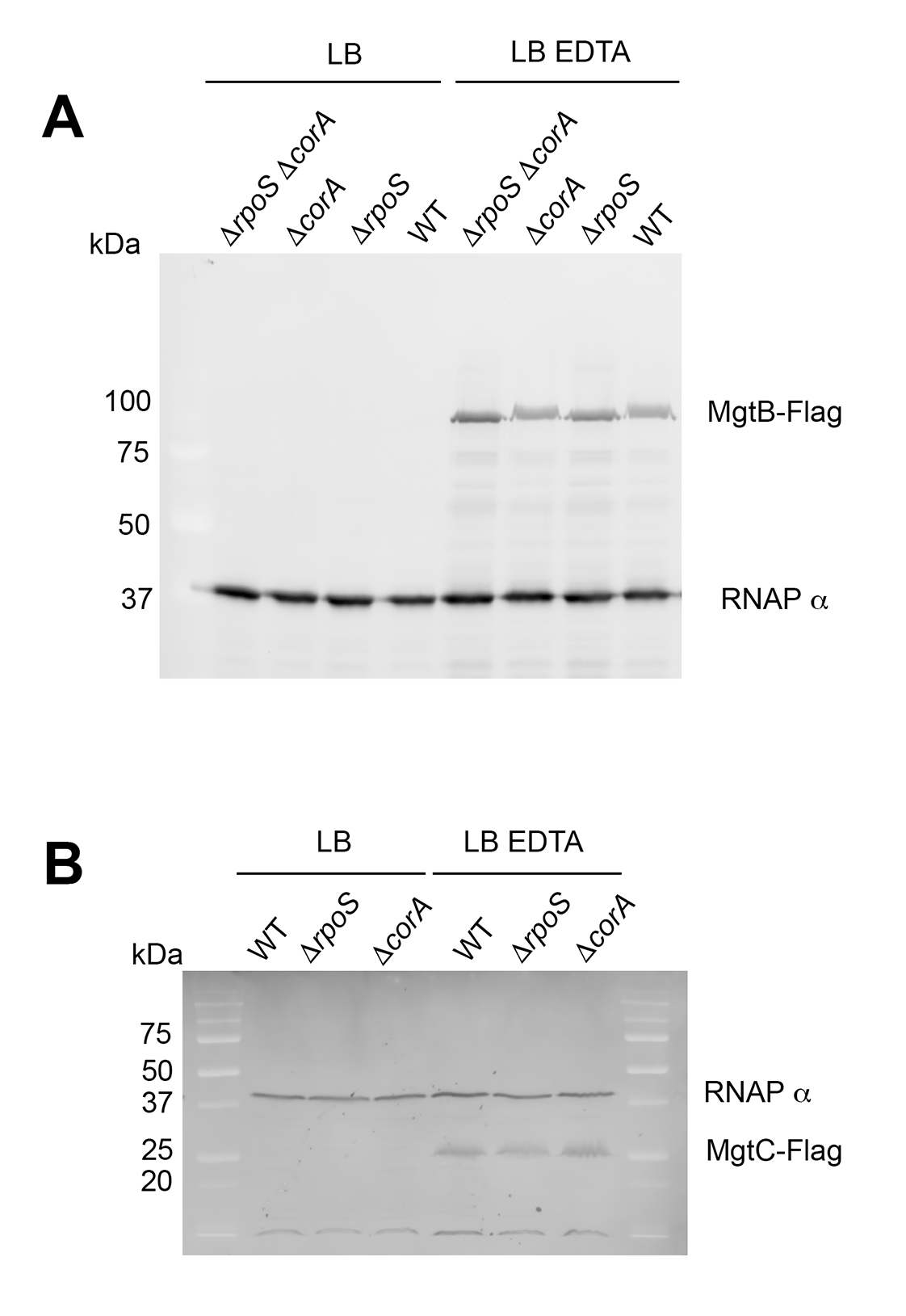

Supplement: S1 Fig — The MgtB-Flag (A) and MgtC-Flag (B) proteins were immunodetected in the wild-type (WT) and mutant strains grown for 18h at 37°C in LB supplemented or not with EDTA 2 mM. Membranes used to reveal the Flag-tagged proteins with the anti-Flag antibody were then incubated in the presence of antibodies directed against the alpha subunit of RNA polymerase used as a loading control of total protein amounts. The Mg2+-transporter MgtB is a 101-kDa protein. MgtC is a 22.5-kDa protein encoded by the first gene of the mgtCB operon which inhibits ATP synthesis and protects PhoP from proteolysis [10]. (TIF) [file pone.0291736.s006.tif]

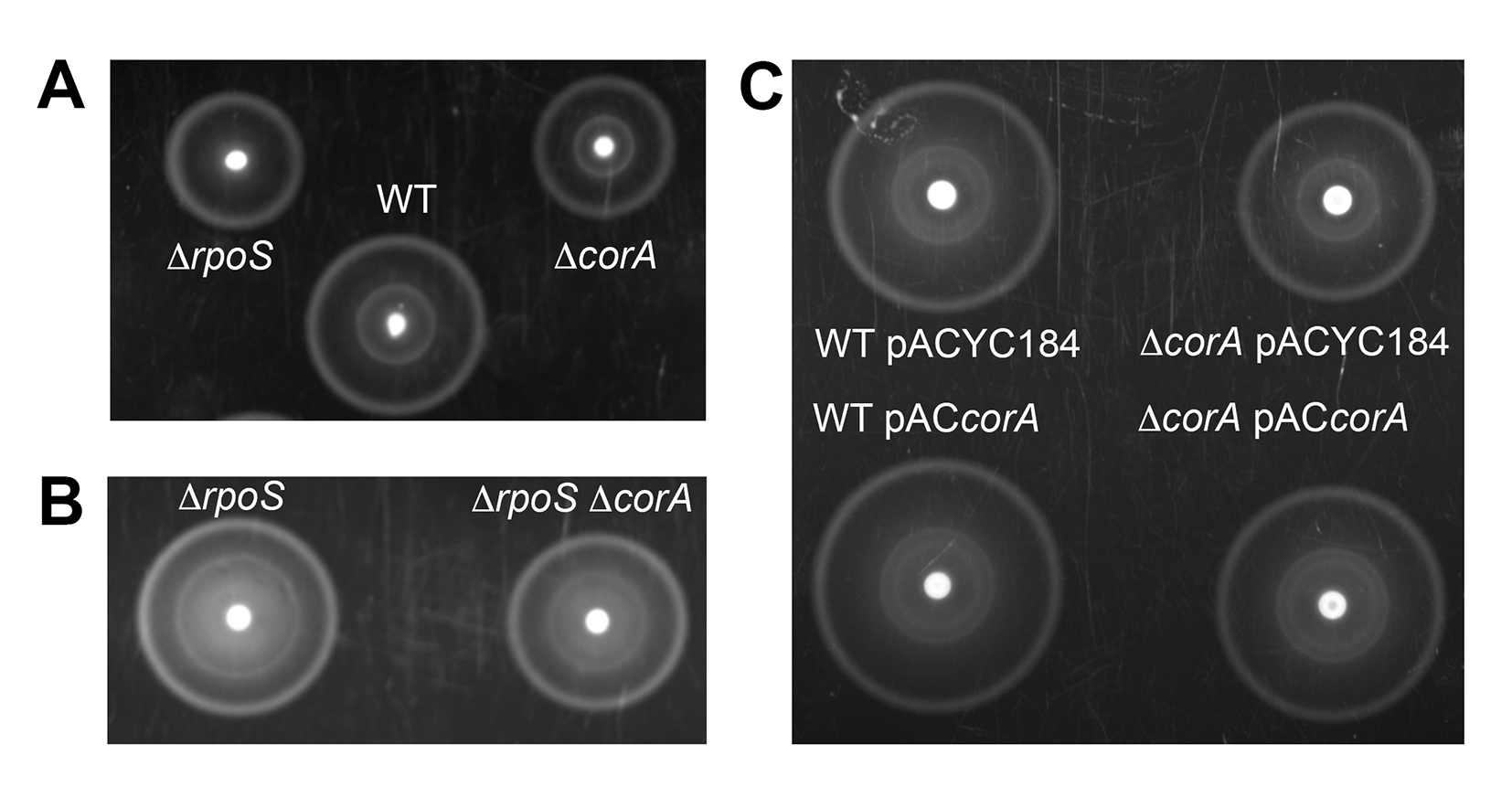

Supplement: S2 Fig — (A) Motility of the wild-type (WT) strain and ΔrpoS and ΔcorA mutants. (B) Motility of the ΔrpoS and ΔrpoSΔcorA mutants. (C) The ΔcorA mutation was complemented with the pACcorA plasmid containing the corA gene, the pACYC184 vector being used as a control. Motility was estimated by measuring the diameter of the halo after five hours of growth at 37°C on LB agar 0.3%. Representative experiments are shown (A, B, C). Although the diameters of halos were variable from one experiment to another, the effect of the ΔcorA mutation on the motility of the wild-type and ΔrpoS strains was reproducibly observed in the three independent experiments that were carried out. (TIF) [file pone.0291736.s007.tif]

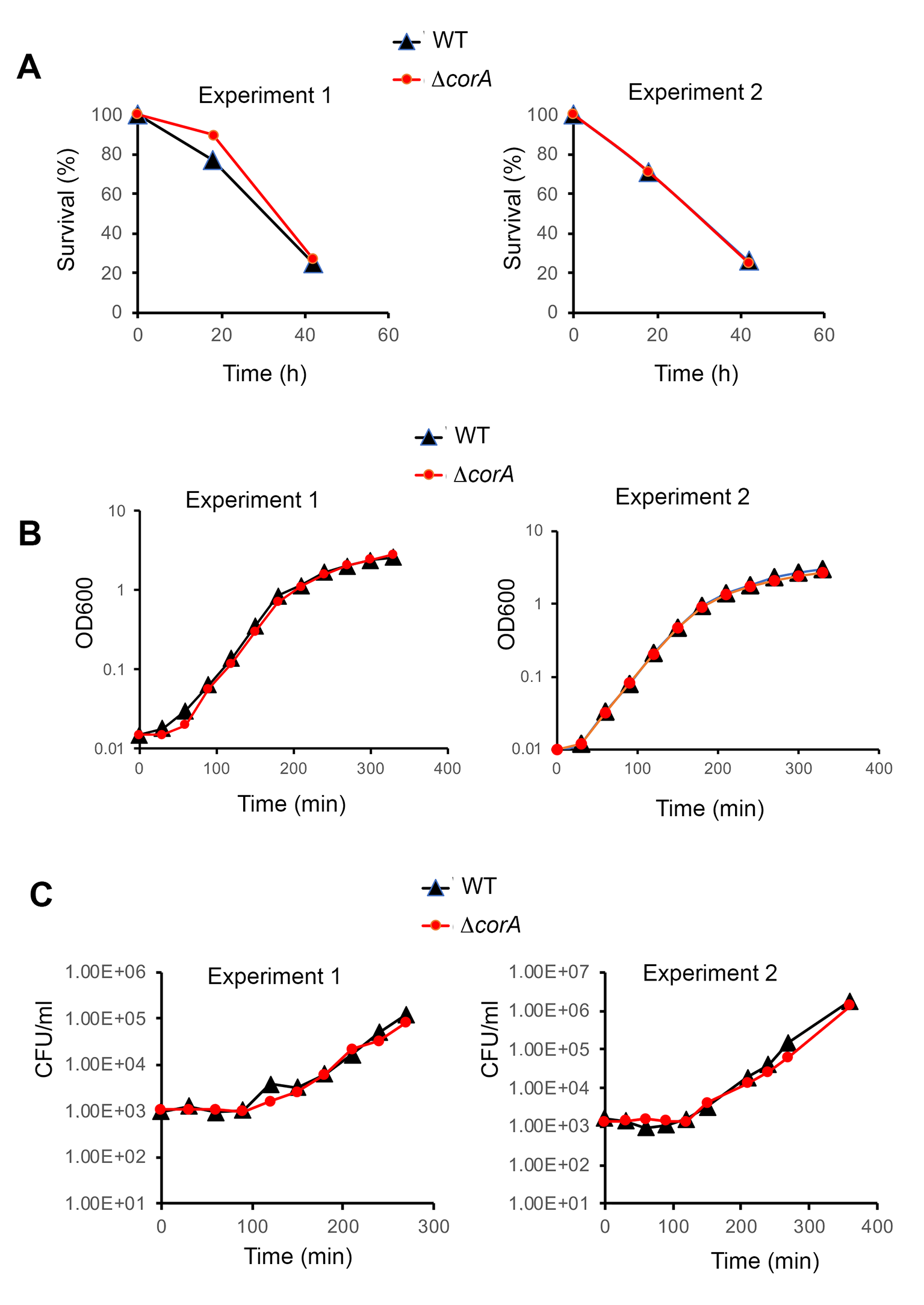

Supplement: S3 Fig — (A) Stationary phase survival of wild-type and ΔcorA strains grown in LB was evaluated by measuring the colony forming units (CFU) daily for up to three days. One-hundred percent survival corresponds to the number of cells in cultures grown overnight (Time 0). (B) Kinetics of growth of wild-type and ΔcorA strains in LB was followed by measuring the optical density of bacterial cultures at 600 nm. (C) Kinetics of growth of wild-type and ΔcorA strains in LB was followed by measuring viable cells on LB plates. Stationary phase (18 h) LB cultures were inoculated into fresh LB (about 3000 cells/ml) and the colony forming units (CFU) were measured at time intervals. Two independent experiments were performed using biological replicates (A-C). (TIF) [file pone.0291736.s008.tif]

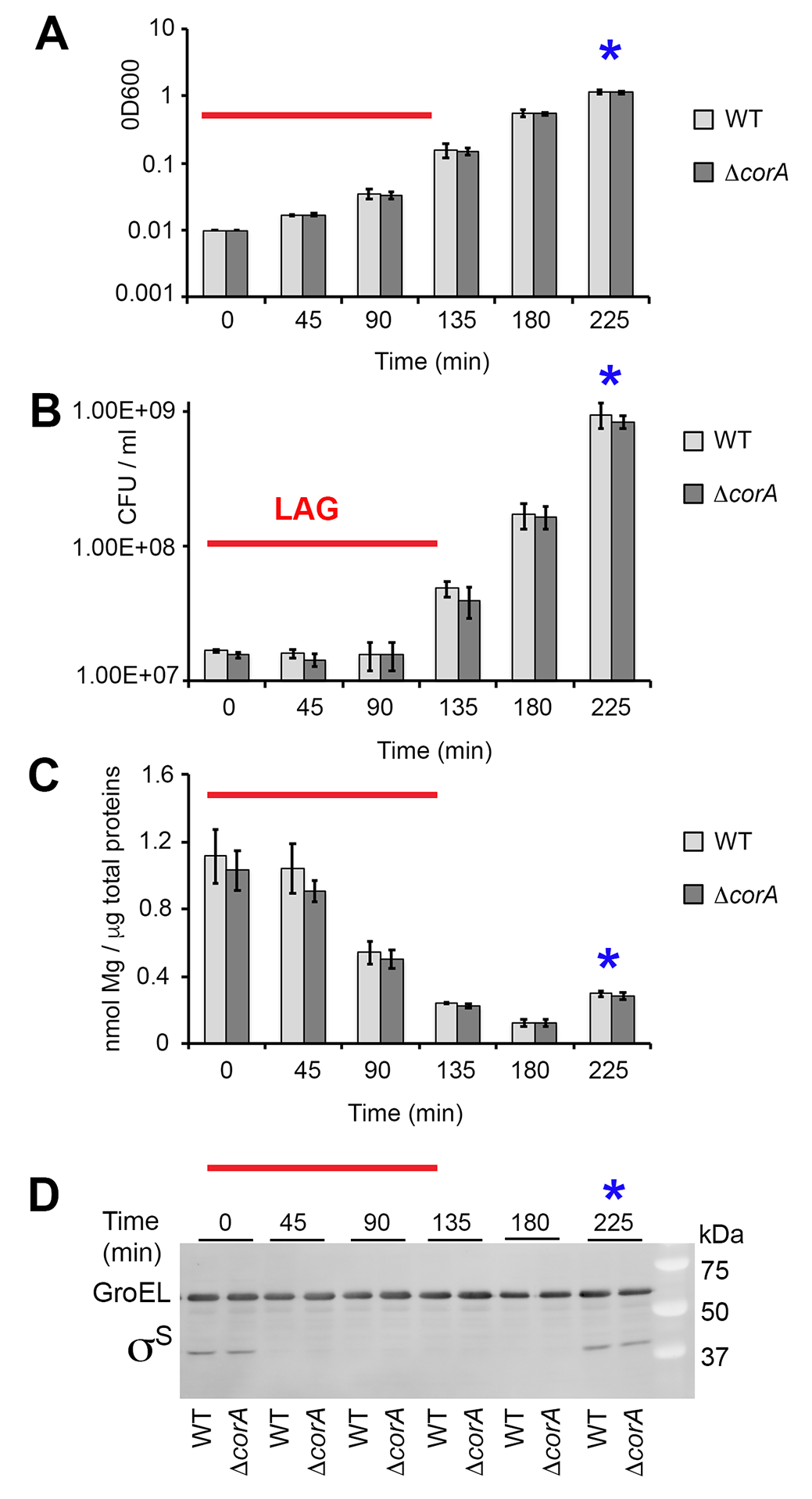

Supplement: S4 Fig — Stationary phase LB cultures of the wild-type (WT) and ΔcorA strains were diluted into fresh LB medium and the cell-associated magnesium content (panel C) was measured during the lag phase of growth (LAG, red line) and until entry to stationary phase (blue star). The kinetics of growth was followed by measuring the OD600 of the culture (panel A) and the viable cells (panel B). σS was immunodetected to similar levels in wild-type and ΔcorA cells (panel D). σS was detected only in the inoculum and at the entry to stationary phase (from OD600 of about 1), as expected [1]. GroEL was immunodetected as a loading control. The error bars represent standard errors for three independent measurements. No significant difference between the wild-type strain and the ΔcorA mutant was found (A-D). (TIF) [file pone.0291736.s009.tif]

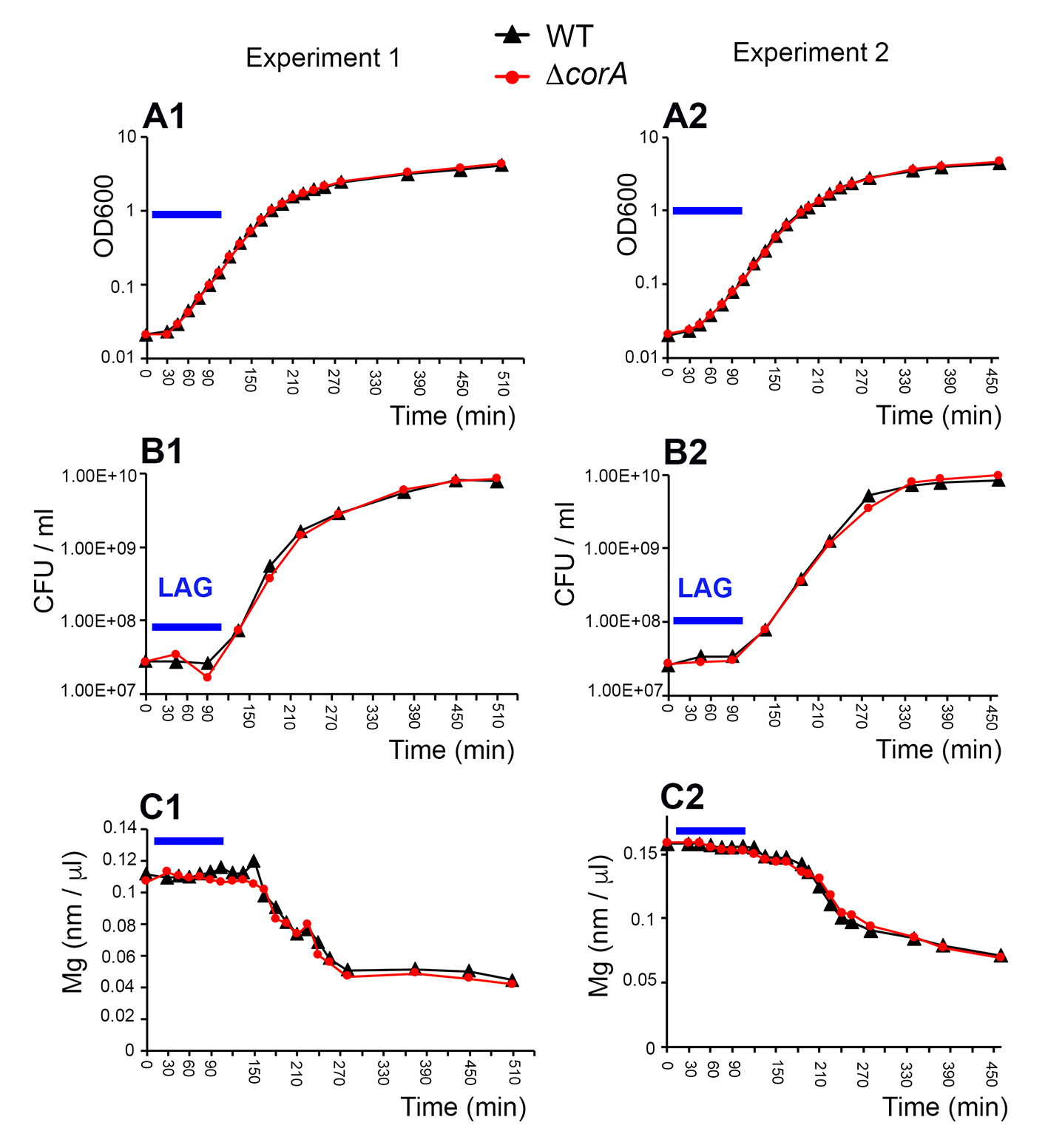

Supplement: S5 Fig — Stationary phase LB cultures of the wild-type (WT) strain and ΔcorA mutant were diluted into fresh LB medium and the extracellular magnesium content was measured during Salmonella growth (nmoles/μl, panel C). The kinetics of growth was followed by measuring the OD600 of the culture (panel A) and the viable cells (CFU/ml, panel B). Two independent experiments were conducted. Magnesium concentration in various LB batches varies from 0.1 to 0.2 mM [8] and was around 0.11 mM in Experiment 1 and 0.16 mM in Experiment 2. In both experiments, magnesium concentration was stable during the lag phase of growth (LAG, blue line). (TIF) [file pone.0291736.s010.tif]

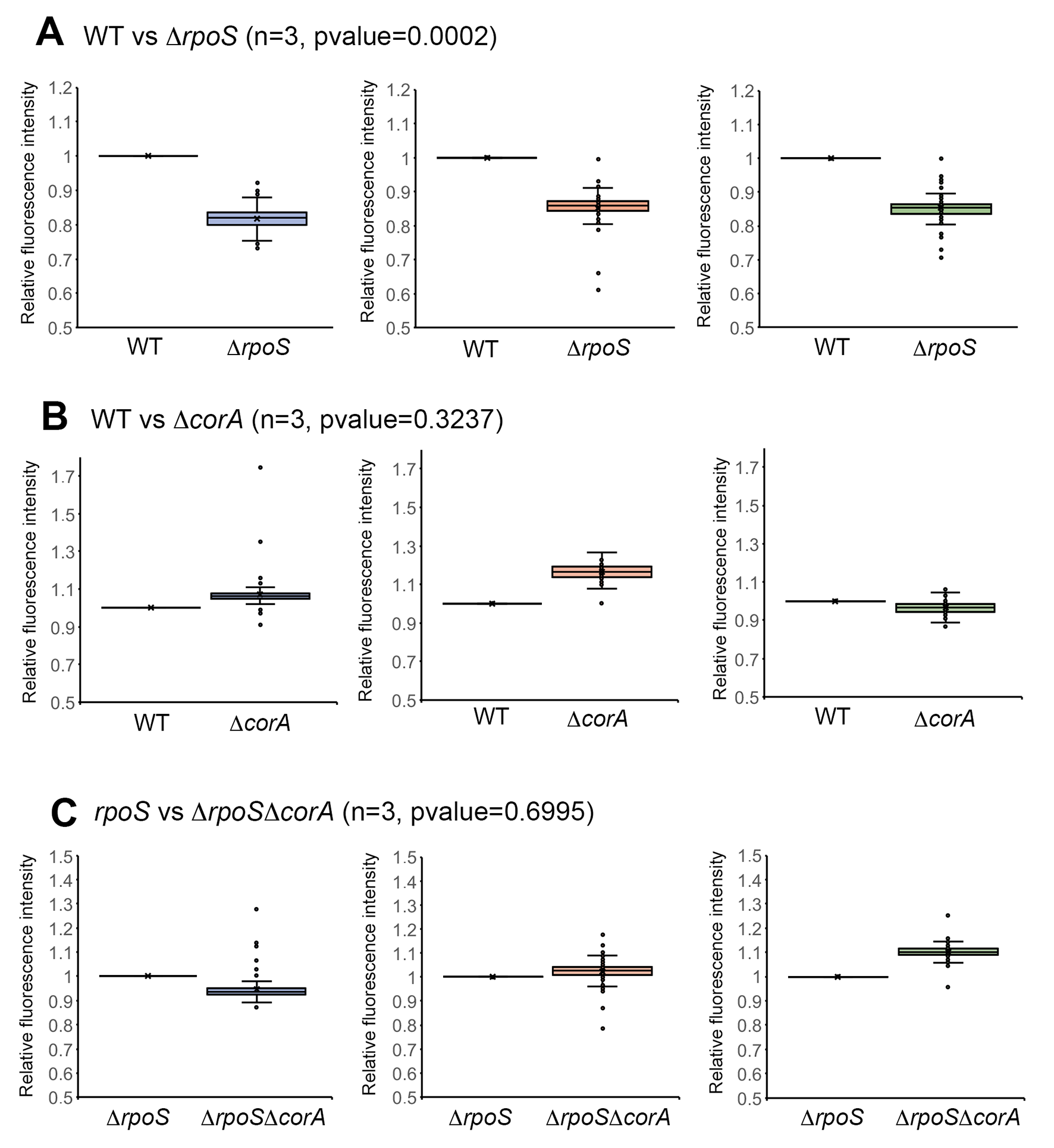

Supplement: S6 Fig — Salmonella wild-type and mutant strains were grown for 18 h at 37°C in LB. Cell-associated free magnesium amounts were measured as described (Material & Methods). The fluorescence ratio per OD600 of the bacterial culture was estimated for each strain tested using three biological replicates. Relative fluorescence values, i.e., fluorescence ratio per OD600 for a mutant relative to the fluorescence ratio per OD600 for the wild-type strain (A, B) or the ΔrpoS mutant (C) evaluated within the same experiment, were used for comparison between strains through independent experiments. For each strain comparison (A-C) the three independent experiments performed are shown with different colors and p-values are indicated. In these experiments, the only significant difference was between the wild-type strain and the ΔrpoS mutant (panel A). (TIF) [file pone.0291736.s011.tif]

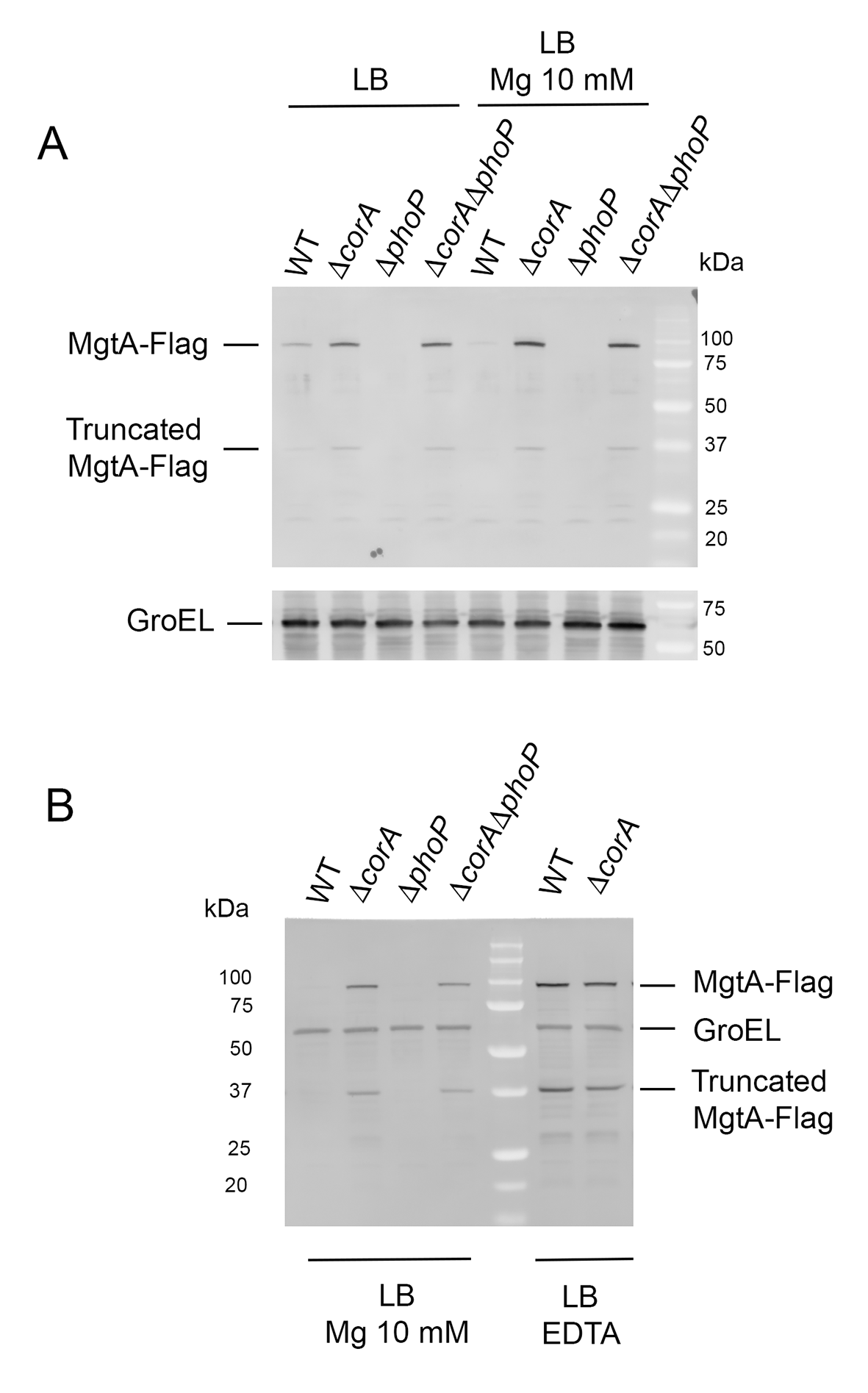

Supplement: S7 Fig — The MgtA-Flag protein was immunodetected in Salmonella wild-type (WT) and mutant strains grown for 18 h at 37°C in LB supplemented or not with MgCl2 10 mM (A, B) or EDTA 2 mM (B). MgtA is a 95-kDa protein. In most immunodetection experiments, two MgtA-Flag products were found (a full-length product at about 98-kDa and a smaller product of about 38-kDa), suggesting that the MgtA-flag protein is sensitive to degradation. Membranes used to reveal the Flag-tagged proteins with the anti-Flag antibody were then incubated in the presence of antibodies directed against GroEL used as a loading control of total protein amounts. (TIF) [file pone.0291736.s012.tif]

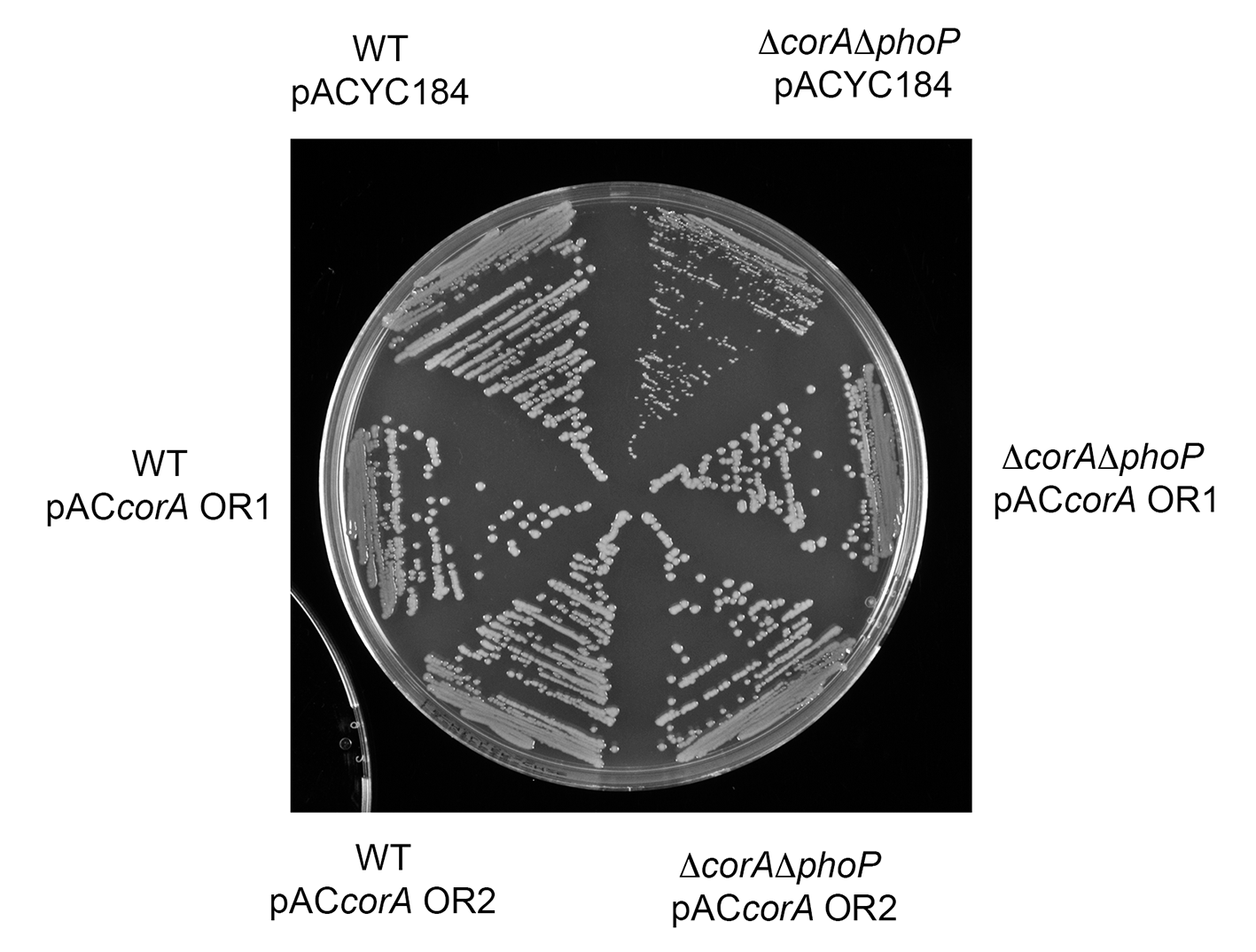

Supplement: S8 Fig — The Salmonella wild-type and ΔcorAΔphoP strains harboring the vector pACYC184 and derivatives containing the corA gene (pACcorA OR1 and OR2, S1 Table) were grown 18 h in LB at 37°C. Cultures were streaked on LB plates which were incubated at 37°C and colony size was examined overnight. A representative experiment is shown. (TIF) [file pone.0291736.s013.tif]

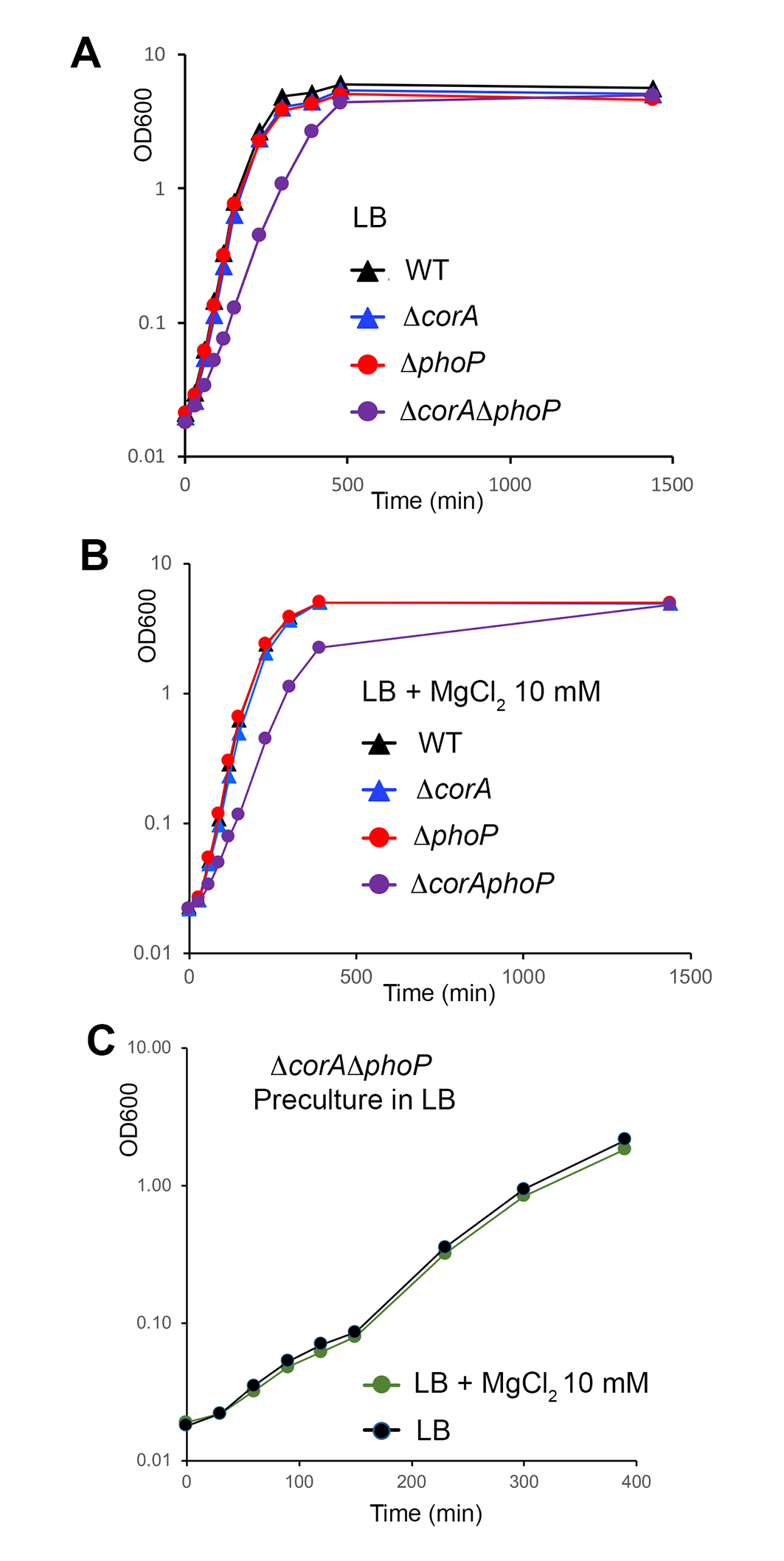

Supplement: S9 Fig — The Salmonella wild-type (WT), ΔcorA, ΔphoP and ΔcorAΔphoP strains were grown 18 h in LB (A, C) and in LB supplemented with magnesium 10 mM (B, C) at 37°C. Cultures were inoculated at the same OD600 into fresh LB supplemented or not with magnesium, as indicated. Growth was followed by measuring the optical density at 600 nm. Representative experiments are shown. (TIF) [file pone.0291736.s014.tif]

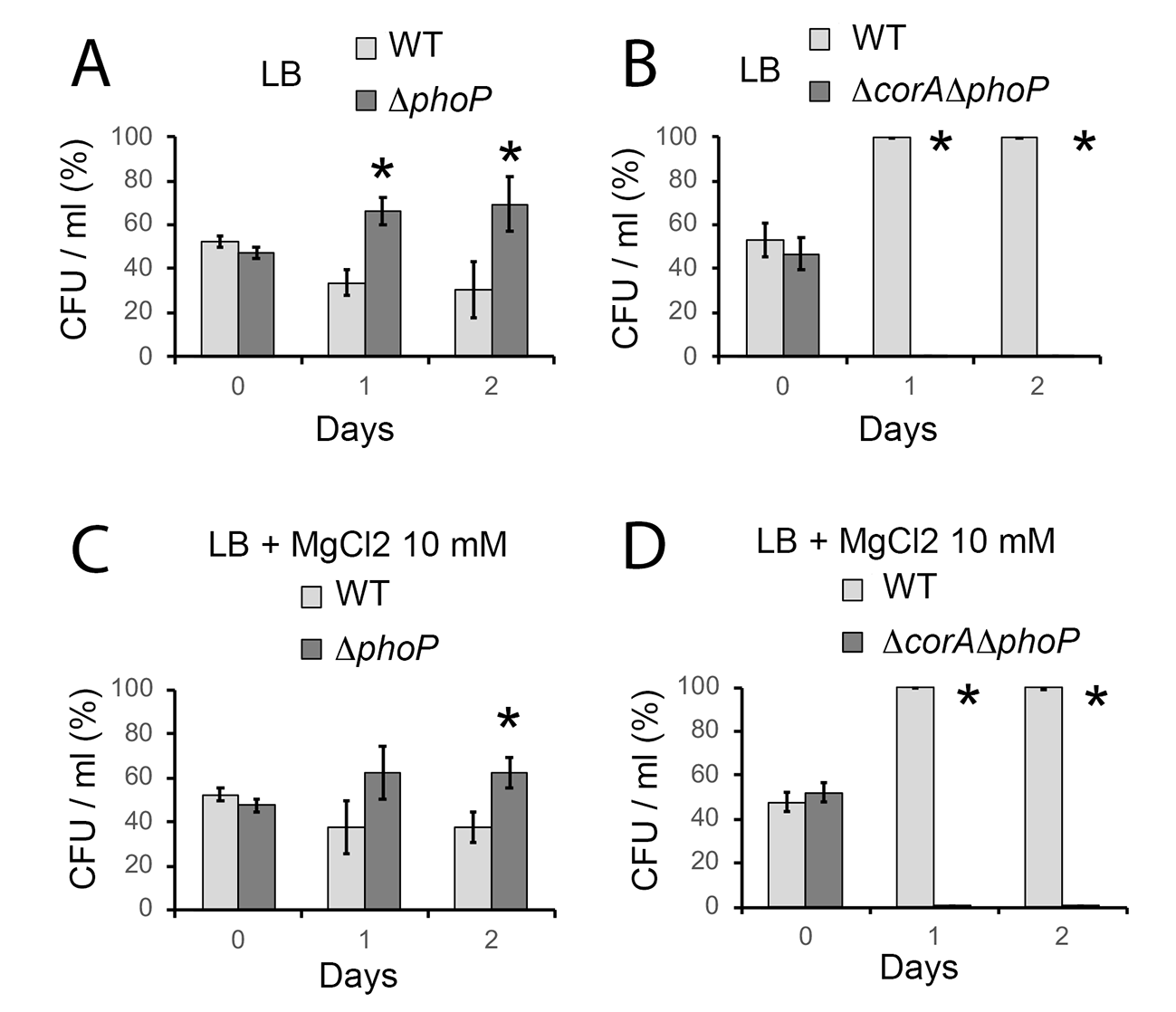

Supplement: S10 Fig — (A) Competition assays between the wild-type strain ATCC14028 (WT) and the mutants indicated were performed in LB and in LB supplemented with MgCl2 10 mM. Equal cell numbers of stationary phase cultures of the wild-type strain and the mutant strain were mixed in fresh medium to give a total of about 3000 cells ml-1 (Day 0) and the mixtures were incubated at 37°C with shaking. Aliquots of bacteria were removed at timed intervals and numbers of viable cells of each strain were determined. Cells number of each strain is reported as a percentage of the total number of viable cells in the culture. The error bars represent standard errors for three independent measurements. * Statistically significant competitive advantage or disadvantage of one strain compared to the other (p-value <0.01). (TIF) [file pone.0291736.s015.tif]

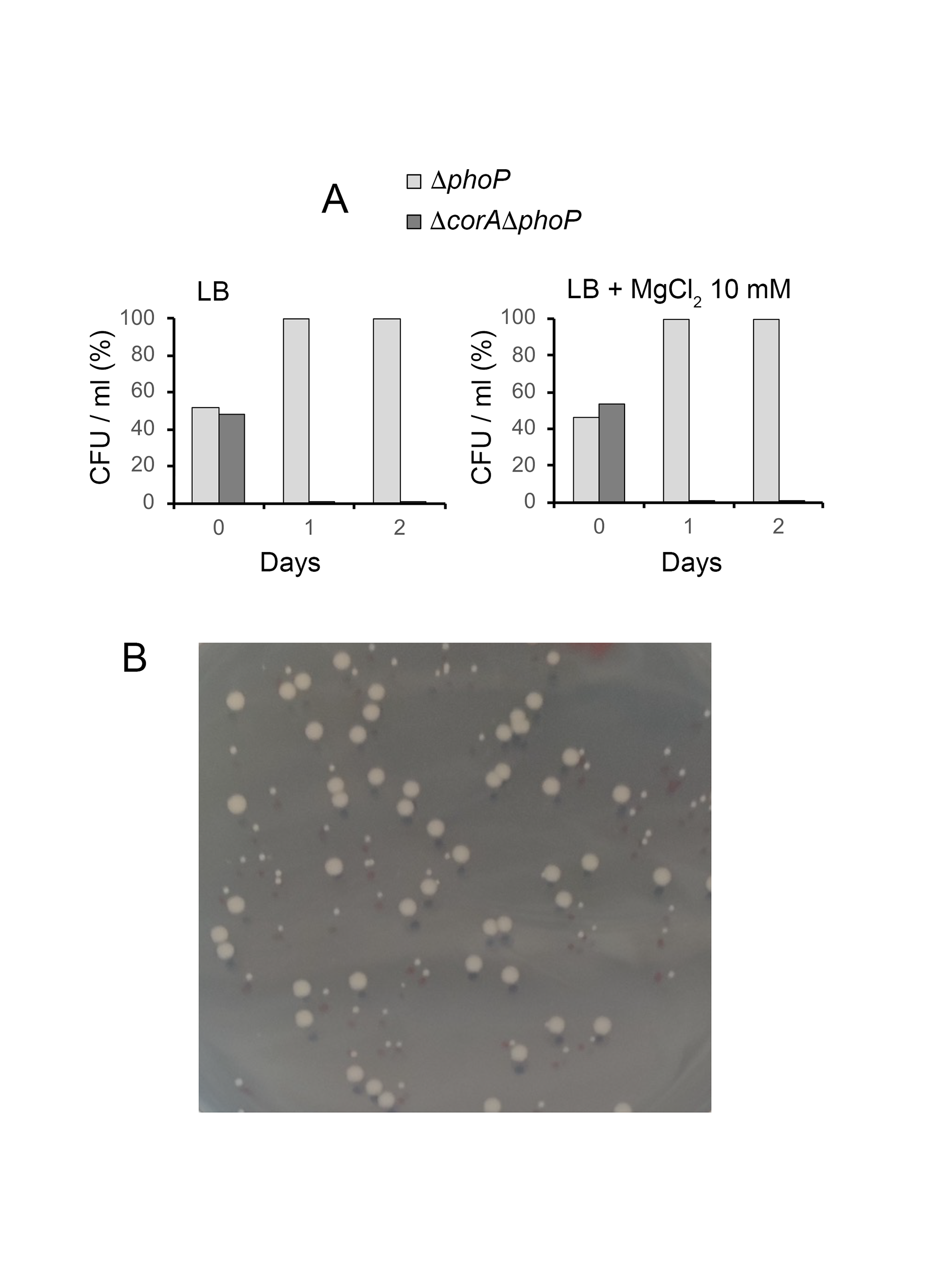

Supplement: S11 Fig — (A) Competition assays between the ΔphoP and ΔcorAF044phoP mutants were performed in LB supplemented or not with MgCl2 10 mM. Equal cell numbers of stationary phase cultures of each strain were mixed in fresh medium to give a total of about 3000 cells ml-1 (Day 0) and the mixtures were incubated at 37°C with shaking. Aliquots of bacteria were removed at timed intervals and numbers of viable cells of each strain were determined. For each time point, cells number of each strain is reported as a percentage of the total number of viable cells in the culture. (B) Mixture of the ΔphoP and ΔcorAF044phoP cells at day 0 was spread on LB plates and incubated overnight at 37°C to visualize colony size. (TIF) [file pone.0291736.s016.tif]

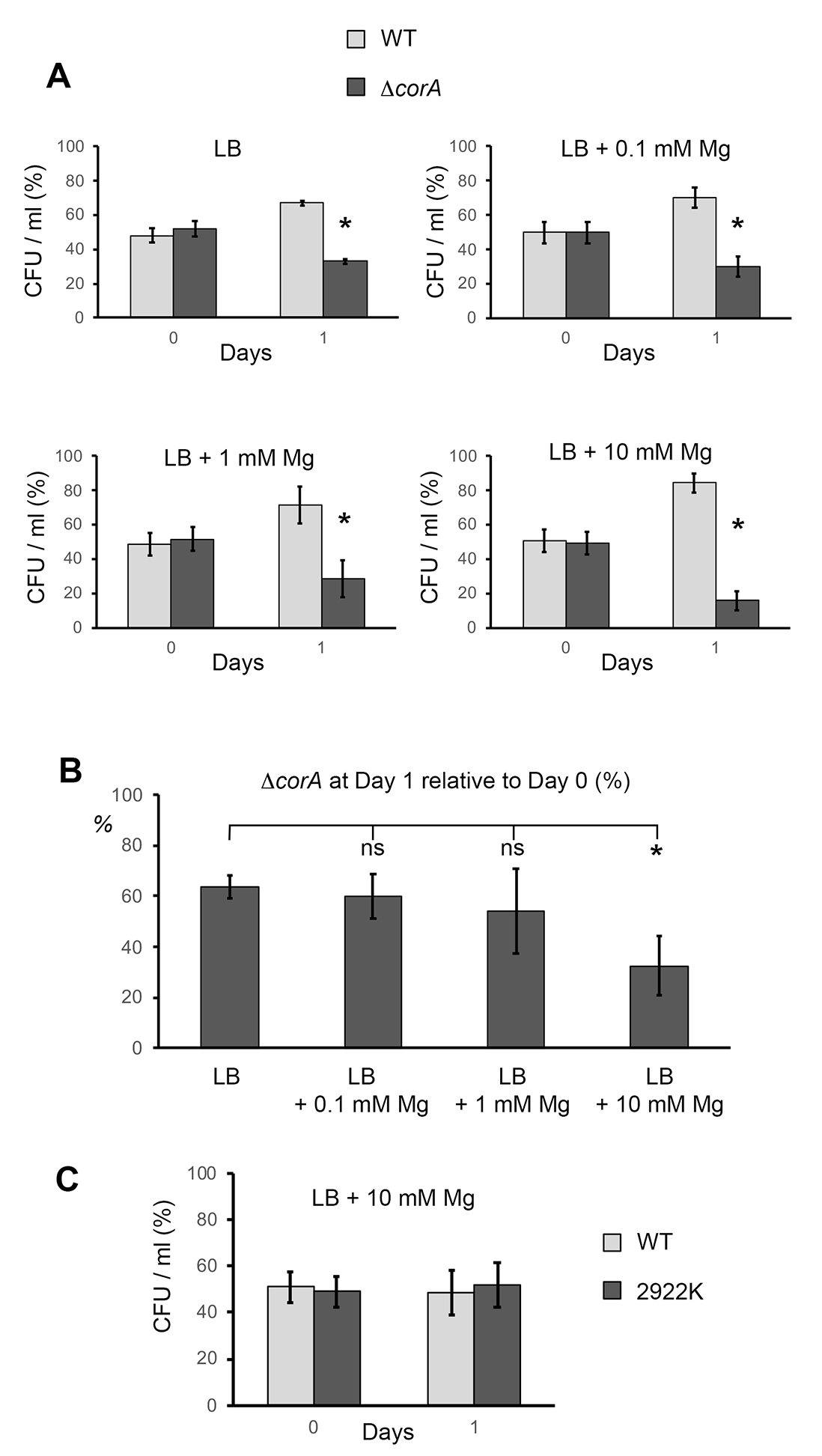

Supplement: S12 Fig — (A) Competition assays between the wild-type strain ATCC14028 (WT) and the ΔcorA mutant were performed in LB supplemented or not with MgCl2 at the indicated concentrations and at 37°C. Equal cell numbers of stationary phase cultures of the wild-type strain and the mutant were mixed in fresh medium to give a total of about 3000 cells ml-1 (Day 0) and the mixtures were incubated at 37°C with shaking. Aliquots of bacteria were removed at Day 0 and Day 1 (after 24 h of growth) and numbers of viable cells of each strain were determined. Cells number of each strain is reported as a percentage of the total number of viable cells in the culture. The error bars represent standard errors for three independent quantifications from biological replicates. * Statistically significant competitive disadvantage of the ΔcorA mutant compared to the wild-type (p-value <0.01). (B) Proportion of the ΔcorA mutant at day 1 relative to day 0 in the different growth conditions. No effect of magnesium was observed at 0.1 mM or 1 mM. The competitive disadvantage of the ΔcorA mutant was aggravated when LB was supplemented with magnesium at 10 mM (* p-value <0.01, n = 3). Under the same growth condition, the fitness of the control strain 2922K and the wild-type strain ATCC14028 was similar (C, n = 3). (TIF) [file pone.0291736.s017.tif]

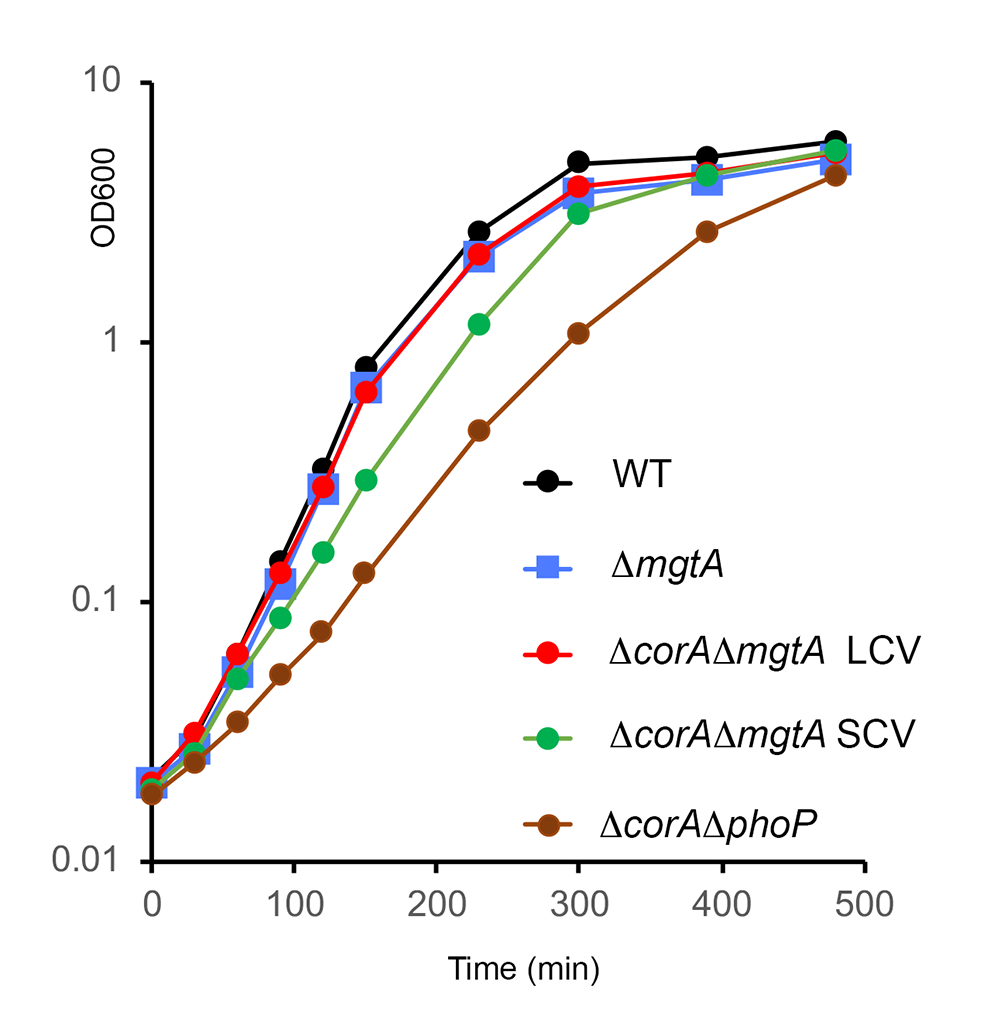

Supplement: S13 Fig — The Salmonella wild-type strain, the ΔmgtA mutant and the ΔcorAΔmgtA large colony and small colony variants (LCV and SCV, respectively) were grown 18 h in LB at 37°C. The ΔcorAΔphoP was included as a control (S9 Fig). Cultures were inoculated at the same OD600 into fresh LB and growth was followed by measuring the optical density at 600 nm. Representative experiments are shown. (TIF) [file pone.0291736.s018.tif]

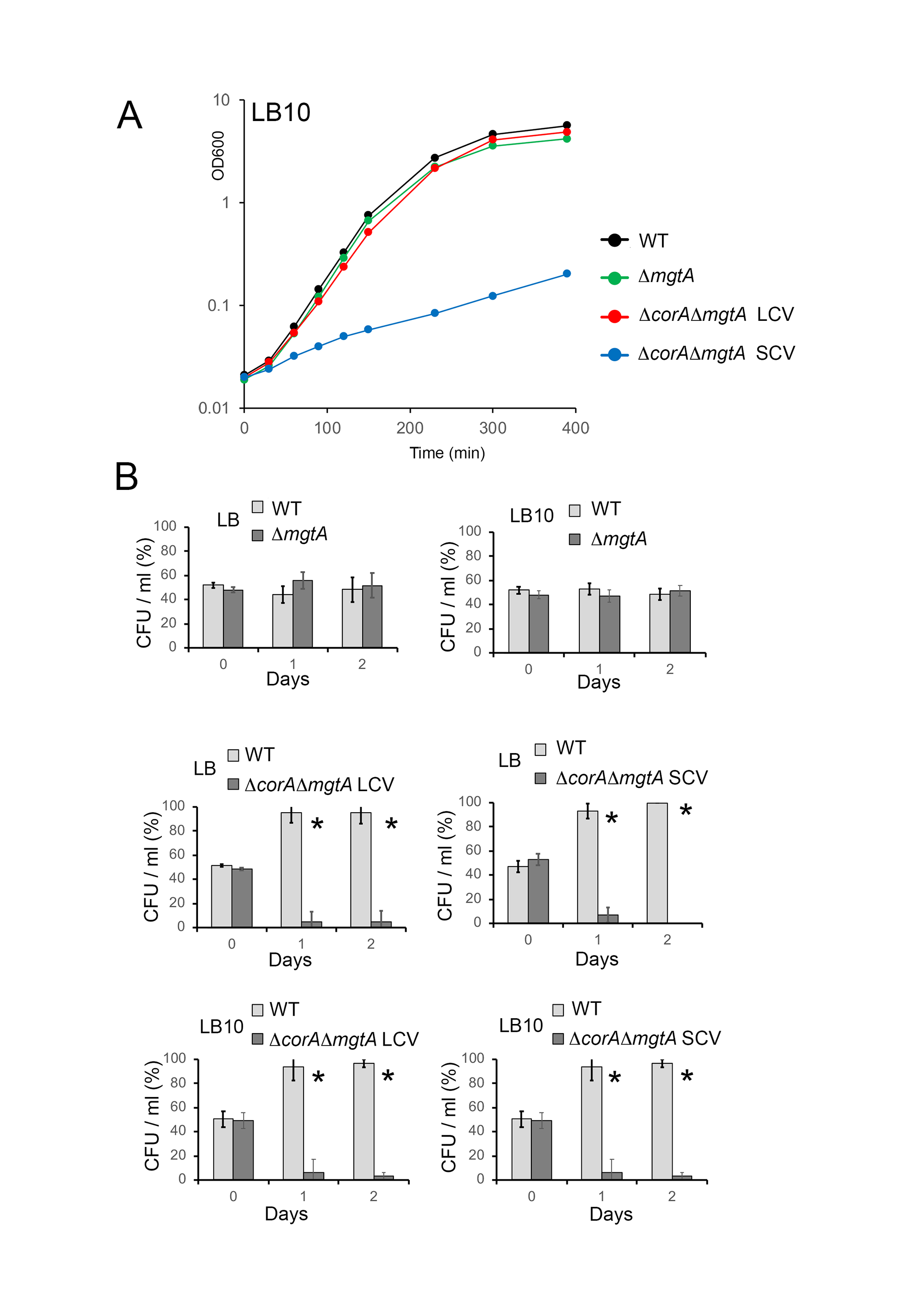

Supplement: S14 Fig — A) The Salmonella wild-type (WT), ΔmgtA and ΔcorAΔmgtA strains were grown 18 h at 37°C in LB supplemented with magnesium 10 mM. Cultures were inoculated at the same OD600 into fresh LB supplemented with magnesium and growth was followed by measuring the optical density at 600 nm. Representative experiments are shown. B) Competition assays between the wild-type strain ATCC14028 (WT) and the ΔmgtA strain or the ΔcorAΔmgtA mutants were performed in LB supplemented or not with MgCl2 10 mM at 37°C. Equal cell numbers of stationary phase cultures of the wild-type strain and the mutant were mixed in fresh medium to give a total of about 3000 cells ml-1 (Day 0) and the mixtures were incubated at 37°C with shaking. Aliquots of bacteria were removed at timed intervals and numbers of viable cells of each strain were determined. Cells number of each strain is reported as a percentage of the total number of viable cells in the culture. The error bars represent standard errors for three independent quantifications from biological replicates. * Statistically significant competitive disadvantage of the ΔcorAΔmgtA mutant compared to the wild-type (p-value <0.01). (TIF) [file pone.0291736.s019.tif]

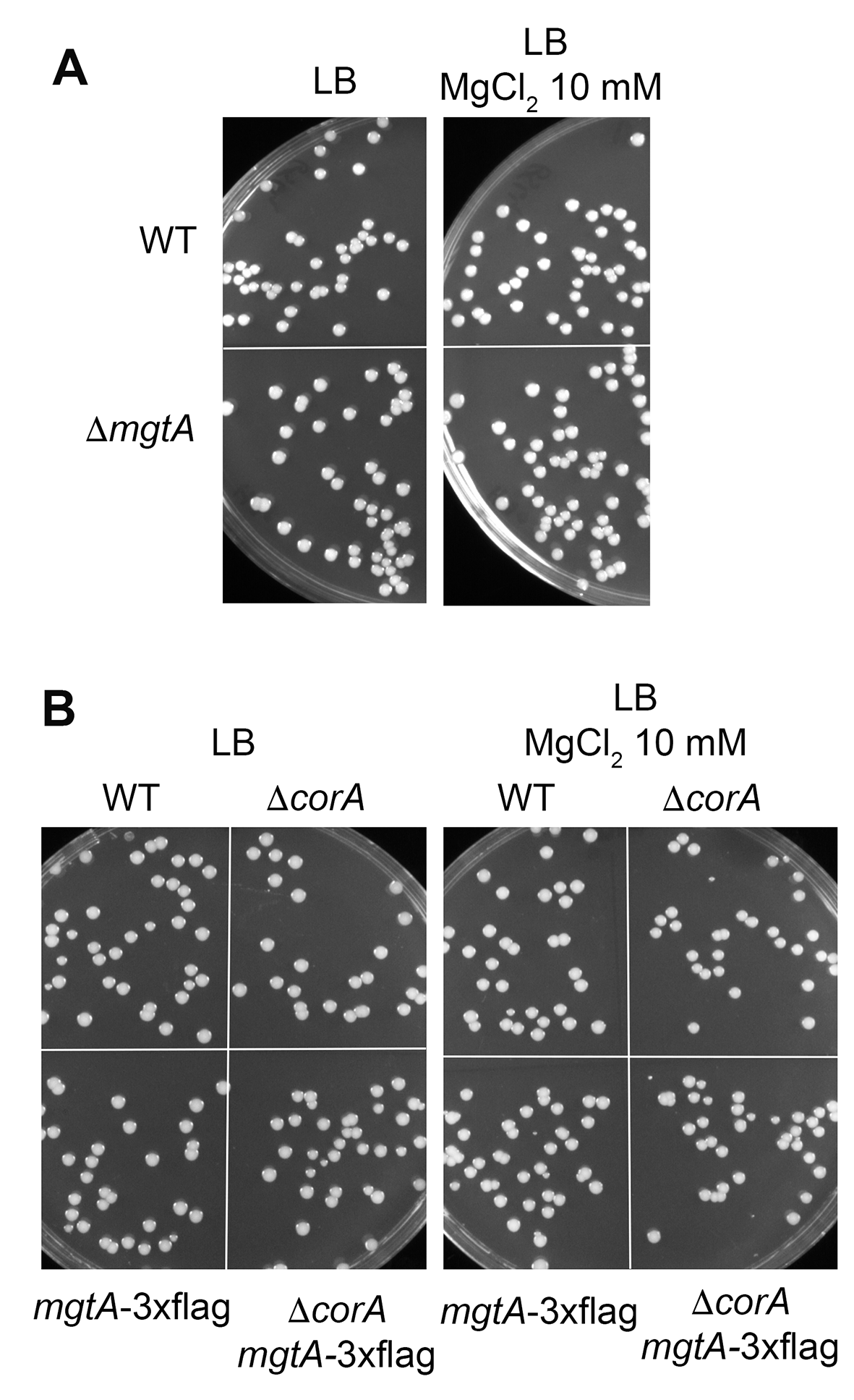

Supplement: S15 Fig — The Salmonella wild-type strain and mutants indicated were grown 18 h in LB at 37°C. Cultures were spread on LB plates which were incubated at 37°C and colony size was examined overnight. Representative experiments are shown. (TIF) [file pone.0291736.s020.tif]

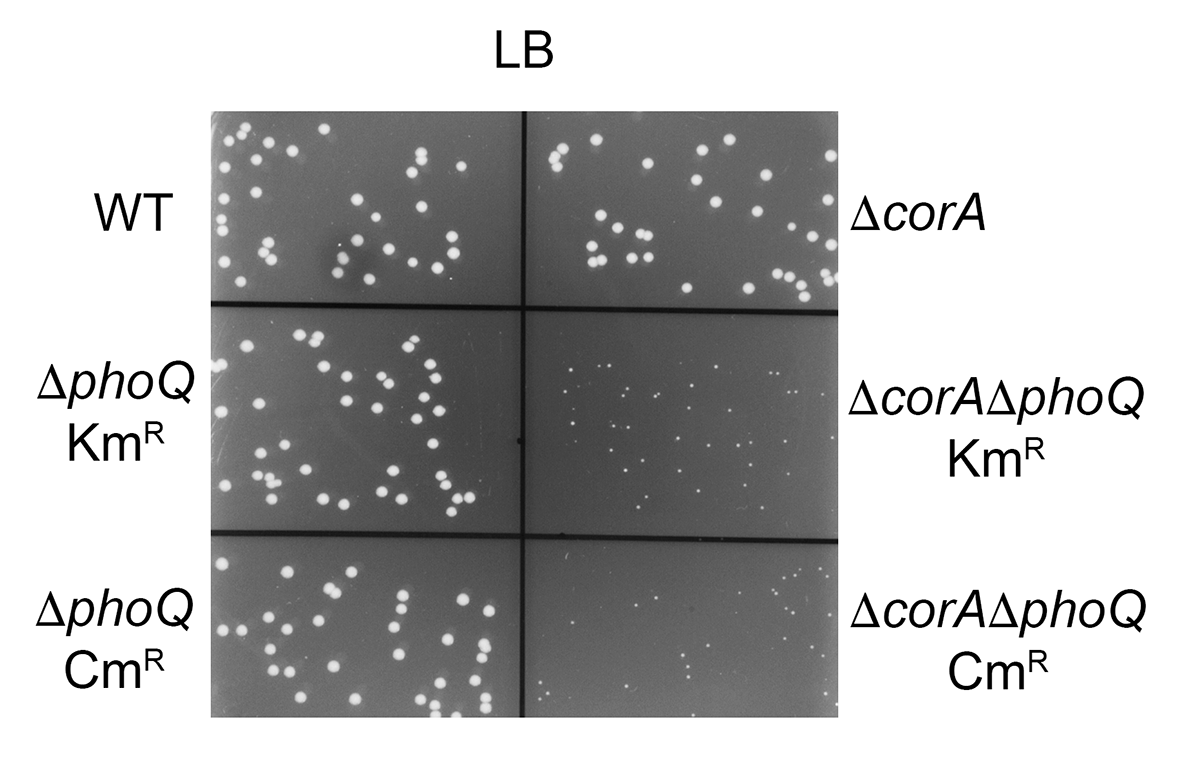

Supplement: S16 Fig — The Salmonella wild-type, ΔcorA, ΔphoQ and ΔcorAΔphoQ strains were grown 18 h in LB at 37°C. Cultures were spread on LB plates which were incubated at 37°C and colony size was examined overnight. The two ΔphoQ constructions are identical, except for the antibiotic resistance cartridge. (TIF) [file pone.0291736.s021.tif]

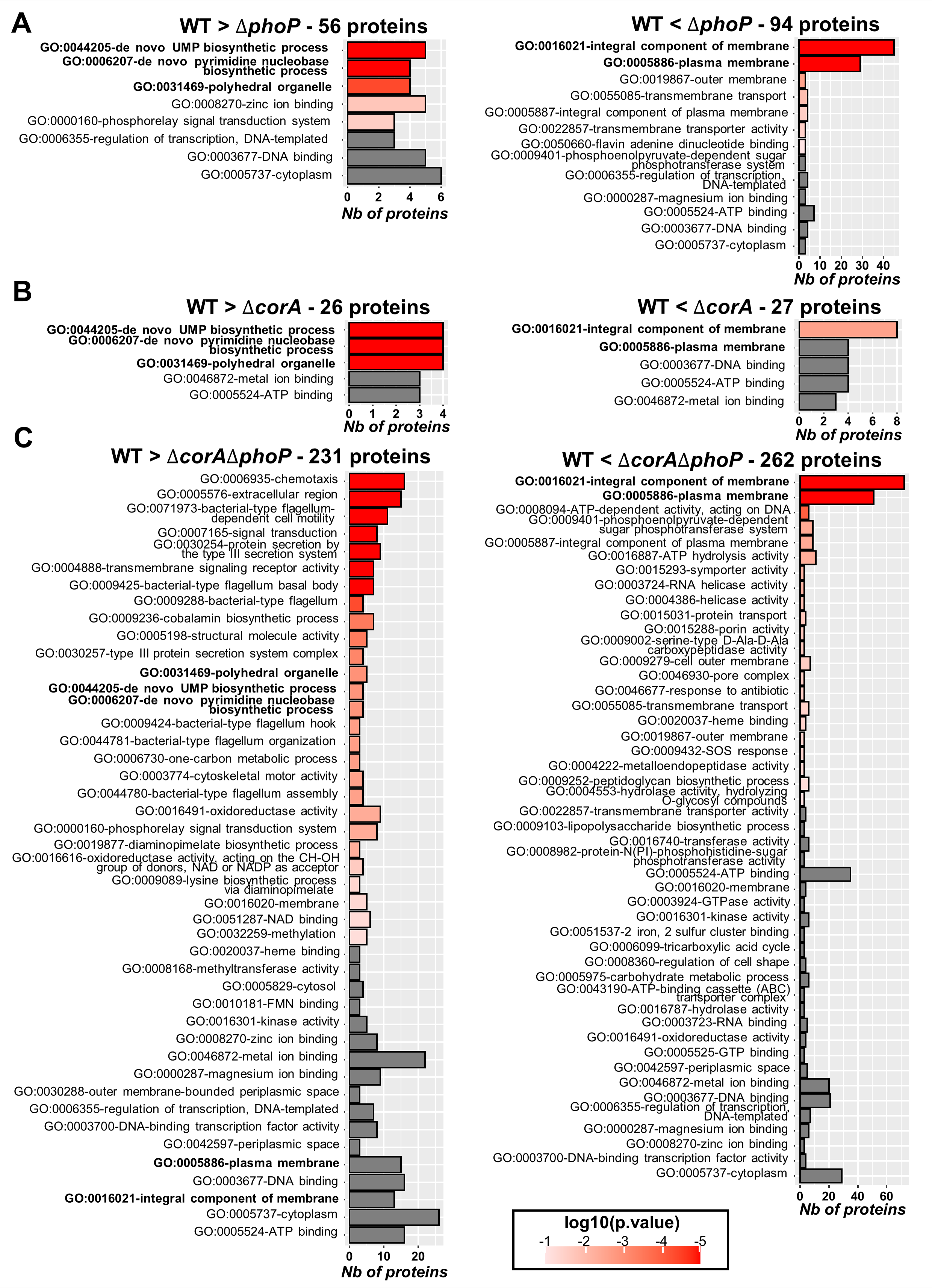

Supplement: S17 Fig — Enrichment analyses of GO terms have been performed from the lists of differentially abundant proteins between mutants and the wild-type strain (WT). (A) Enrichment analysis for the proteins significantly more abundant in WT than ΔphoP (left), and significantly more abundant in ΔphoP than WT (right). (B) Enrichment analysis for the proteins significantly more abundant in WT than ΔcorA (left), and significantly more abundant in ΔcorA than WT (right). (C) Enrichment analysis for the proteins significantly more abundant in WT than ΔcorAΔphoP (left), and significantly more abundant in ΔcorAΔphoP than WT (right). Histograms represent the number of proteins associated to a GO term in a list. Colors are function of the p-value of enrichment of the associated GO term (the redder it is, the stronger the enrichment of the term). Grey color means the p-value of enrichment is superior to 1% (e.g., the GO term is not enriched). GO terms of interest are highlighted in bold. (TIF) [file pone.0291736.s022.tif]

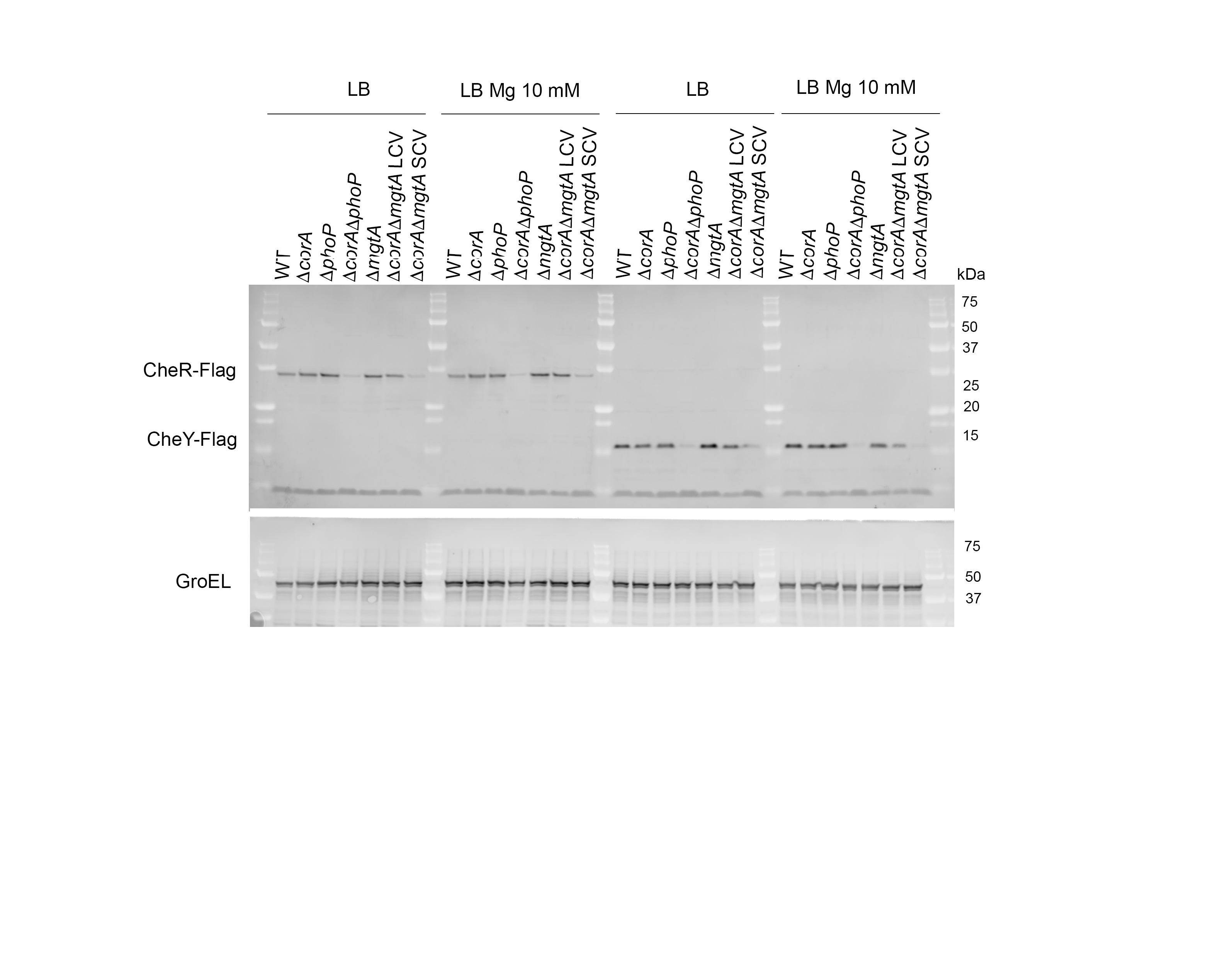

Supplement: S18 Fig — The CheR-Flag and CheY-Flag proteins were immunodetected in the Salmonella strains indicated grown for 18 h at 37°C in LB supplemented or not with MgCl2 10 mM. Membranes used to reveal the Flag-tagged proteins with the anti-Flag antibody were then incubated in the presence of antibodies directed against GroEL used as a loading control of total protein amounts. These immunodetection data are consistent with the MS-based data showing reduced abundance of the CheR and CheY proteins in the ΔcorAΔphoP mutant (Fig 6 and S1 Dataset). In addition, these data reveal a reduced abundance of the CheR and CheY proteins in the ΔcorAΔmgtA SCV mutant, compared to the ΔcorAΔmgtA LCV mutant. (TIF) [file pone.0291736.s023.tif]

**A**

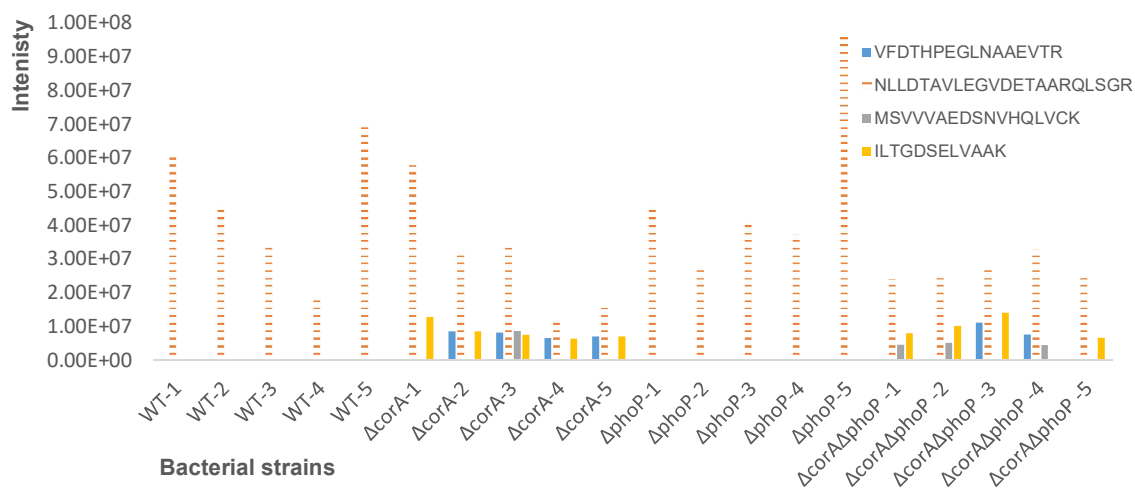

**B1**

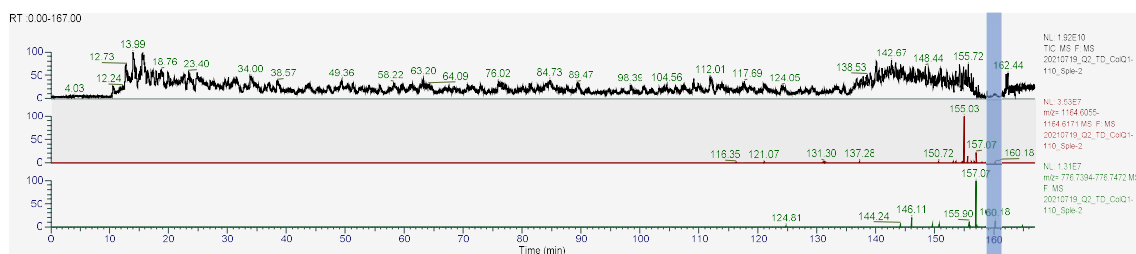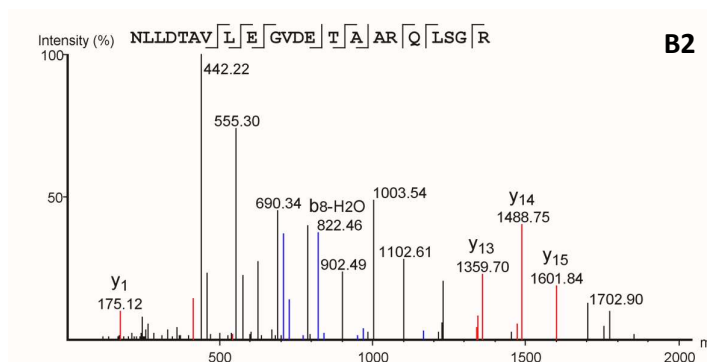

**B2**

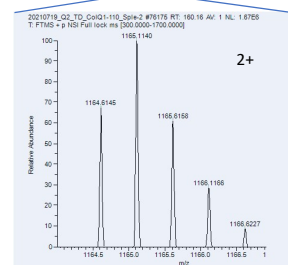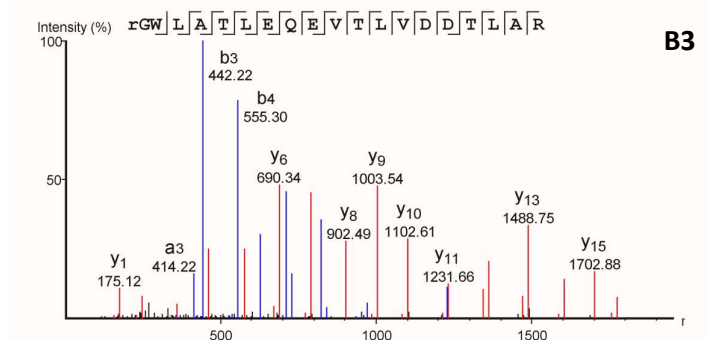

**B3**

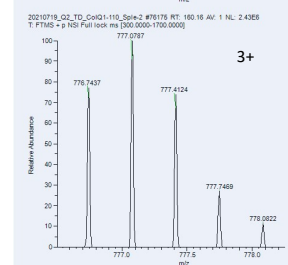

Supplement: S19 Fig — (A) From the MaxQuant analysis, histogram representing intensities of identified peptides for MgtA. (B) MS1 and MS2 spectra. B1: Chromatogram of a wild-type sample (grey box) with the extracted ion chromatogram for 2+ (1164.6113 m/z) and 3+ (776.7433 m/z) charge states of the NLLDTAVLEGVDETAARQLSGR peptide (blue box). B2 (orange box): HCD fragmentation spectra, from database search using PEAKS software, attributed to the NLLDTAVLEGVDETAARQLSGR peptide. B3 (orange box): HCD fragmentation of a similar spectra, from de novo sequencing using PEAKS software, attributed to a RGWLATLEQVTLVDDTLAR peptide. See also S1 Text. (PDF) [file pone.0291736.s024.pdf]

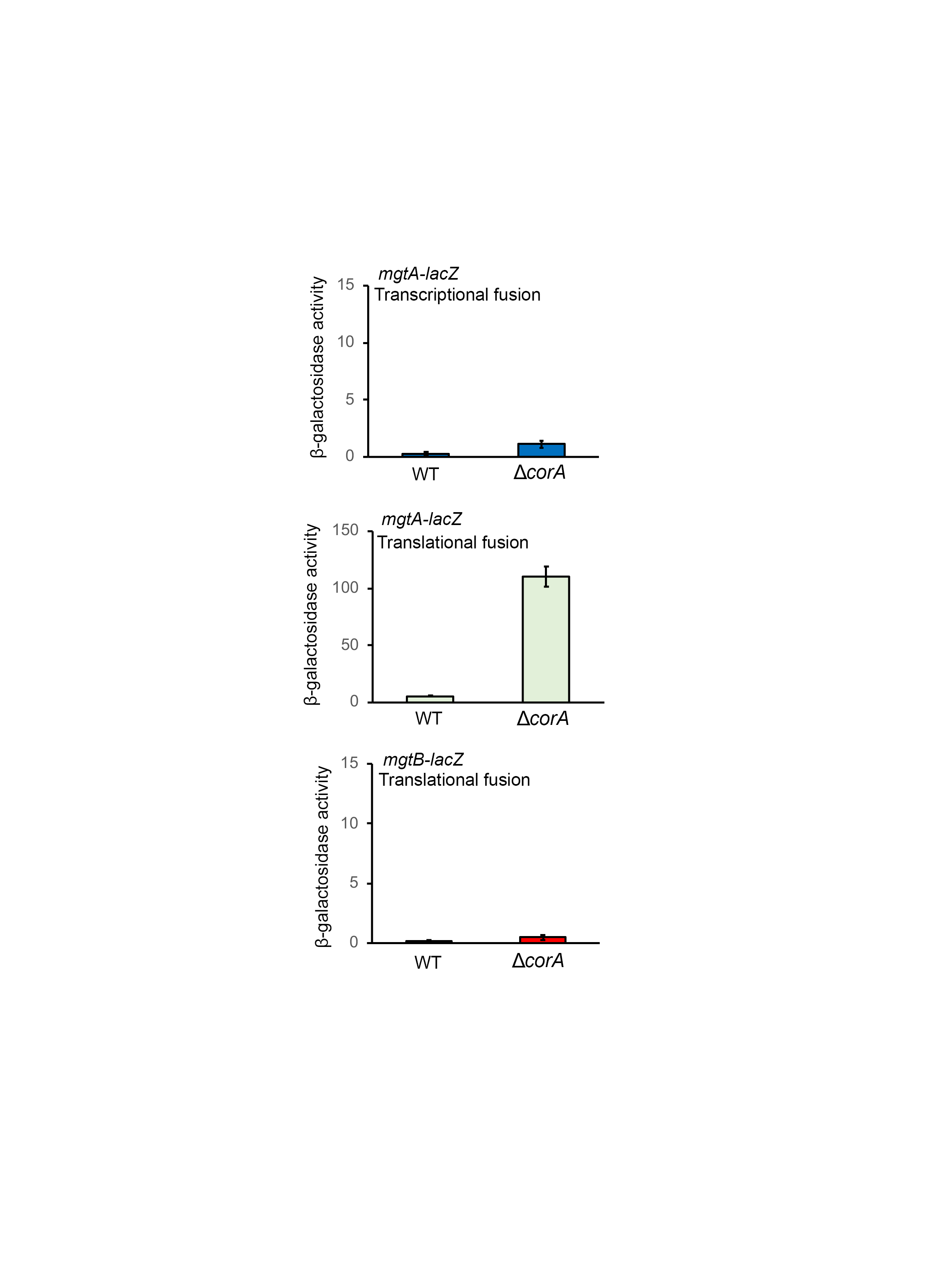

Supplement: S21 Fig — Expression of the transcriptional and translational mgtA-lacZ fusions was evaluated in the wild-type and ΔcorA strains, grown for 18 h in LB at 37°C. A translational mgtB-lacZ fusion was included as a control. Bar graphs represent the mean β-galactosidase activity, and error bars represent standard deviation of at least three independent experiments. The transcriptional and translational mgtA-lacZ fusions were both inserted at the beginning of the mgtA ORF (S2 Table). The translational mgtB-lacZ fusion was inserted at the beginning of the mgtB ORF (S2 Table). The mgtA-lacZ and mgtB-lacZ translational fusions were expressed to very low levels in the wild-type strain and expression of the mgtA-lacZ (but not mgtB-lacZ) fusion was increased in the ΔcorA mutant, in agreement with the immunodetection data (Figs 1 and S1). The strong positive effect of the ΔcorA mutation on mgtA expression was not observed with the transcriptional mgtA-lacZ fusion, suggesting a post-transcriptional effect. (TIF) [file pone.0291736.s026.tif]

# S1A Fig Raw data

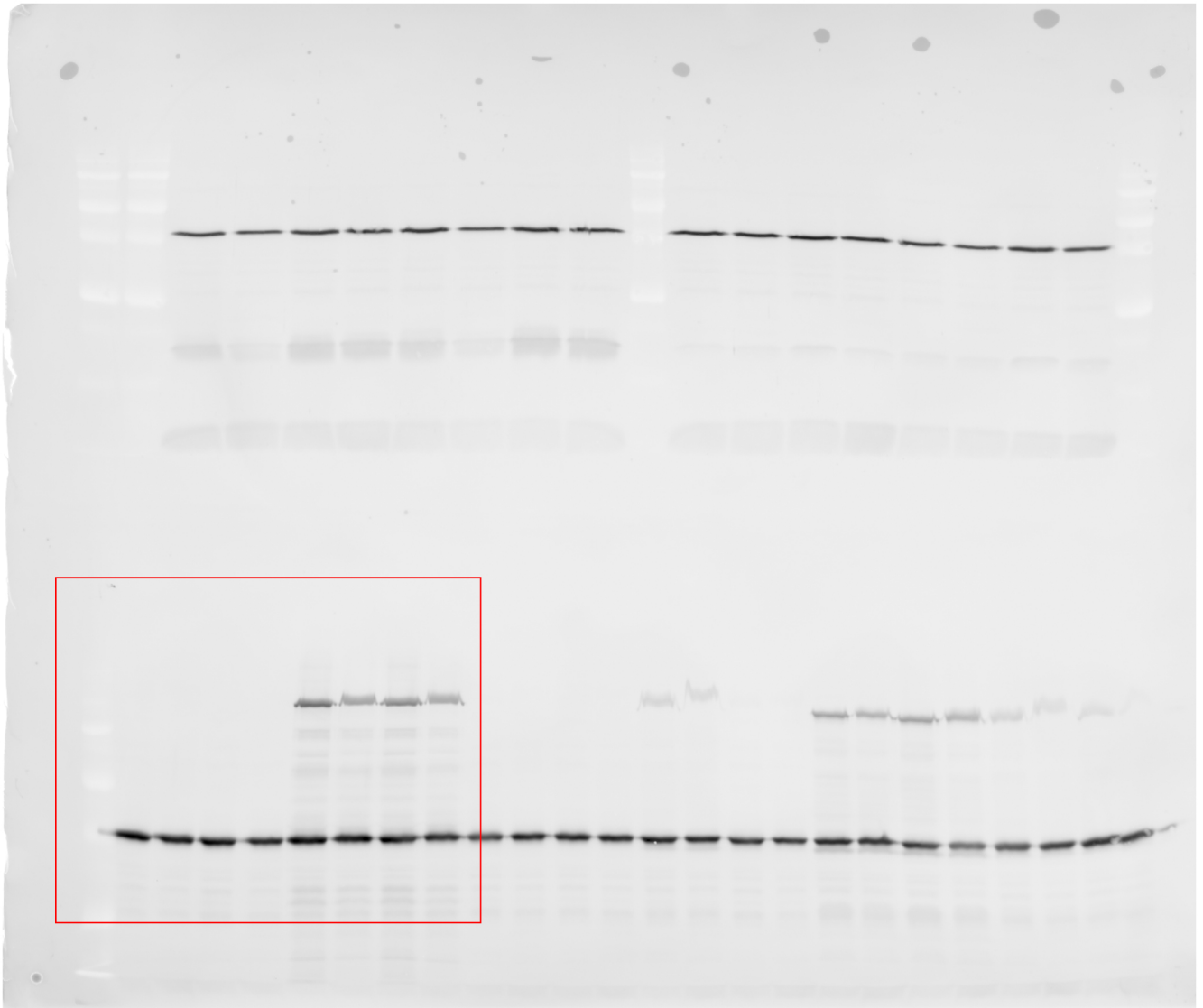

## S1B Fig Raw data

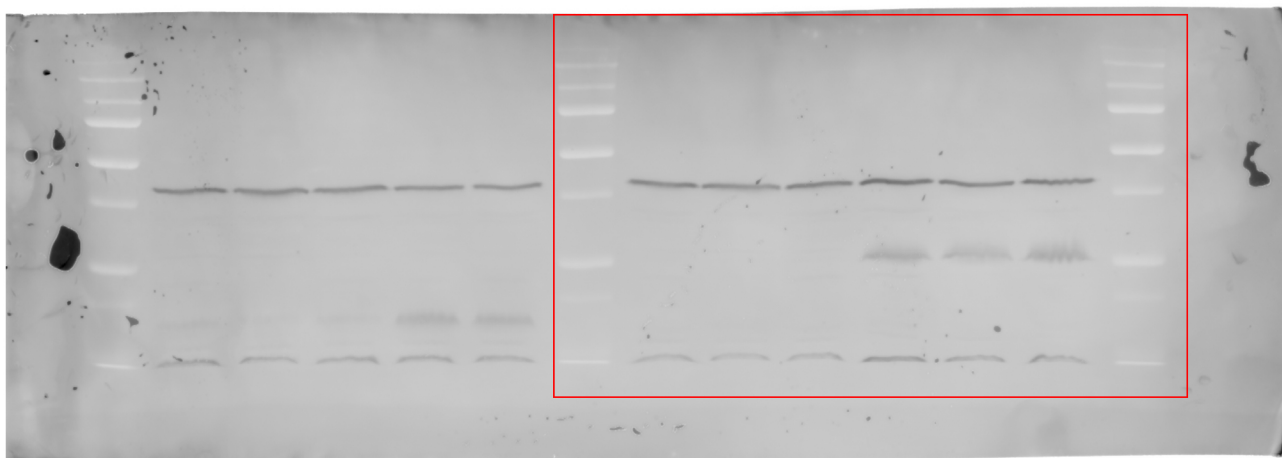

Supplement: S1 Raw images — (PDF) [file pone.0291736.s027.pdf]

## S2A Fig raw data

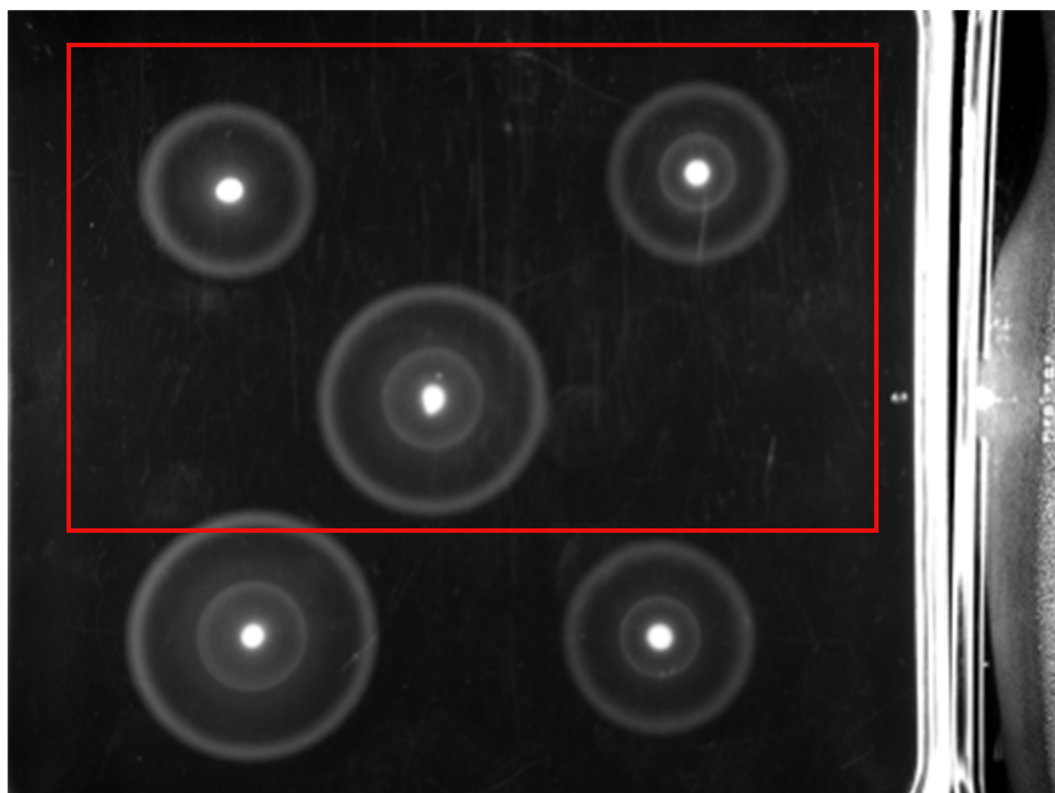

## S2B Fig raw data

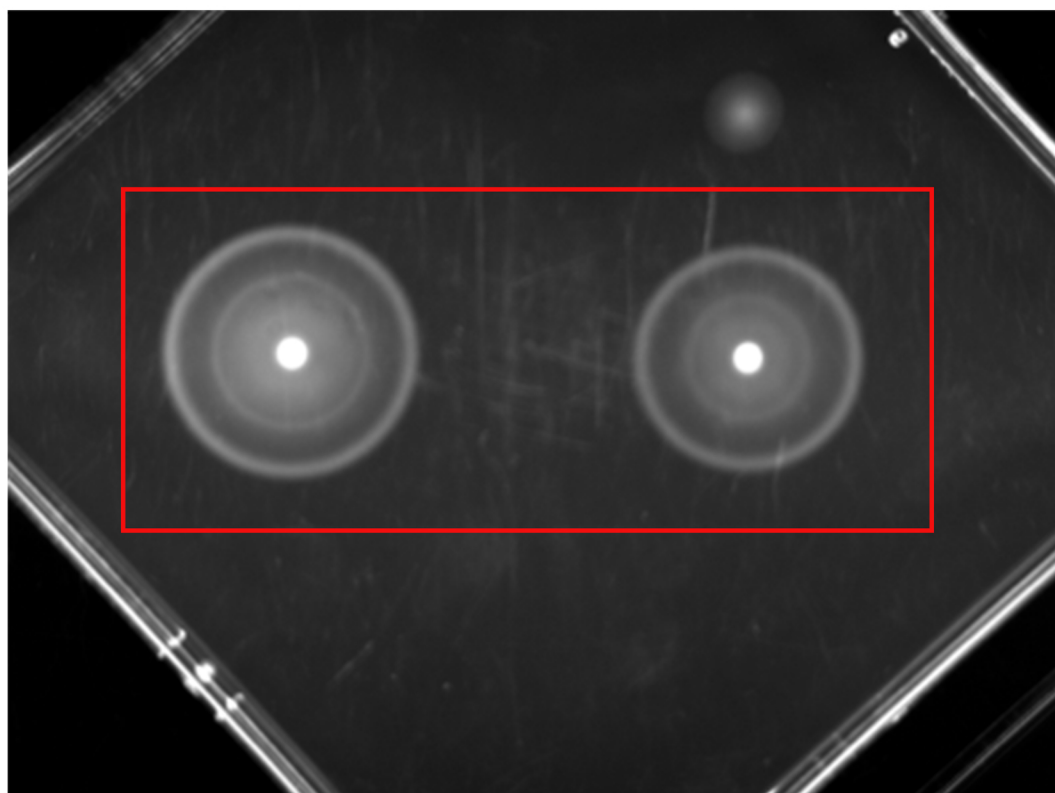

## S2C Fig raw data

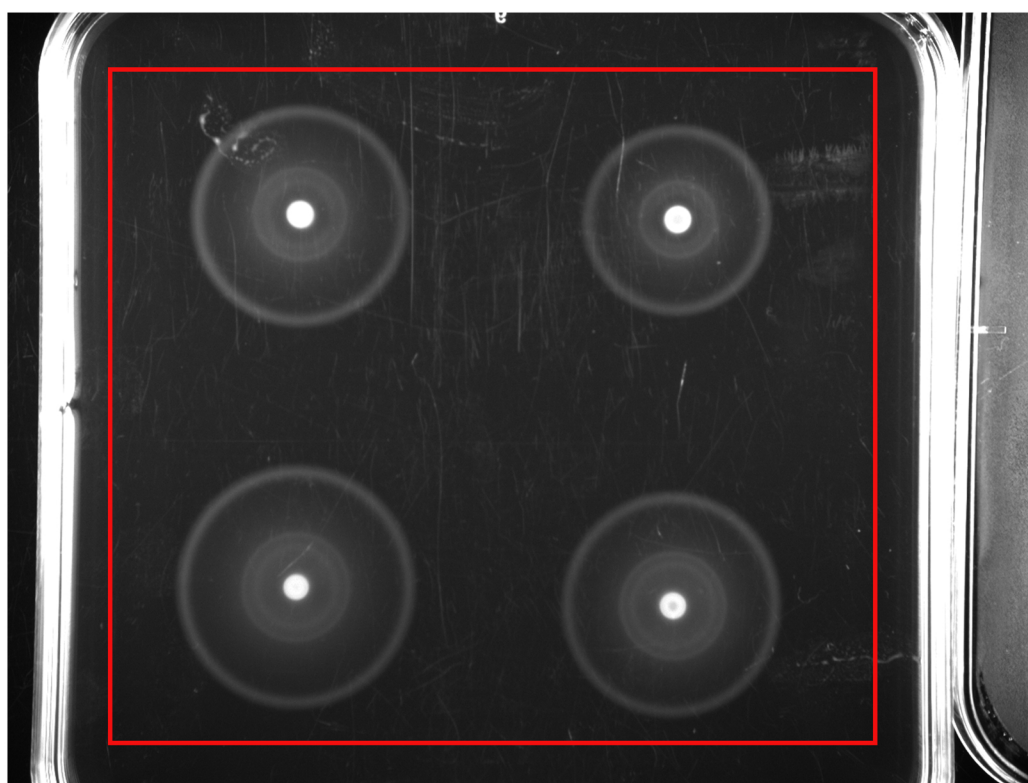

Supplement: S2 Raw images — (PDF) [file pone.0291736.s028.pdf]

## S4D Fig Raw data

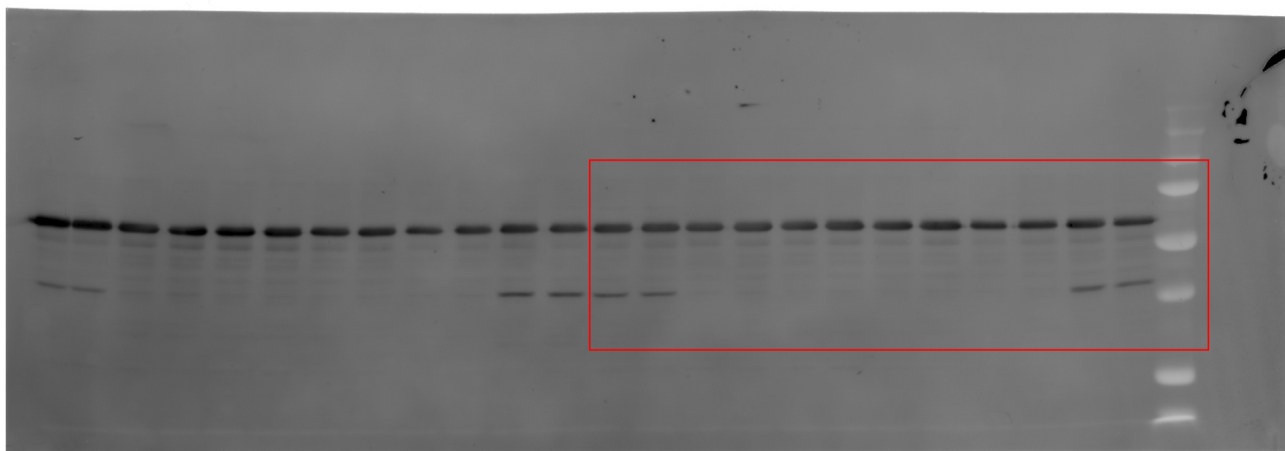

Supplement: S3 Raw images — (PDF) [file pone.0291736.s029.pdf]

S7A Fig Raw data

anti Flag

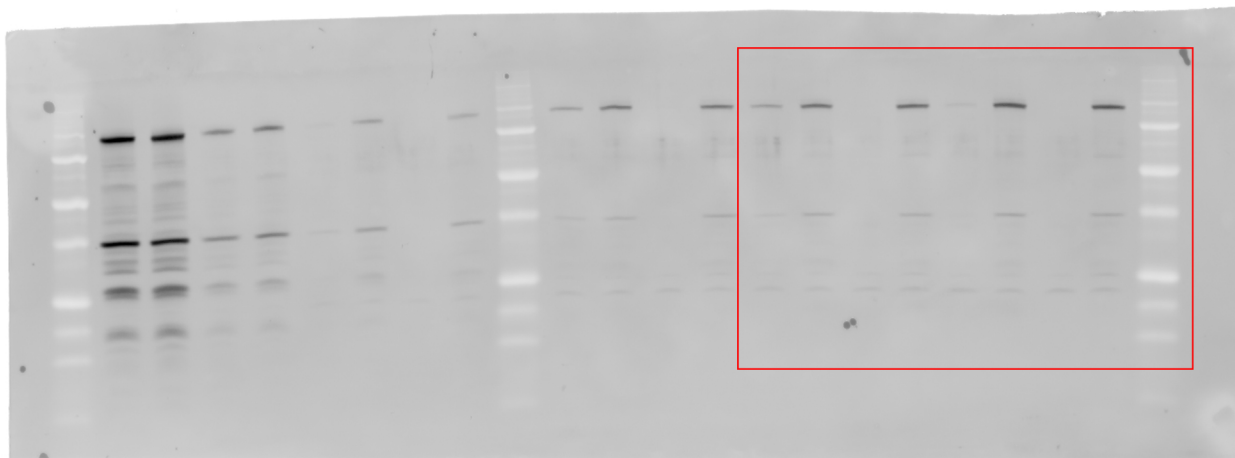

↓ anti GroEL

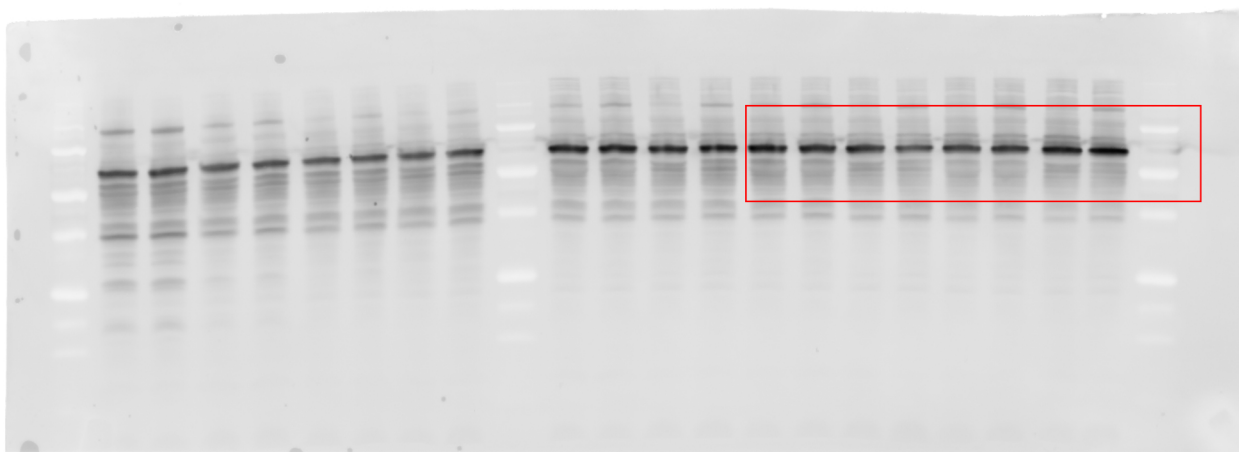

## S7B Fig Raw data

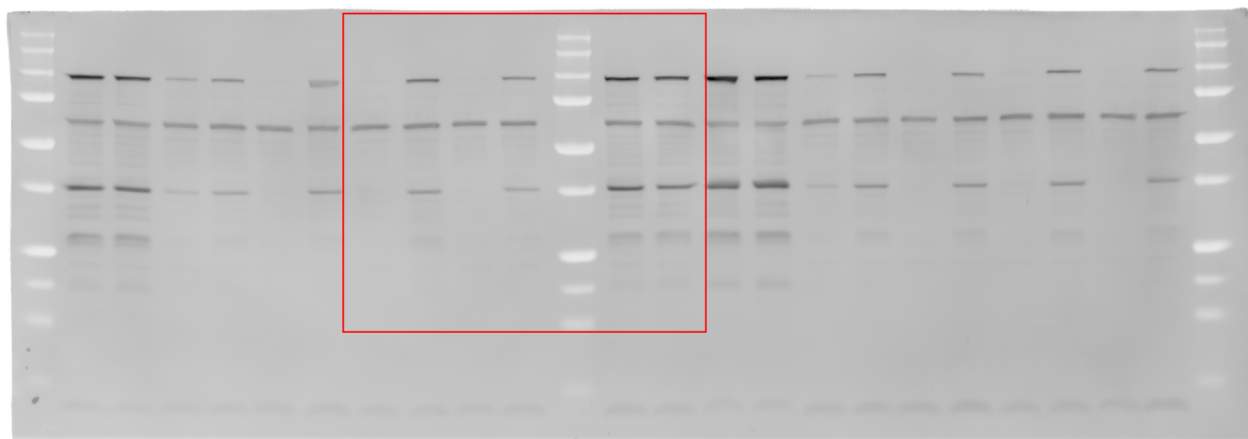

Supplement: S4 Raw images — (PDF) [file pone.0291736.s030.pdf]

S8 Fig raw image

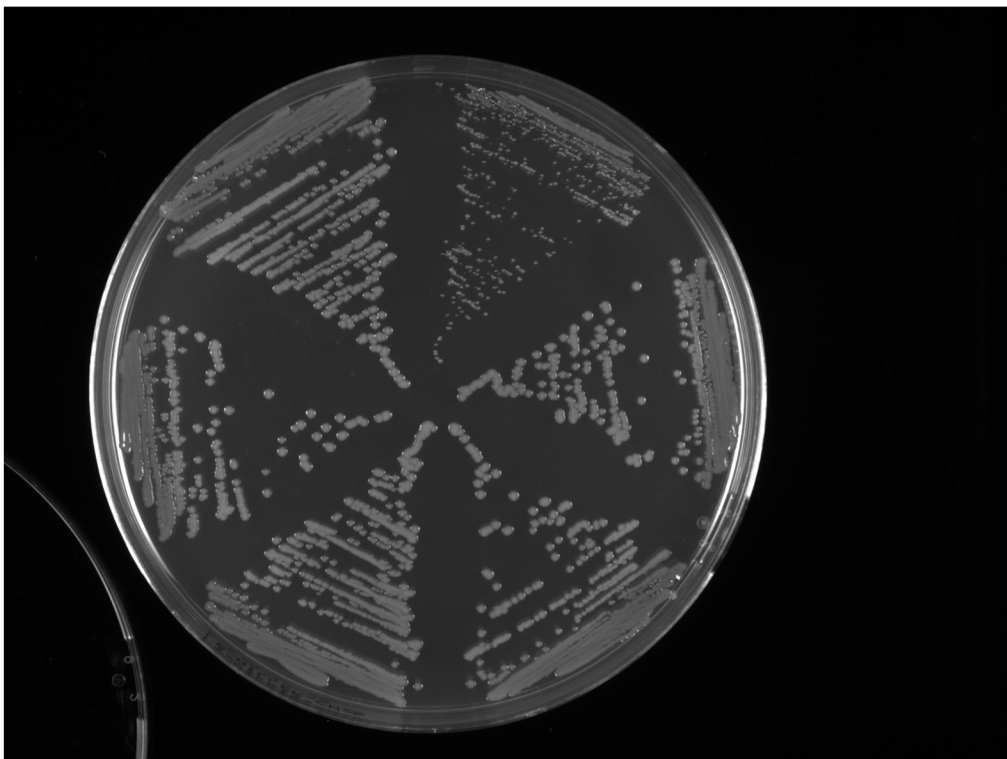

Supplement: S5 Raw images — (PDF) [file pone.0291736.s031.pdf]

S11B Fig raw data

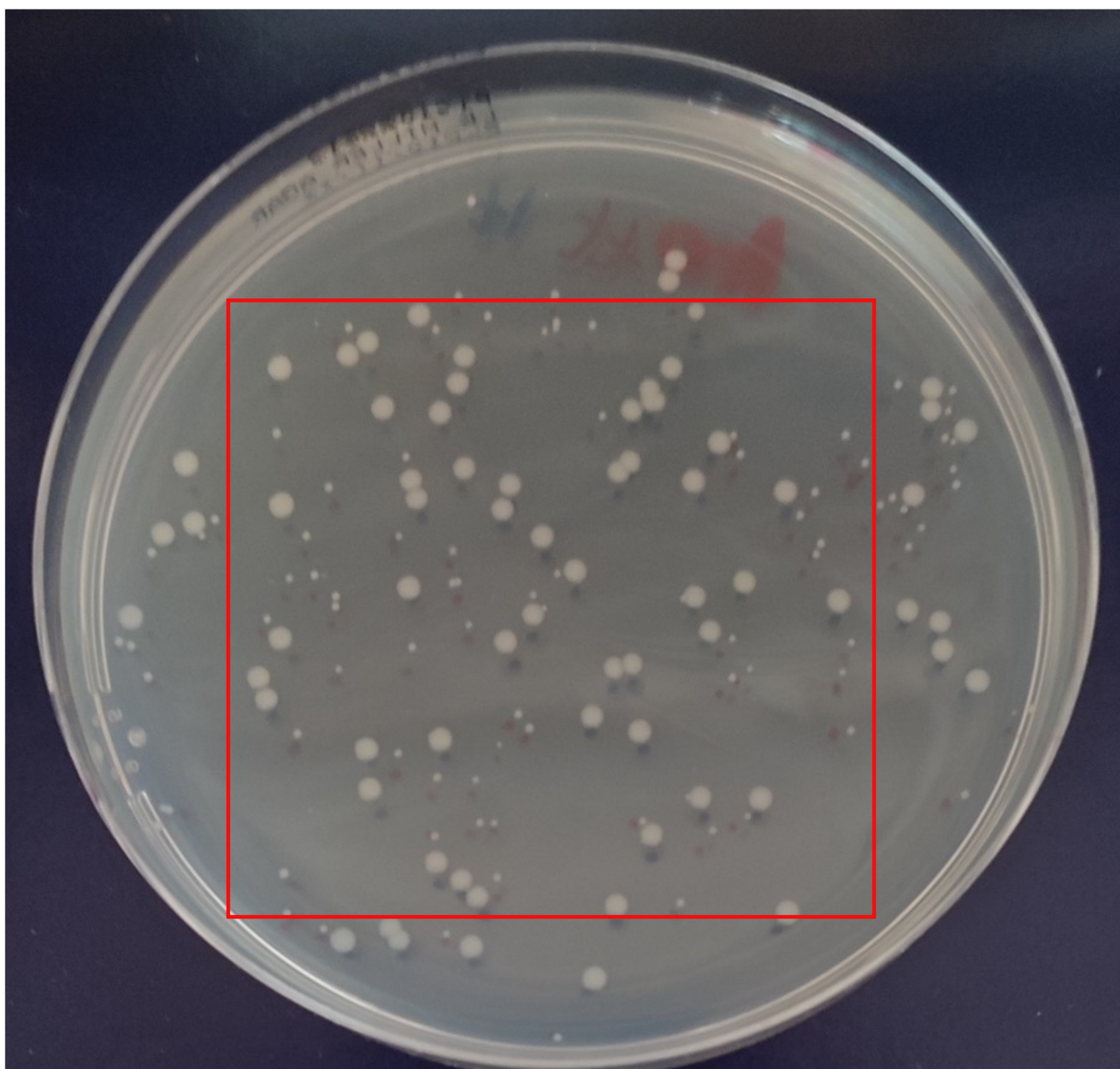

Supplement: S6 Raw images — (PDF) [file pone.0291736.s032.pdf]

S15A Fig raw image

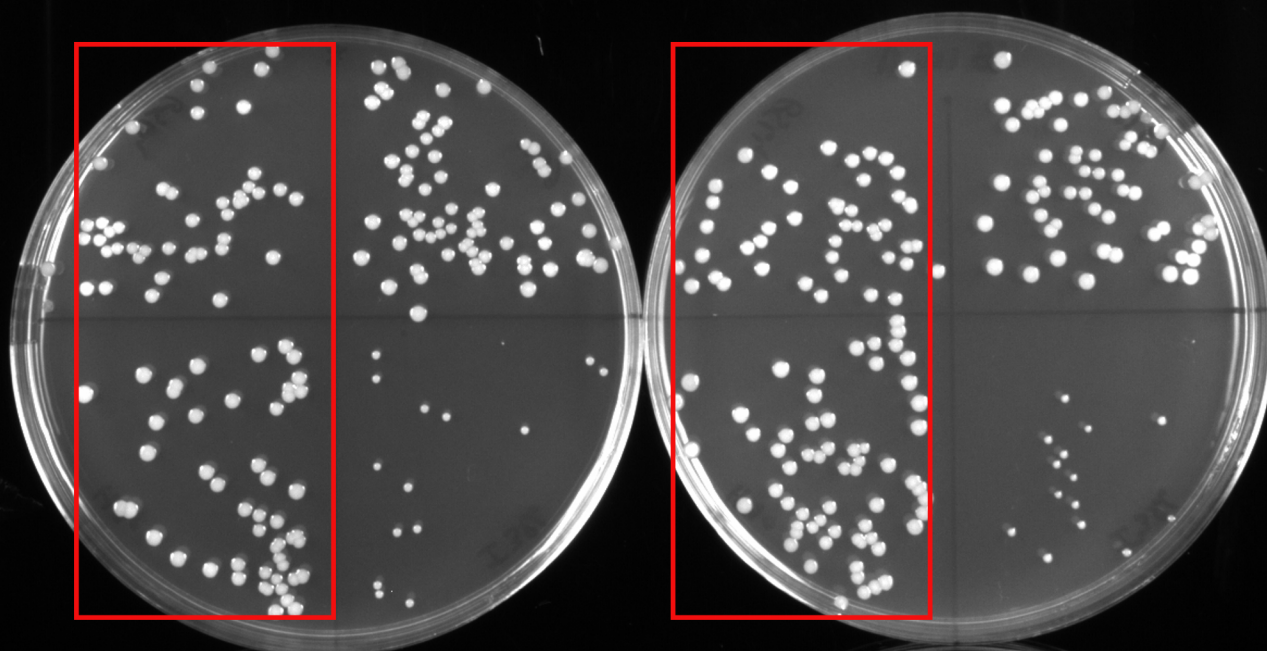

S15B Fig raw image  
LB (left part)

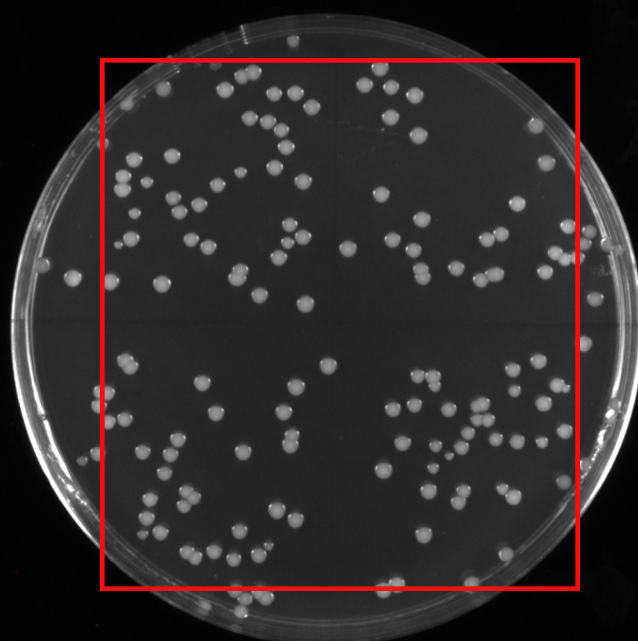

S15B Fig raw image  
LB MgCl<sub>2</sub> 10 mM (right part)

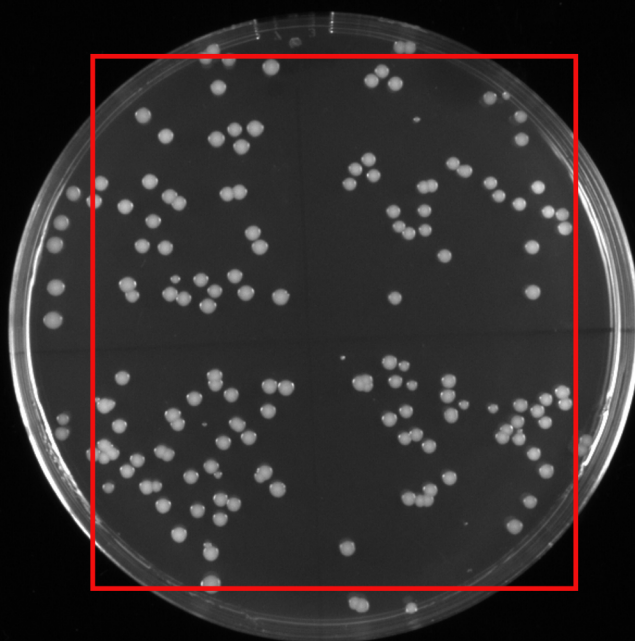

Supplement: S7 Raw images — (PDF) [file pone.0291736.s033.pdf]

S16 Fig raw image

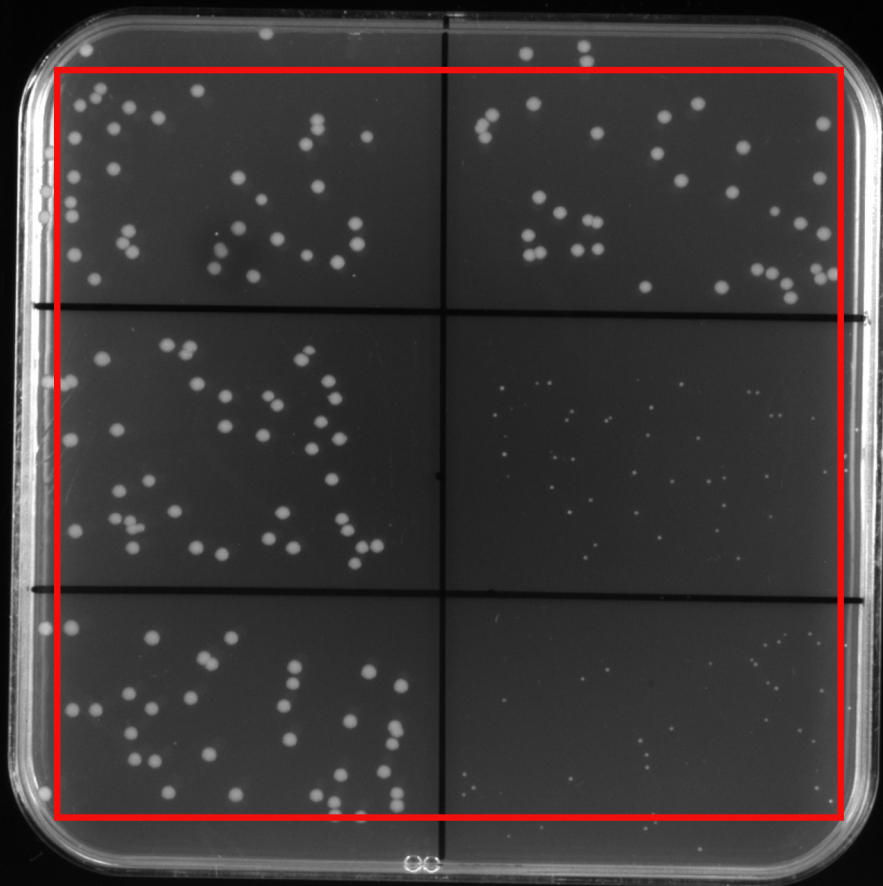

Supplement: S8 Raw images — (PDF) [file pone.0291736.s034.pdf]

S18 Fig. Raw data

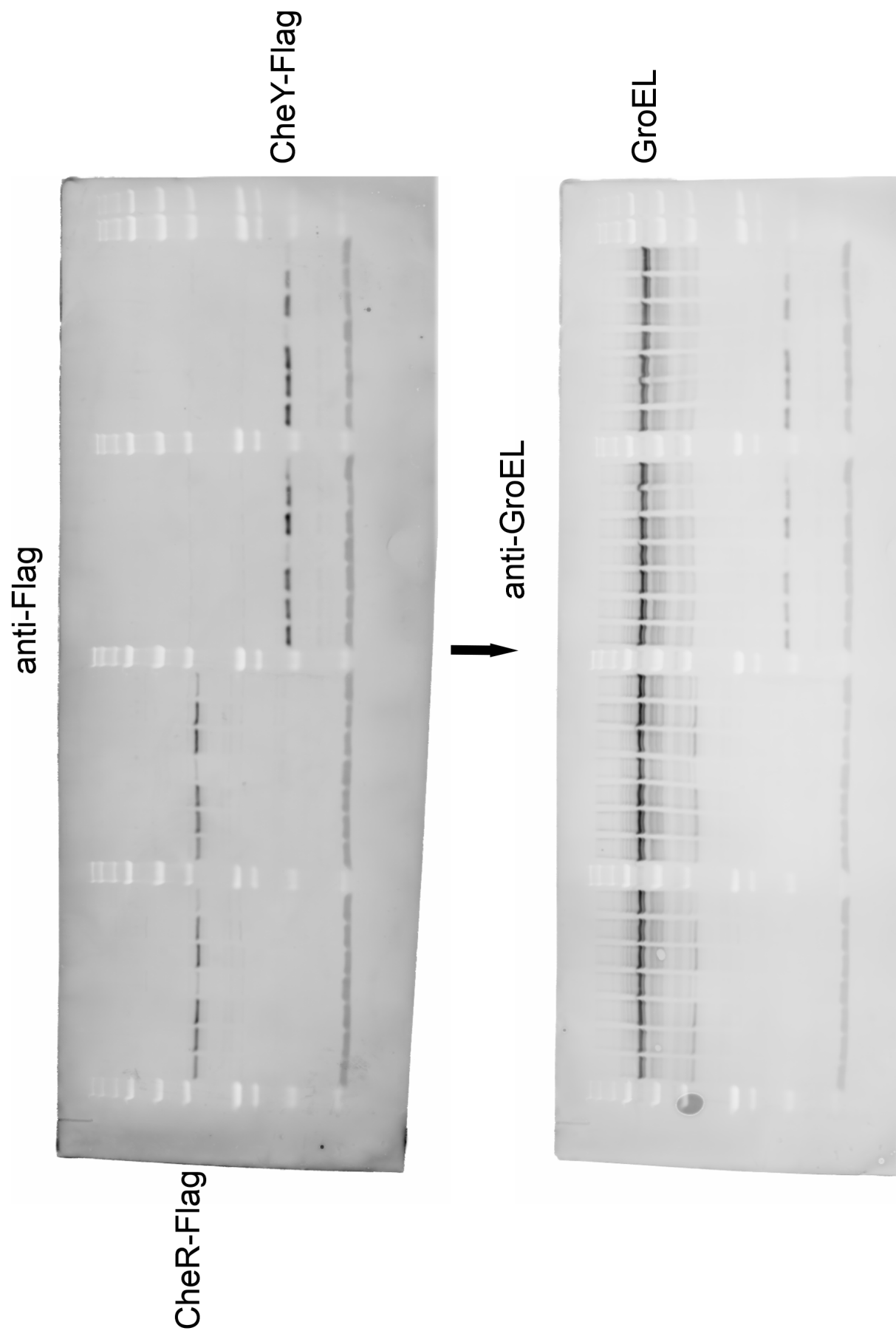

Supplement: S9 Raw images — (PDF) [file pone.0291736.s035.pdf]

Fig 1A Raw data

anti Flag

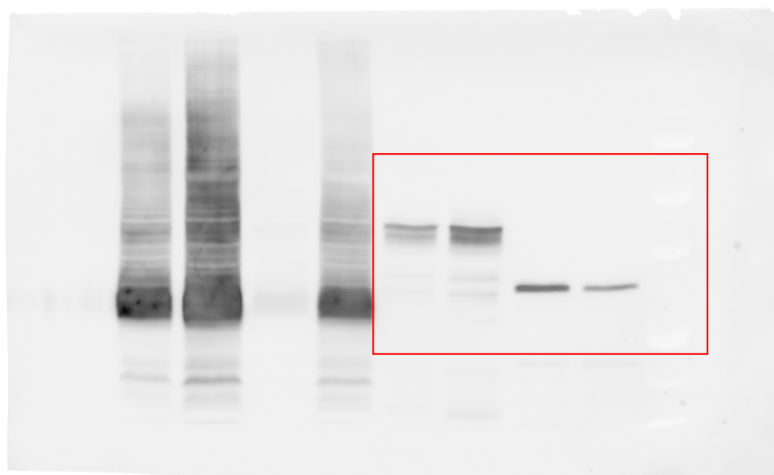

↓ anti GroEL

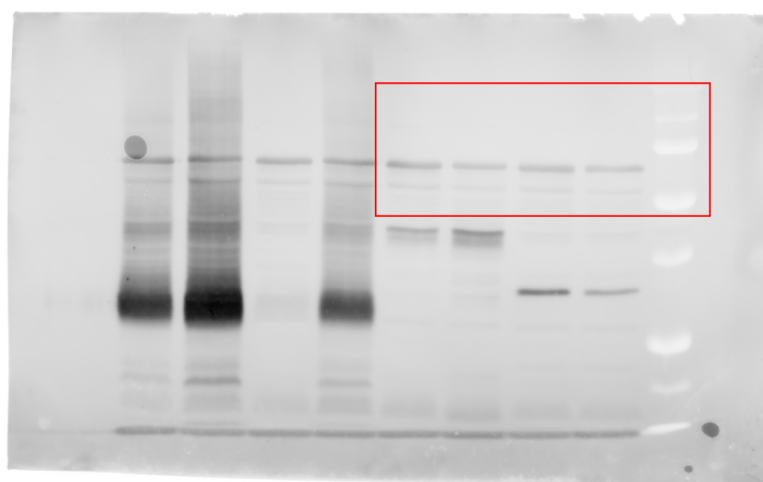

Fig 1B Raw data

anti Flag

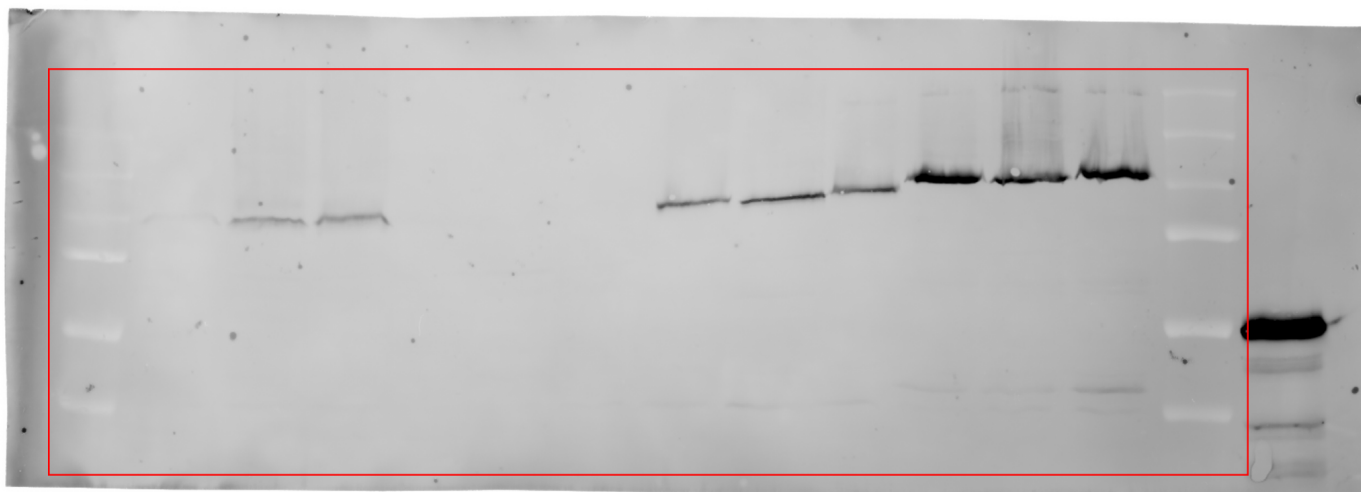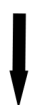

anti RNAP alpha

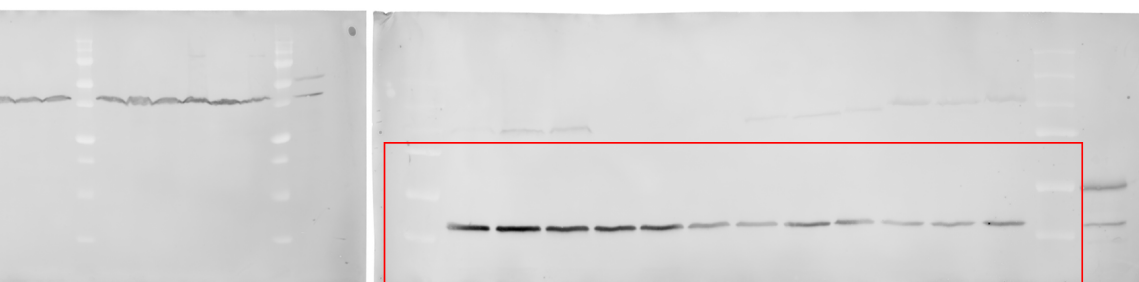

RNAP alpha

Supplement: S10 Raw images — (PDF) [file pone.0291736.s036.pdf]

Fig 4 Raw data

anti Flag

MgtA-Flag

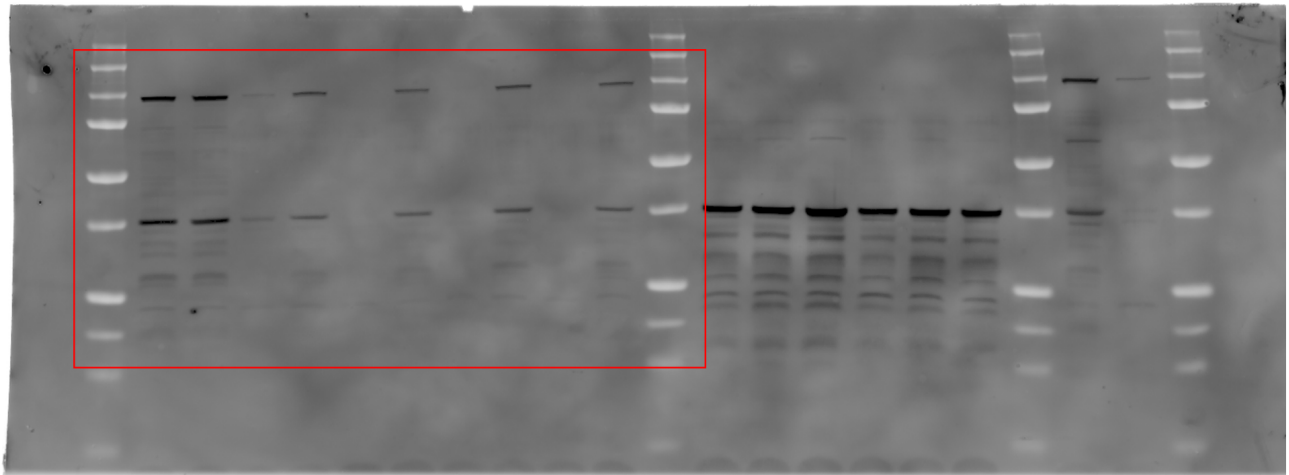

↓ anti GroEL

GroEL

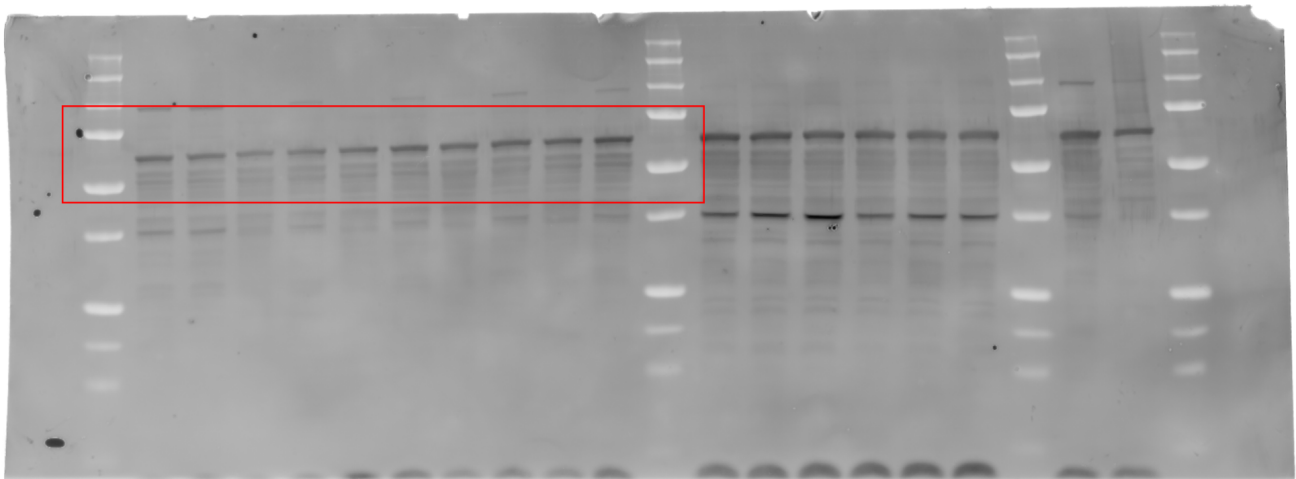

Supplement: S11 Raw images — (PDF) [file pone.0291736.s037.pdf]

Fig 5A raw data

left panel

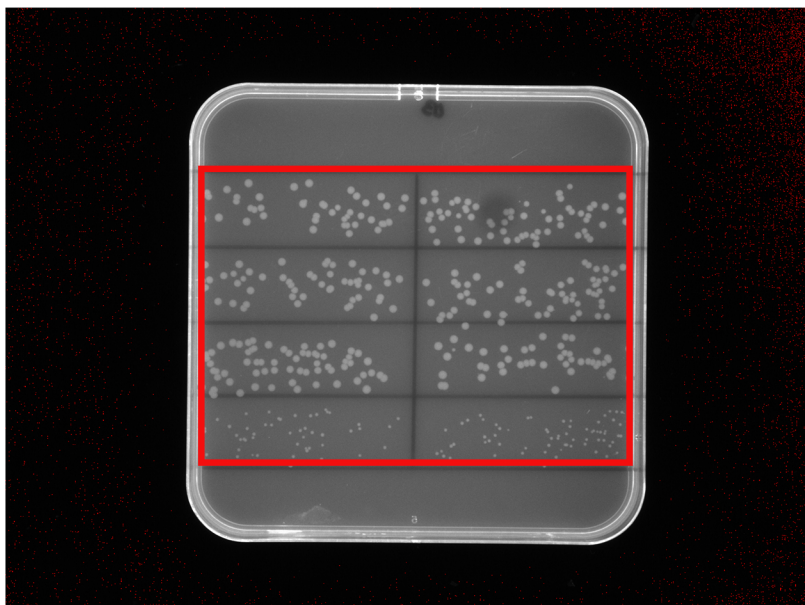

right panel

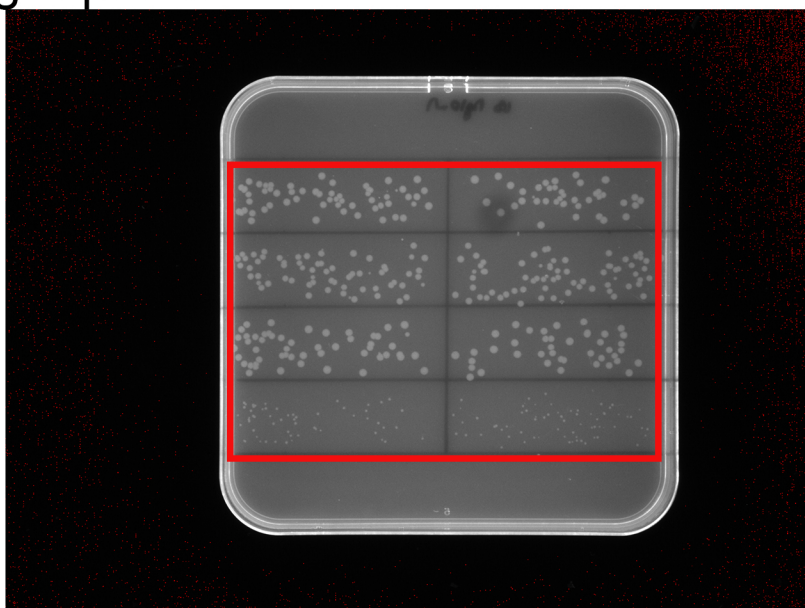

Fig 5B raw data

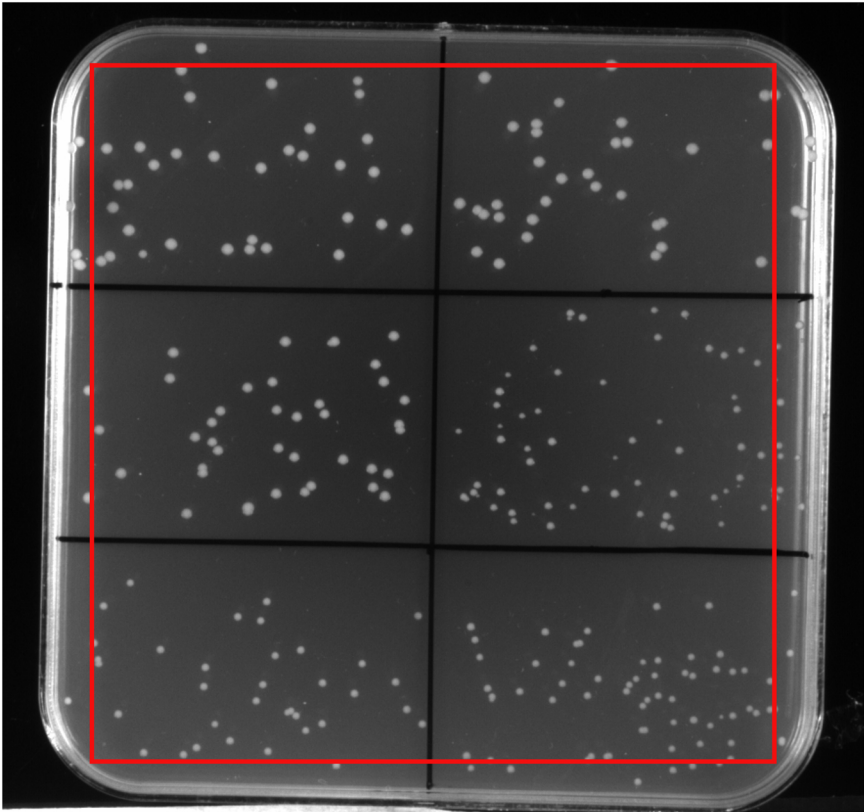

Supplement: S12 Raw images — (PDF) [file pone.0291736.s038.pdf]

Fig 7A raw image

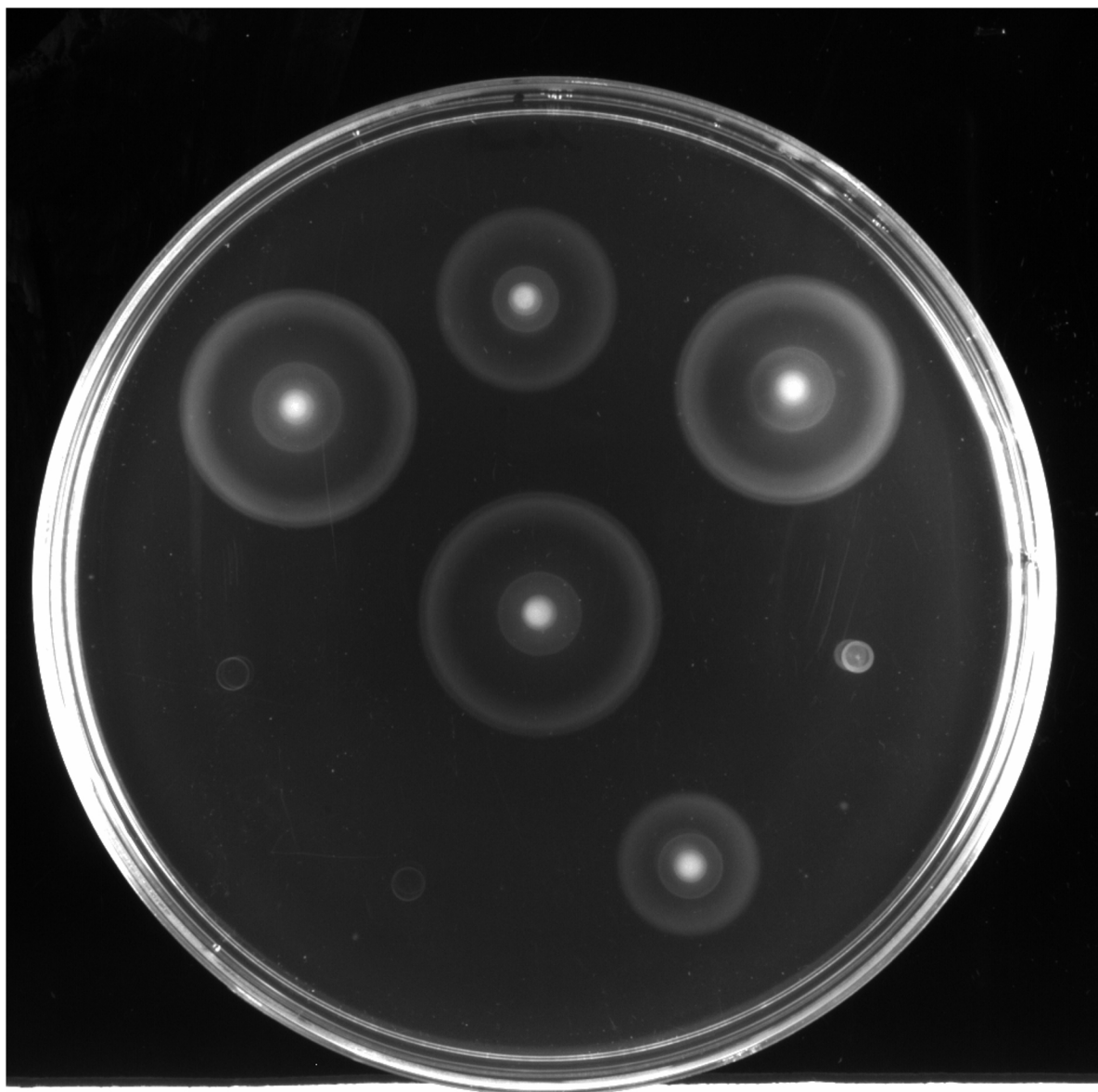

Supplement: S13 Raw images — (PDF) [file pone.0291736.s039.pdf]
