# Supplementary material for: Loss of CorA, the primary magnesium transporter of Salmonella, is alleviated by MgtA and PhoP-dependent compensatory mechanisms
Source: PLoS One. 2023 Sep 15;18(9):e0291736. doi: 10.1371/journal.pone.0291736 (PMC10503707; doi:10.1371/journal.pone.0291736)

A

#	b	b-H2O	b-NH3	a	b (2+)	Seq	y	y-H2O	y-NH3	y (2+)	#
1	115.05	97.04	98.02	87.06	58.03	N					22
2	228.13	210.12	211.11	200.14	114.57	L	2214.17	2196.16	2197.15	1107.59	21
3	341.22	323.21	324.19	313.22	171.11	L	2101.09	2083.08	2084.06	1051.04	20
4	456.25	438.24	439.22	428.25	228.62	D	1988.00	1969.99	1970.98	994.50	19
5	557.29	539.28	540.27	529.30	279.15	T	1872.98	1854.97	1855.95	936.99	18
6	628.33	610.32	611.30	600.34	314.67	A	1771.93	1753.92	1754.90	886.46	17
7	727.39	709.38	710.37	699.40	364.20	V	1700.89	1682.88	1683.87	850.95	16
8	840.46	822.46	823.46	812.49	420.74	L	1601.84	1583.81	1584.80	801.41	15
9	969.51	951.50	952.50	941.53	485.26	E	1488.75	1470.73	1471.71	744.87	14
10	1026.55	1008.54	1009.52	998.55	513.77	G	1359.70	1341.69	1342.68	680.35	13
11	1125.62	1107.60	1108.59	1097.62	563.31	V	1302.68	1284.67	1285.65	651.84	12
12	1240.64	1222.63	1223.62	1212.65	620.82	D	1203.61	1185.60	1186.58	602.30	11
13	1369.69	1351.67	1352.66	1341.69	685.34	E	1088.58	1070.57	1071.55	544.79	10
14	1470.73	1452.72	1453.71	1442.74	735.87	T	959.54	941.53	942.51	480.27	9
15	1541.77	1523.76	1524.74	1513.77	771.39	A	858.49	840.46	841.46	429.75	8
16	1612.81	1594.80	1595.78	1584.81	806.90	A	787.45	769.44	770.43	394.23	7
17	1768.91	1750.90	1751.88	1740.91	884.95	R	716.42	698.41	699.39	358.71	6
18	1896.97	1878.96	1879.94	1868.97	948.98	Q	560.32	542.30	543.28	280.66	5
19	2010.05	1992.04	1993.02	1982.06	1005.53	L	432.26	414.25	415.22	216.63	4
20	2097.08	2079.07	2080.06	2069.09	1049.04	S	319.17	301.16	302.15	160.09	3
21	2154.10	2136.09	2137.08	2126.11	1077.55	G	232.14	214.13	215.11	116.57	2
22						R	175.12	157.11	158.09	88.06	1

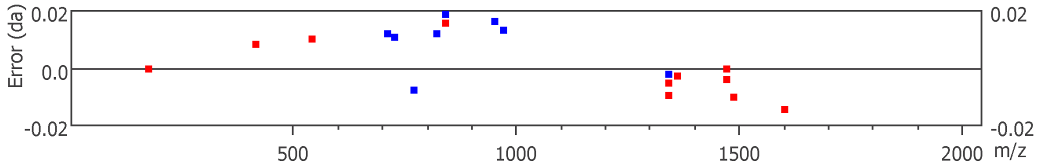

B

#	b	b-H2O	b-NH3	a	b (2+)	Seq	y	y-H2O	y-NH3	y (2+)	#
1	199.12	181.11	182.09	171.12	100.06	R(+42.01)					20
2	256.14	238.13	239.11	228.15	128.57	G	2130.11	2112.10	2113.08	1065.55	19
3	442.22	424.21	425.19	414.22	221.61	W	2073.09	2055.08	2056.06	1037.04	18
4	555.30	537.29	538.28	527.30	278.15	L	1887.01	1869.00	1869.98	944.00	17
5	626.34	608.33	609.31	598.35	313.67	A	1773.93	1755.91	1756.88	887.46	16
6	727.39	709.38	710.36	699.40	364.19	T	1702.88	1684.88	1685.86	851.94	15
7	840.47	822.46	823.45	812.48	420.74	L	1601.84	1583.83	1584.81	801.42	14
8	969.51	951.50	952.50	941.52	485.26	E	1488.75	1470.75	1471.73	744.88	13
9	1097.57	1079.56	1080.55	1069.58	549.29	Q	1359.71	1341.71	1342.69	680.36	12
10	1226.61	1208.59	1209.59	1198.62	613.81	E	1231.66	1213.63	1214.62	616.33	11
11	1325.69	1307.67	1308.66	1297.69	663.34	V	1102.61	1084.60	1085.58	551.81	10
12	1426.73	1408.72	1409.71	1398.74	713.87	T	1003.54	985.52	986.52	502.27	9
13	1539.82	1521.81	1522.79	1511.82	770.41	L	902.49	884.48	885.47	451.75	8
14	1638.89	1620.88	1621.86	1610.89	819.94	V	789.41	771.39	772.39	395.21	7
15	1753.91	1735.90	1736.89	1725.92	877.46	D	690.34	672.32	673.31	345.67	6
16	1868.94	1850.93	1851.91	1840.94	934.98	D	575.32	557.30	558.29	288.16	5
17	1969.99	1951.98	1952.96	1941.99	985.49	T	460.29	442.28	443.26	230.64	4
18	2083.07	2065.06	2066.04	2055.08	1042.04	L	359.24	341.23	342.21	180.12	3
19	2154.11	2136.10	2137.08	2126.11	1077.55	A	246.16	228.15	229.13	123.58	2
20						R	175.12	157.11	158.09	88.06	1

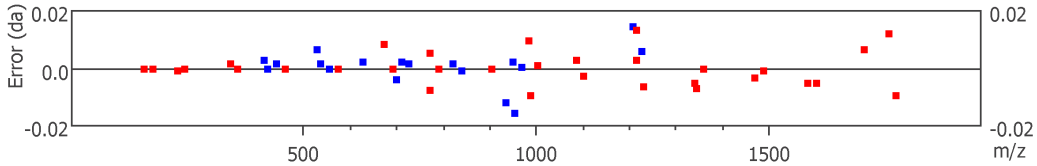

Supplement: S20 Fig — (A) MS2 ion table corresponding to S16B2 Fig. (B) MS2 ion table corresponding to S16B3 Fig (B). The database search approach attributes 20 masses to NLLDTAVLEGVDETAARQLSGR peptide. The de novo sequencing approach attributes 51 masses to a RGWLATLEQVTLVDDTLAR peptide which is not registered in the database. Orange boxes indicate similar masses between database search and de novo sequencing approaches (8 masses). See also S1 Text. (PDF) [file pone.0291736.s025.pdf]
